# Supplementary figures and images for: Inhibition of the minor spliceosome restricts the growth of a broad spectrum of cancers
Source: EMBO Rep. 2025 Jul 7;26(15):3937–69. doi: 10.1038/s44319-025-00511-8 (PMC12332006; doi:10.1038/s44319-025-00511-8)

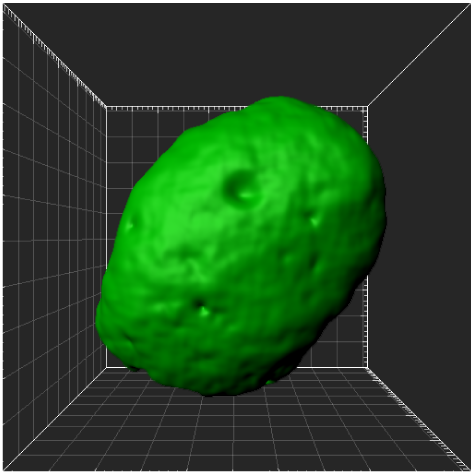

Supplement: Supplementary file 4 — Source data Fig. 1 [file 44319_2025_511_MOESM4_ESM.zip › Figure 1/Fig 1B Images/rnpc3 WT TO(kras)T 7dpf imaris.tif]

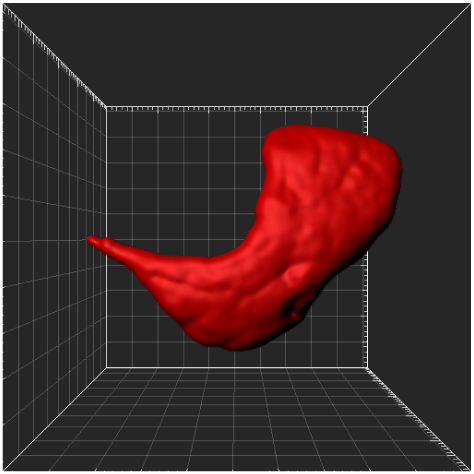

Supplement: Supplementary file 4 — Source data Fig. 1 [file 44319_2025_511_MOESM4_ESM.zip › Figure 1/Fig 1B Images/rnpc3 HET 2-CLiP 7dpf imaris.tif]

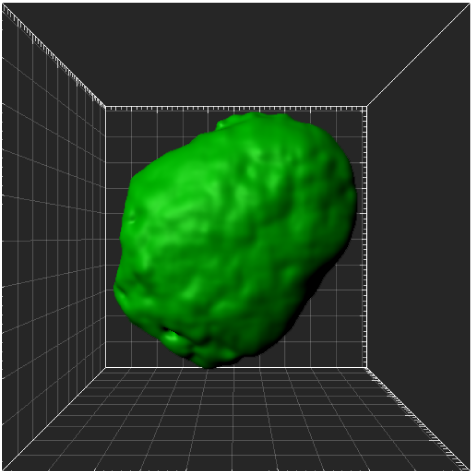

Supplement: Supplementary file 4 — Source data Fig. 1 [file 44319_2025_511_MOESM4_ESM.zip › Figure 1/Fig 1B Images/rnpc3 HET TO(kras)T 7dpf imaris.tif]

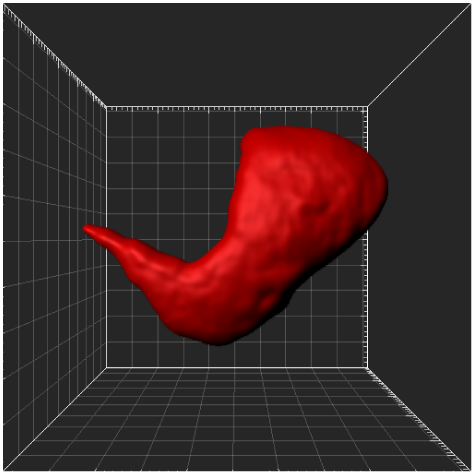

Supplement: Supplementary file 4 — Source data Fig. 1 [file 44319_2025_511_MOESM4_ESM.zip › Figure 1/Fig 1B Images/rnpc3 WT 2-CLiP 7dpf imaris.tif]

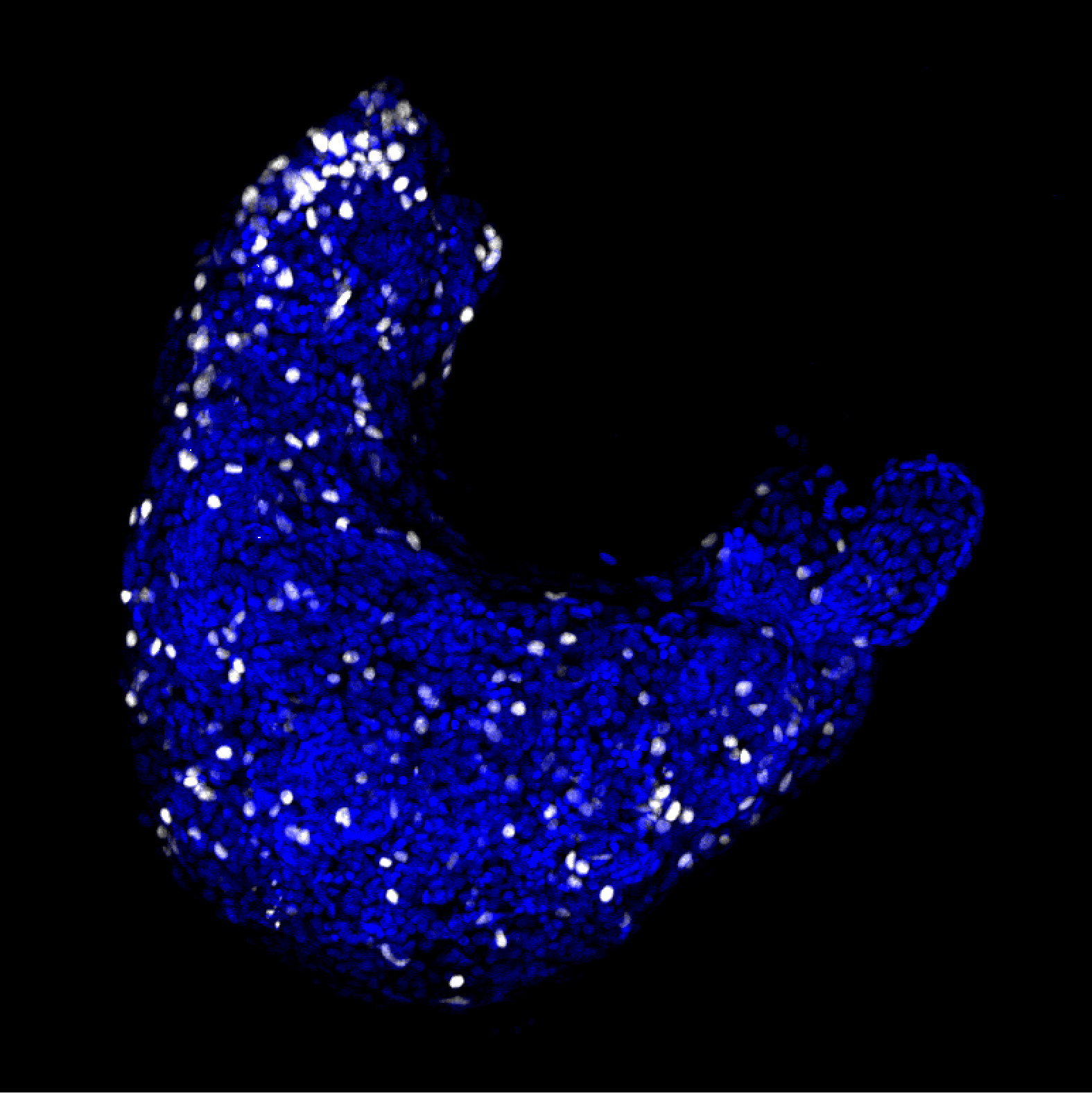

Supplement: Supplementary file 4 — Source data Fig. 1 [file 44319_2025_511_MOESM4_ESM.zip › Figure 1/Fig 1D Images/Representative rnpc3 HET TO(kras)T+ 7dpf EdU overlay.tif]

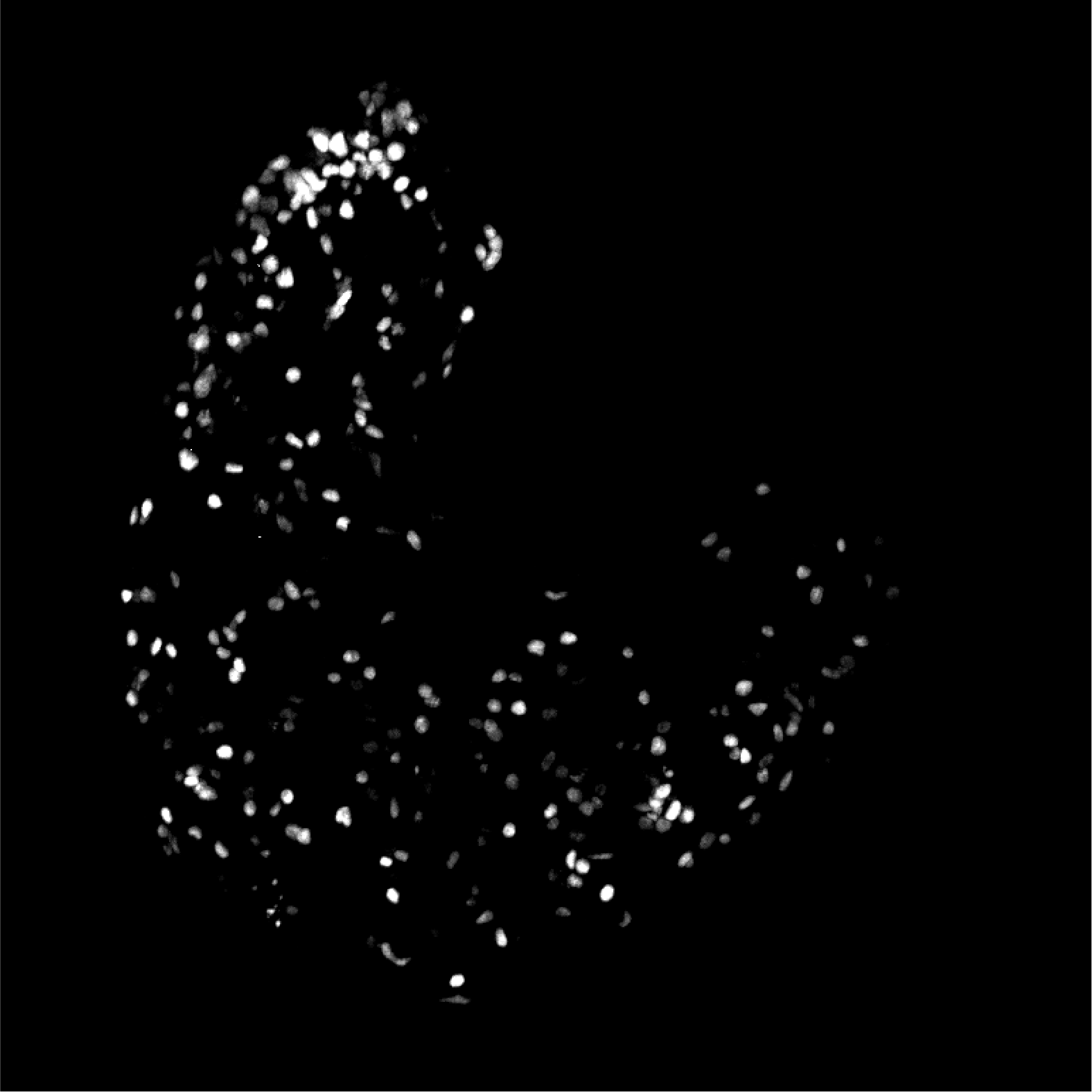

Supplement: Supplementary file 4 — Source data Fig. 1 [file 44319_2025_511_MOESM4_ESM.zip › Figure 1/Fig 1D Images/Representative rnpc3 HET TO(kras)T+ 7dpf EdU white.tif]

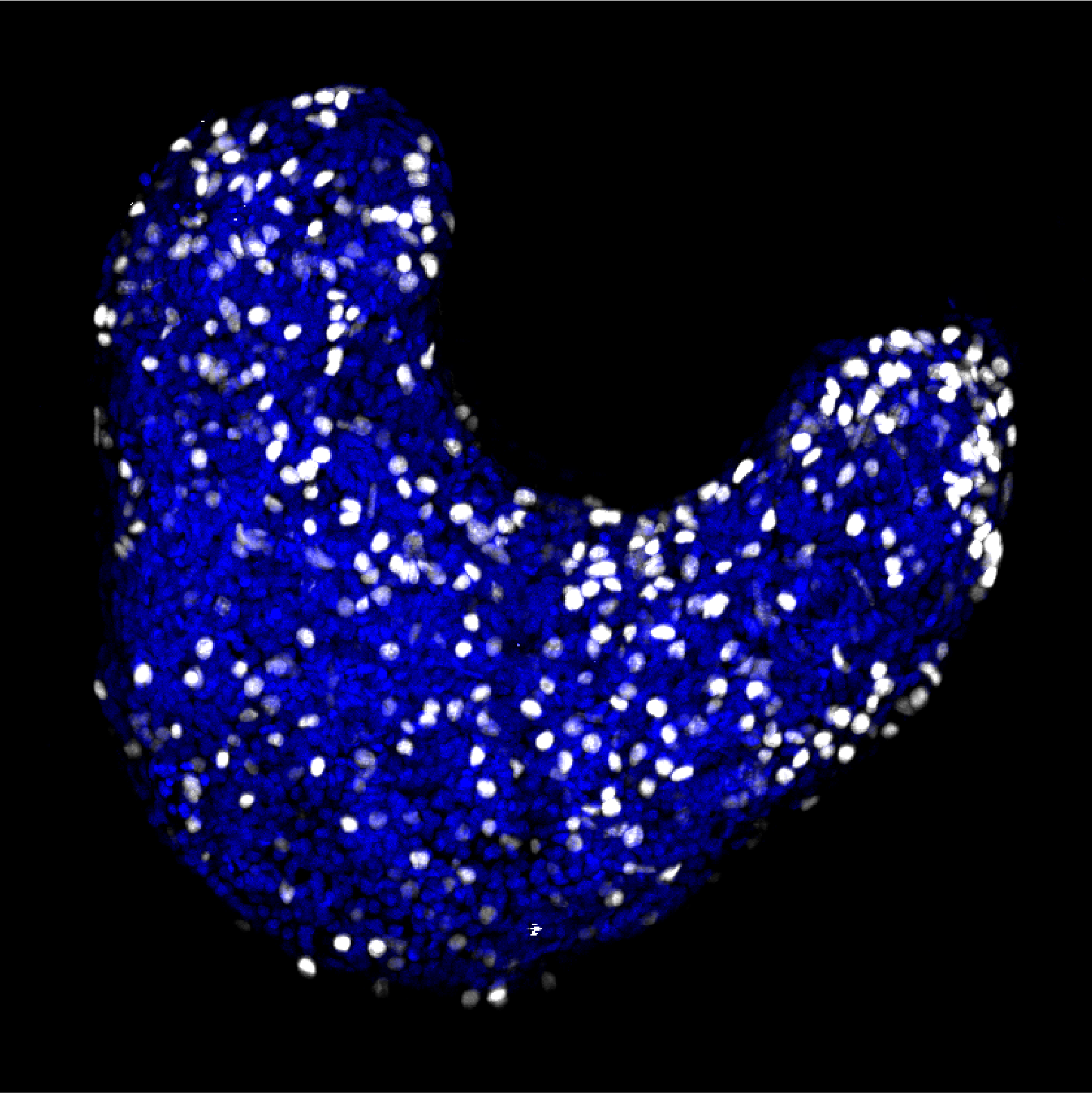

Supplement: Supplementary file 4 — Source data Fig. 1 [file 44319_2025_511_MOESM4_ESM.zip › Figure 1/Fig 1D Images/Representative rnpc3 WT TO(kras)T+ 7dpf EdU overlay.tif]

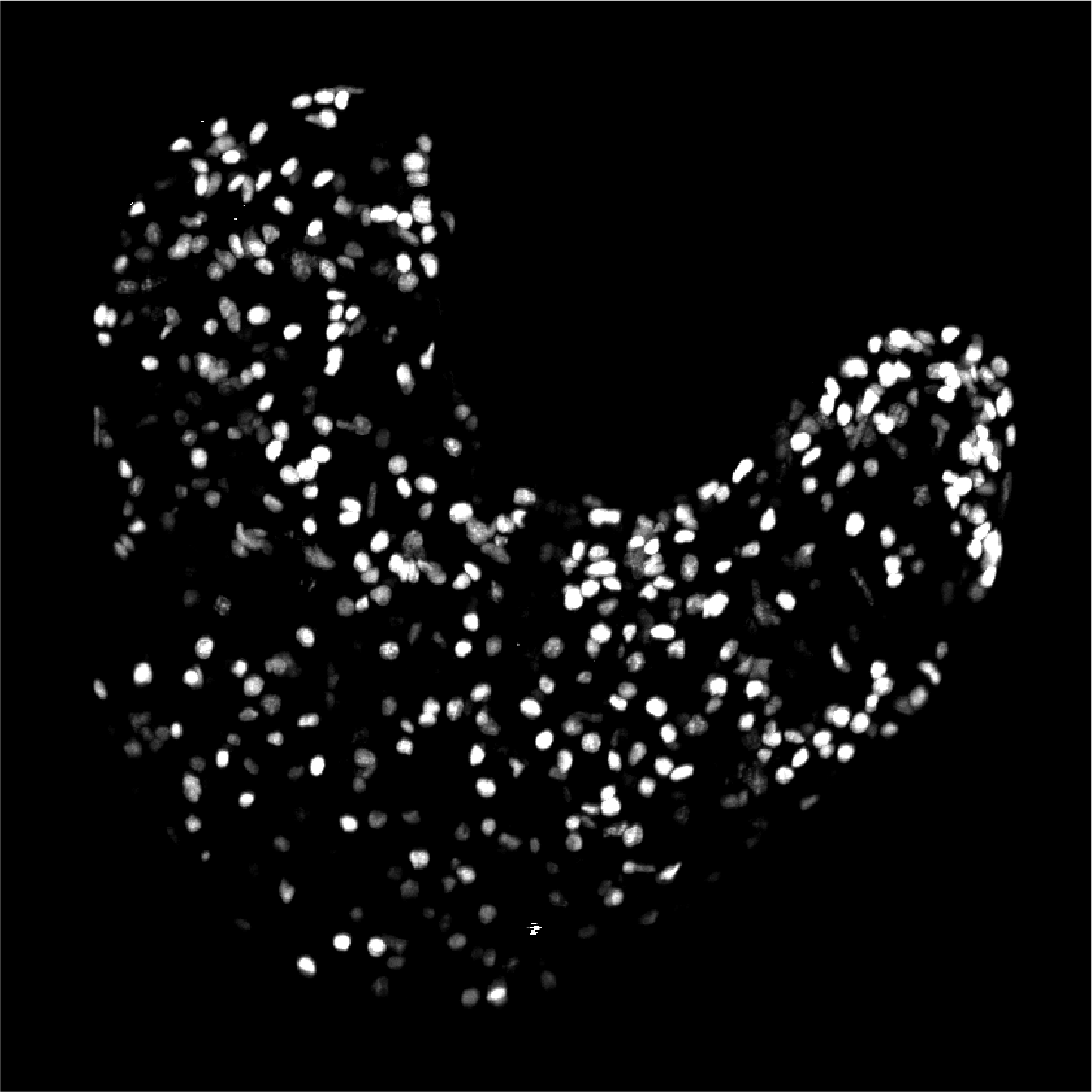

Supplement: Supplementary file 4 — Source data Fig. 1 [file 44319_2025_511_MOESM4_ESM.zip › Figure 1/Fig 1D Images/Representative rnpc3 WT TO(kras)T+ 7dpf EdU white.tif]

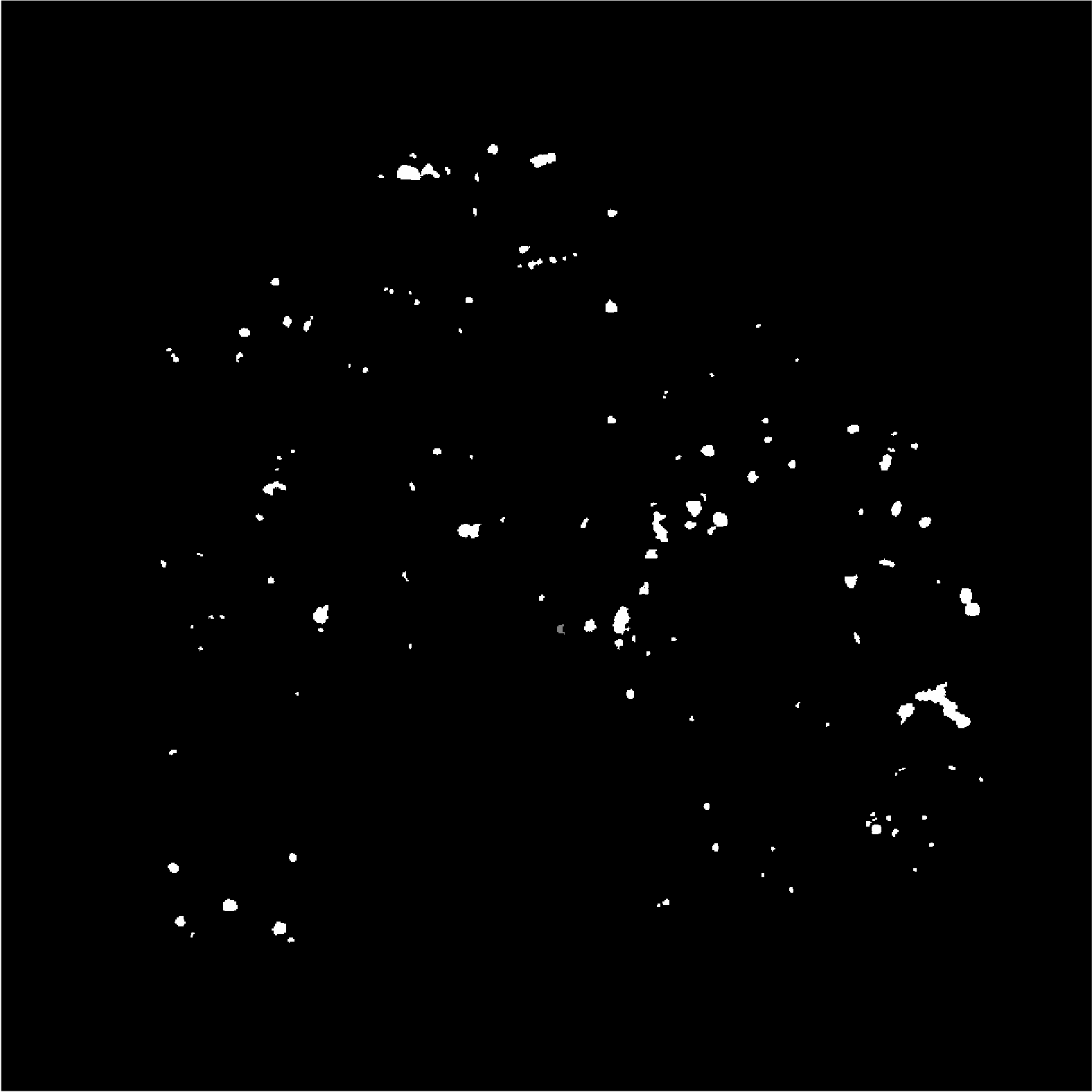

Supplement: Supplementary file 4 — Source data Fig. 1 [file 44319_2025_511_MOESM4_ESM.zip › Figure 1/Fig 1F Images/Representative rnpc3 HET TO(kras)T+ 7dpf Annexin white.tif]

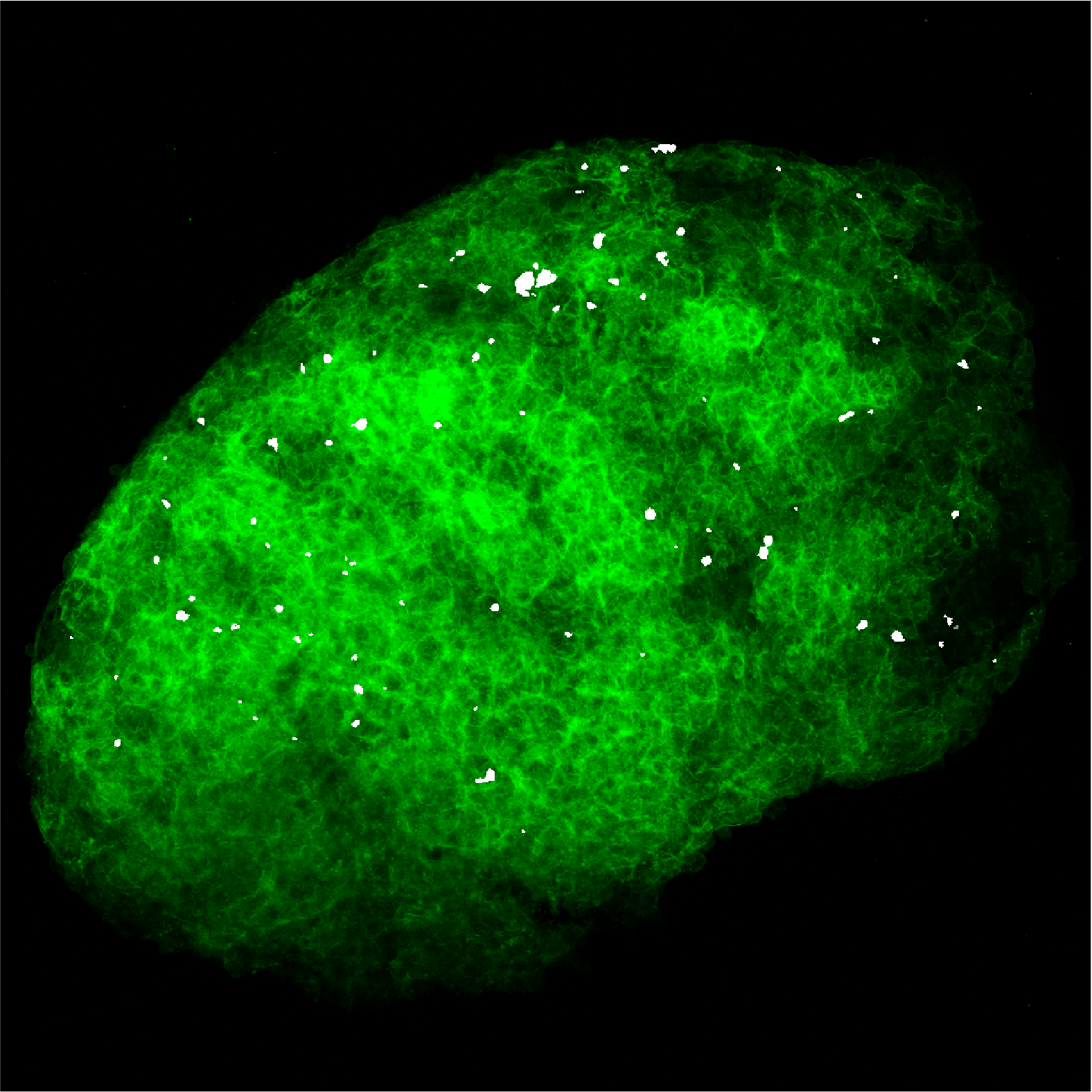

Supplement: Supplementary file 4 — Source data Fig. 1 [file 44319_2025_511_MOESM4_ESM.zip › Figure 1/Fig 1F Images/Representative rnpc3 WT TO(kras)T+ 7dpf Annexin overlay.tif]

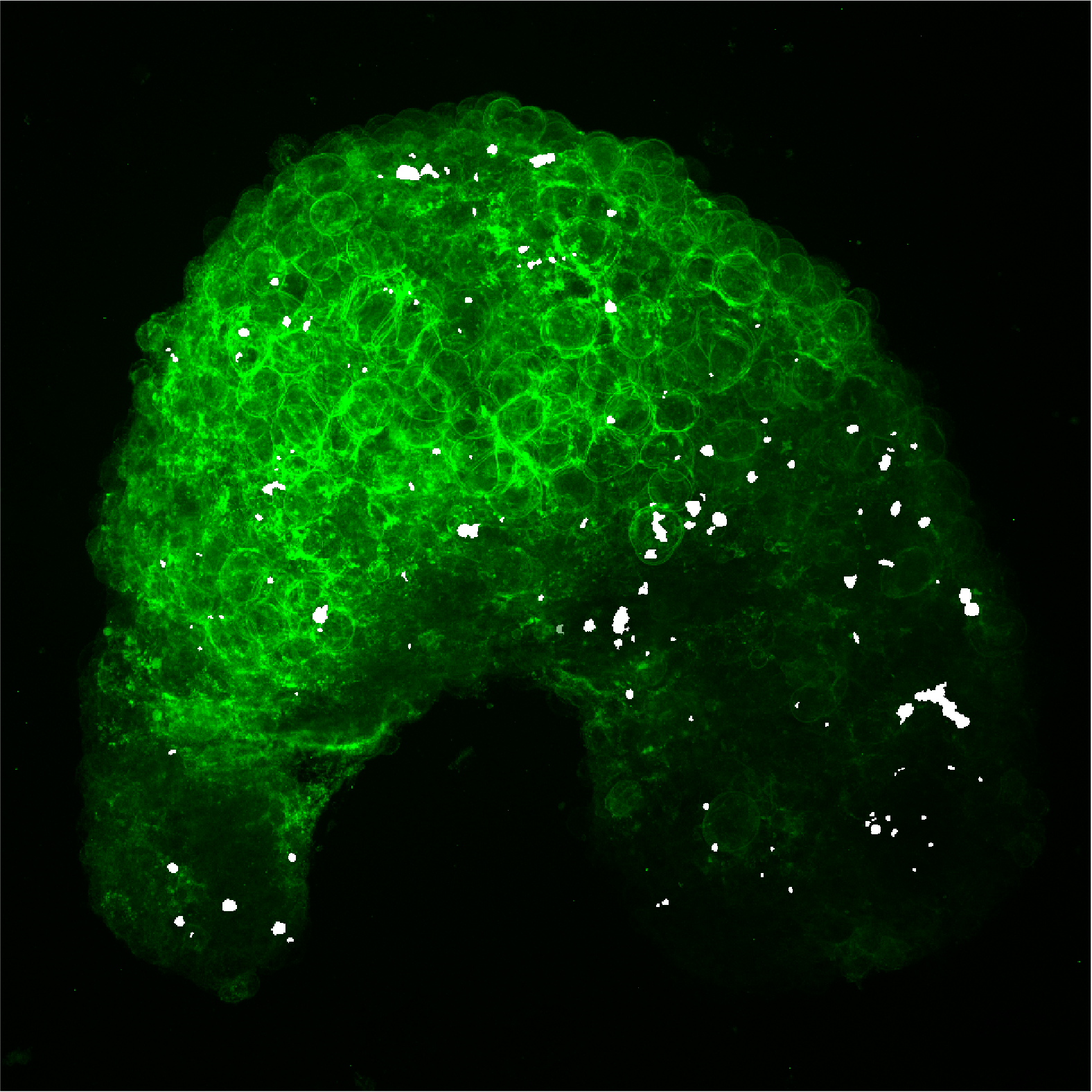

Supplement: Supplementary file 4 — Source data Fig. 1 [file 44319_2025_511_MOESM4_ESM.zip › Figure 1/Fig 1F Images/Representative rnpc3 HET TO(kras)T+ 7dpf Annexin overlay.tif]

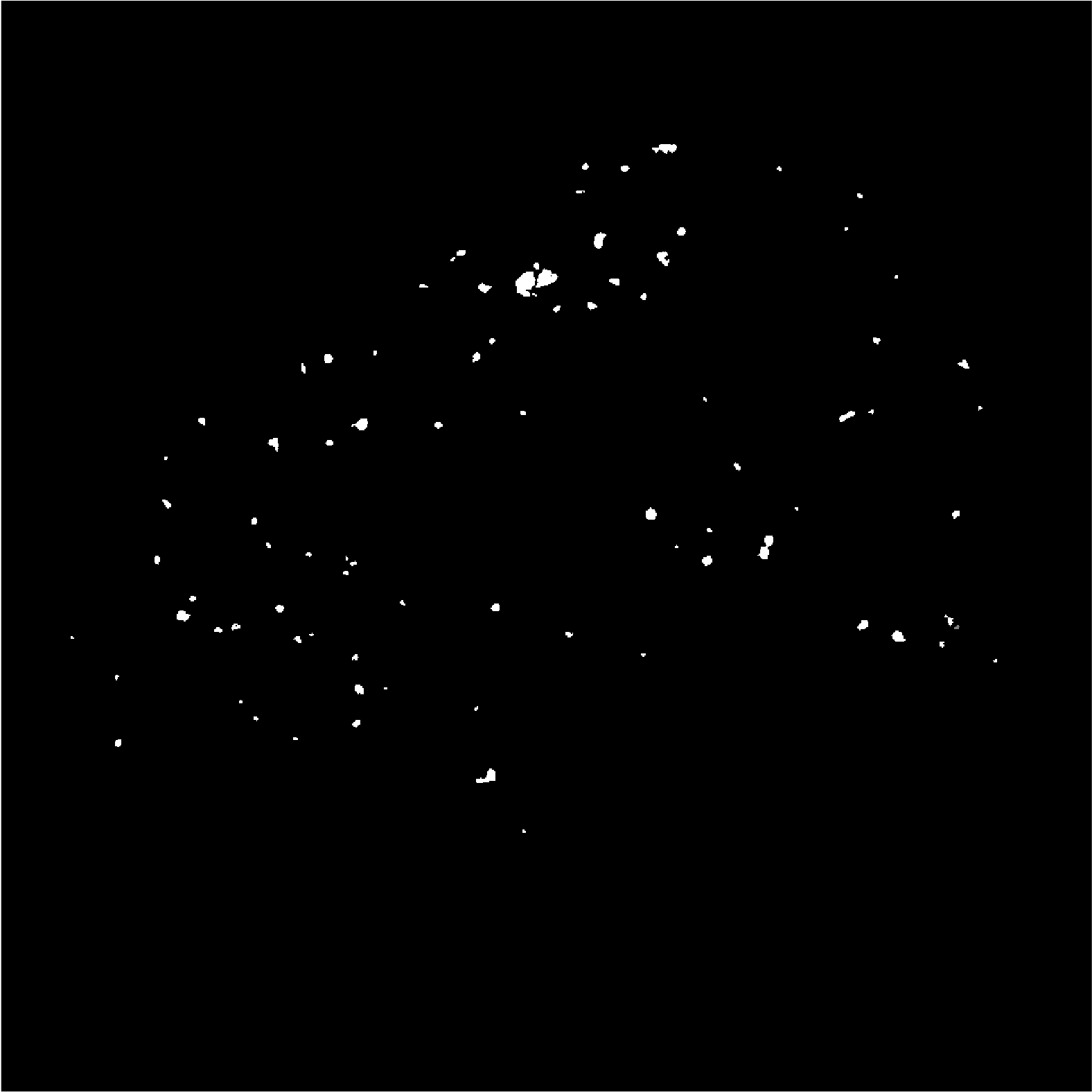

Supplement: Supplementary file 4 — Source data Fig. 1 [file 44319_2025_511_MOESM4_ESM.zip › Figure 1/Fig 1F Images/Representative rnpc3 WT TO(kras)T+ 7dpf Annexin white.tif]

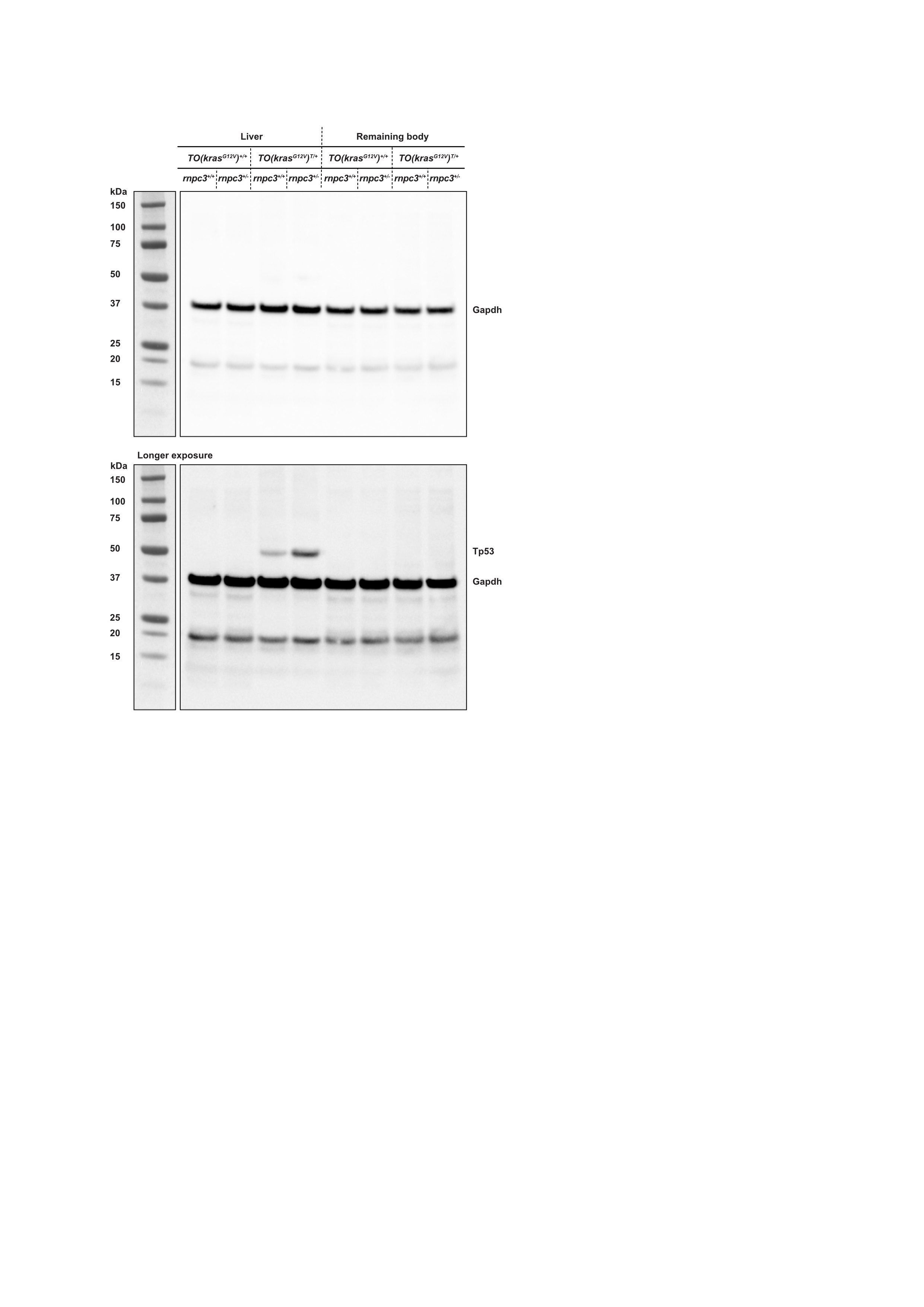

Supplement: Supplementary file 5 — Source data Fig. 2 [file 44319_2025_511_MOESM5_ESM.zip › Figure 2/Fig 2A Western.tiff]

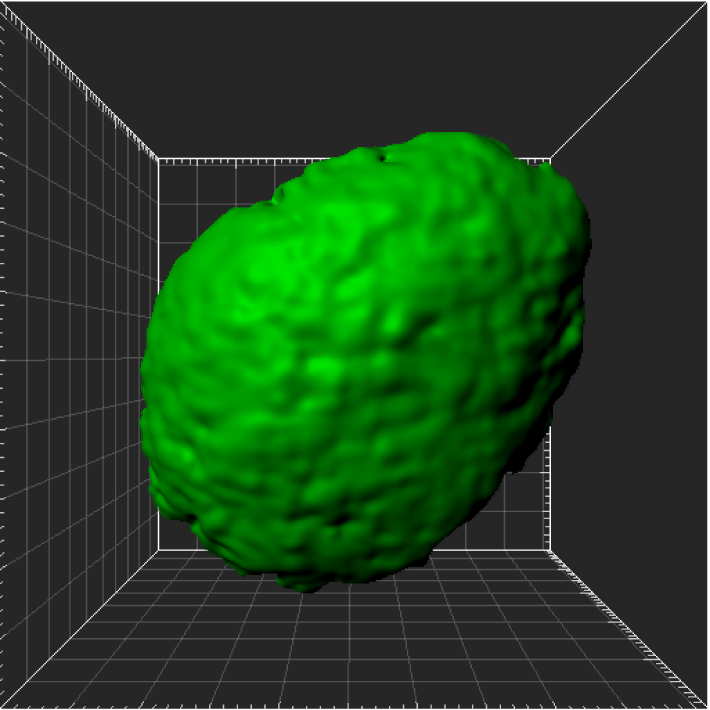

Supplement: Supplementary file 5 — Source data Fig. 2 [file 44319_2025_511_MOESM5_ESM.zip › Figure 2/Fig 2B Images/rnpc3 HET tp53 MUT TO(kras).tif]

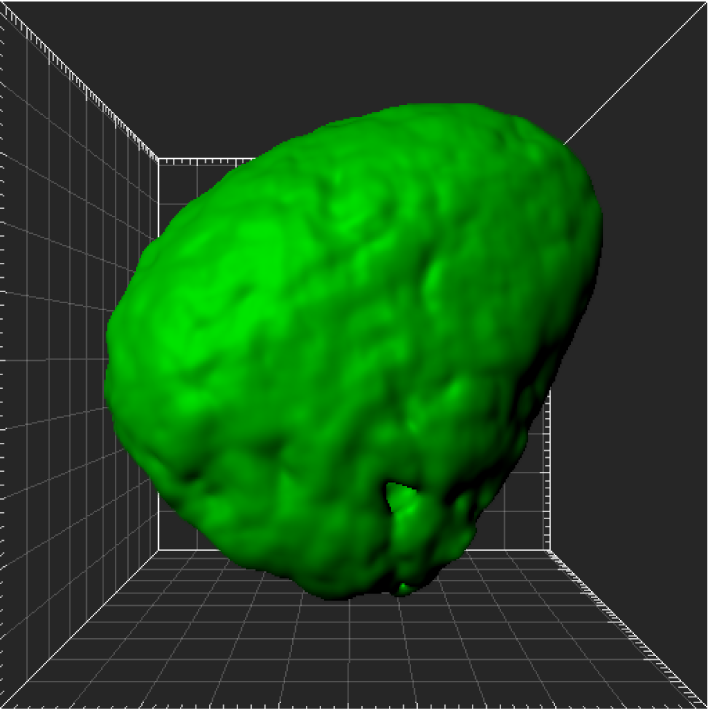

Supplement: Supplementary file 5 — Source data Fig. 2 [file 44319_2025_511_MOESM5_ESM.zip › Figure 2/Fig 2B Images/rnpc3 WT tp53 MUT TO(kras).tif]

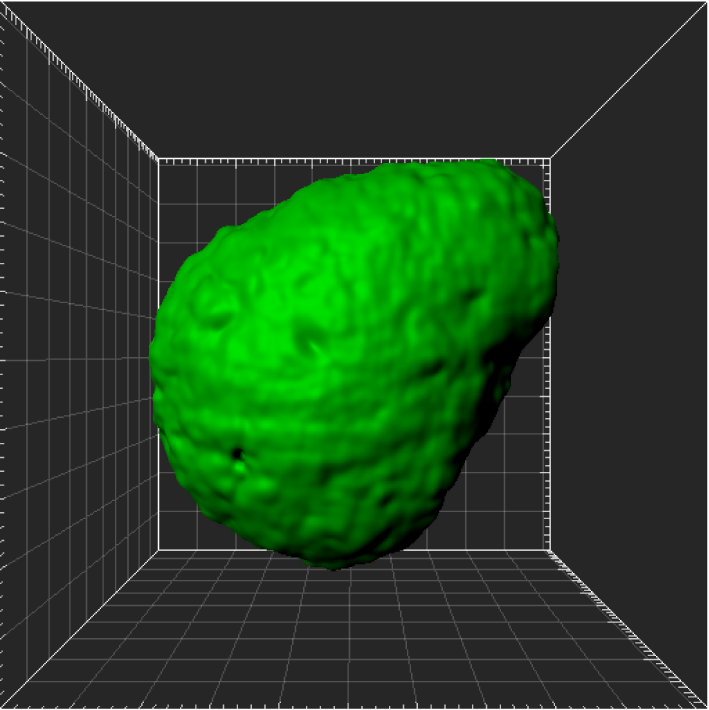

Supplement: Supplementary file 5 — Source data Fig. 2 [file 44319_2025_511_MOESM5_ESM.zip › Figure 2/Fig 2B Images/rnpc3 WT tp53 WT TO(kras).tif]

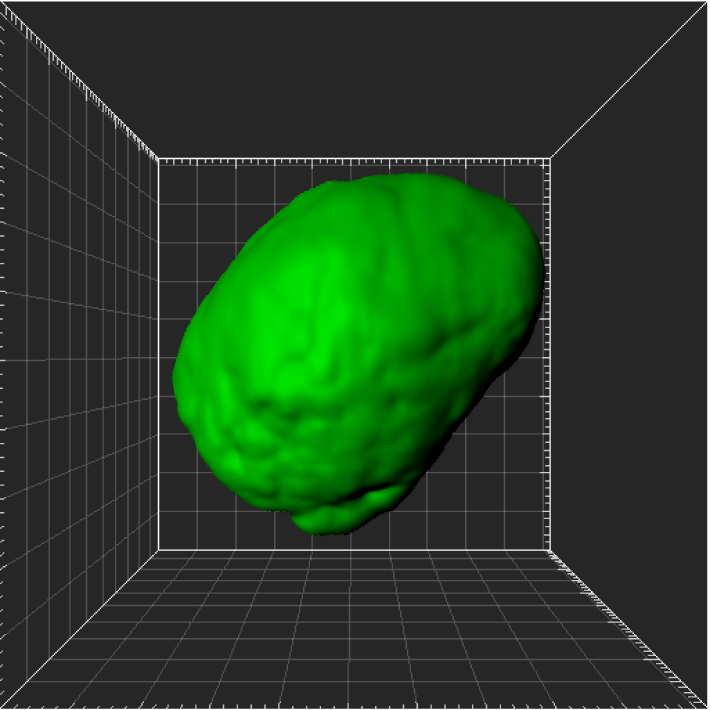

Supplement: Supplementary file 5 — Source data Fig. 2 [file 44319_2025_511_MOESM5_ESM.zip › Figure 2/Fig 2B Images/rnpc3 HET tp53 WT TO(kras).tif]

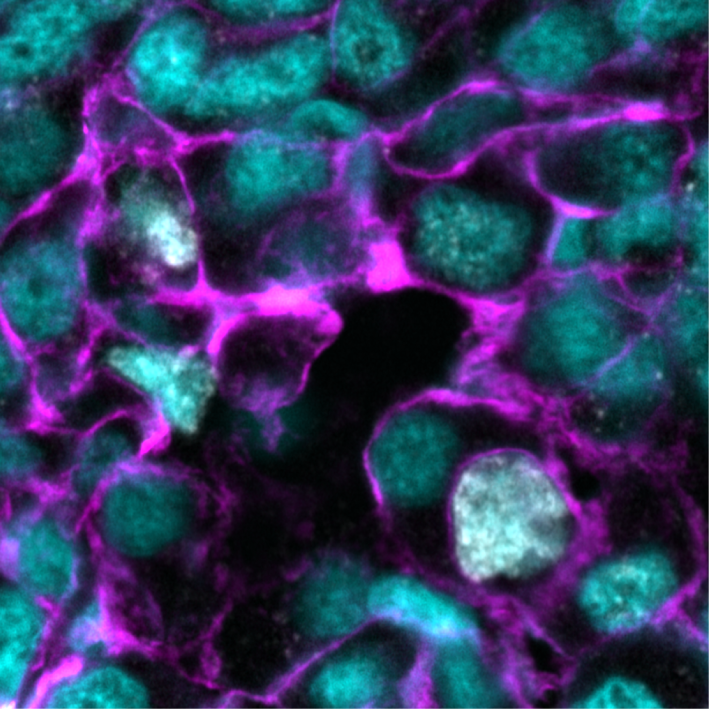

Supplement: Supplementary file 5 — Source data Fig. 2 [file 44319_2025_511_MOESM5_ESM.zip › Figure 2/Fig 2L Images/rnpc3 HET tp53 WT H2AX merge.tif]

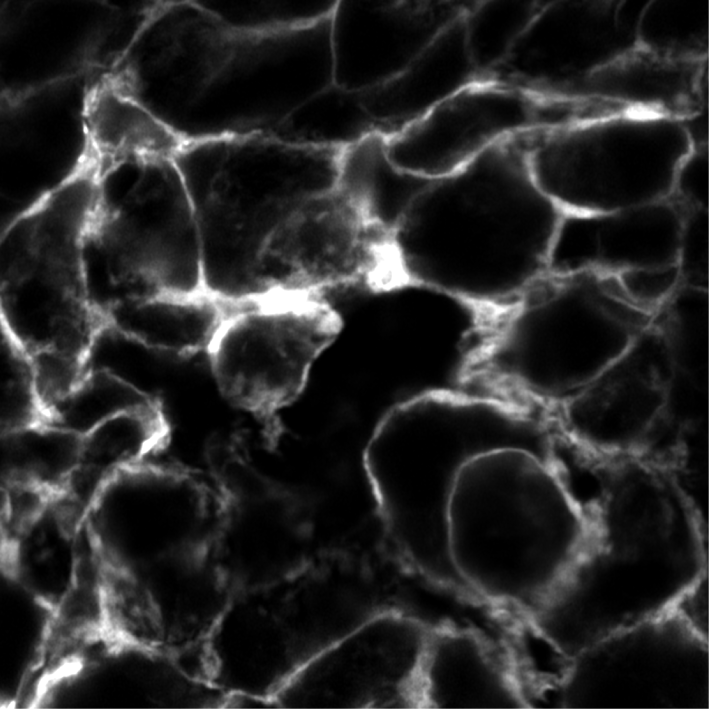

Supplement: Supplementary file 5 — Source data Fig. 2 [file 44319_2025_511_MOESM5_ESM.zip › Figure 2/Fig 2L Images/rnpc3 HET tp53 WT H2AX GFP.tif]

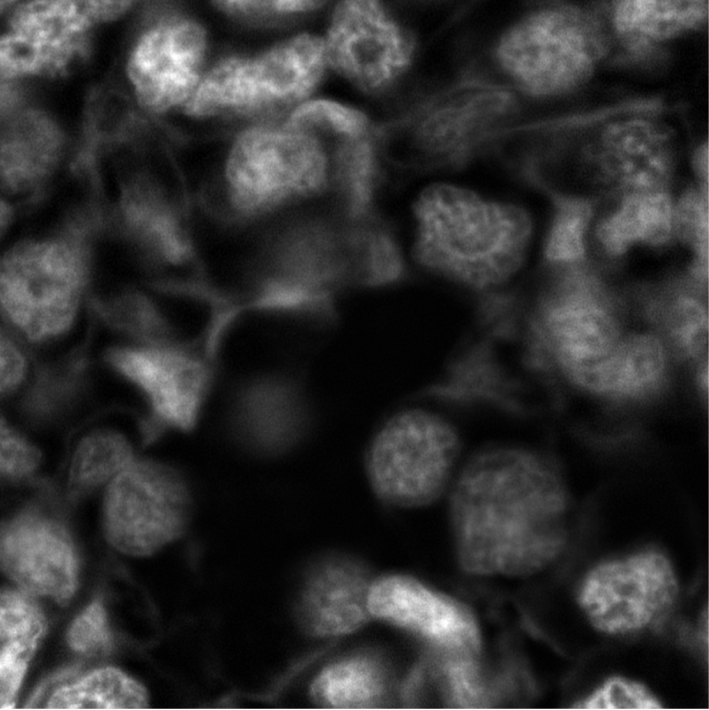

Supplement: Supplementary file 5 — Source data Fig. 2 [file 44319_2025_511_MOESM5_ESM.zip › Figure 2/Fig 2L Images/rnpc3 HET tp53 WT H2AX DAPI.tif]

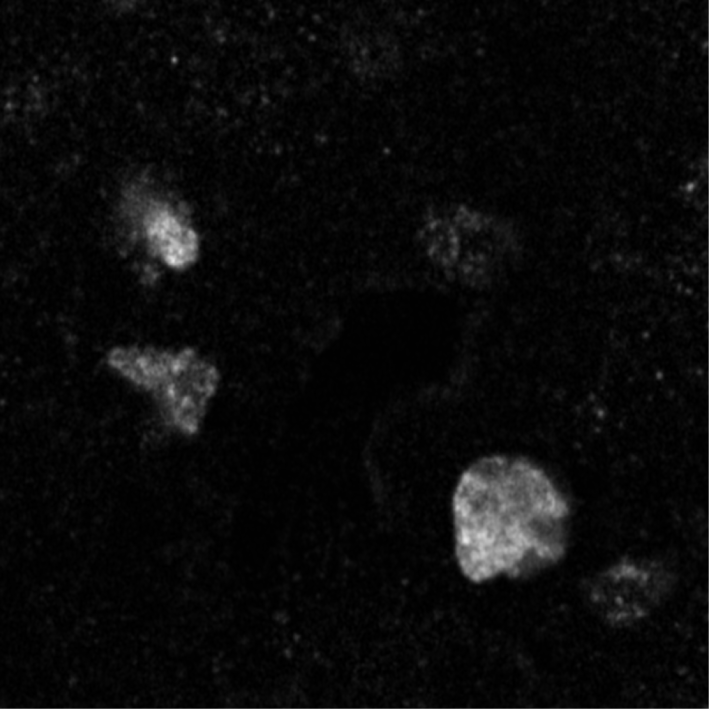

Supplement: Supplementary file 5 — Source data Fig. 2 [file 44319_2025_511_MOESM5_ESM.zip › Figure 2/Fig 2L Images/rnpc3 HET tp53 WT H2AX gH2AX.tif]

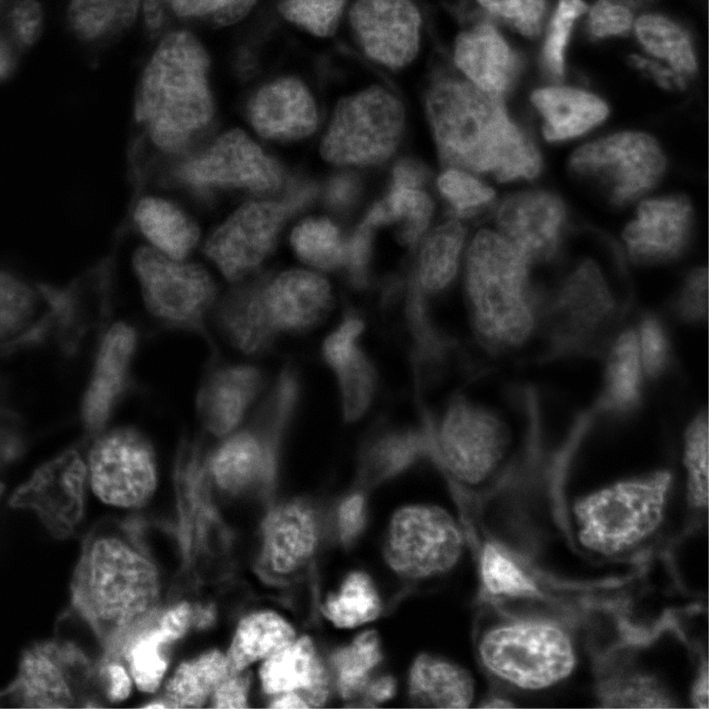

Supplement: Supplementary file 5 — Source data Fig. 2 [file 44319_2025_511_MOESM5_ESM.zip › Figure 2/Fig 2L Images/rnpc3 HET tp53 MUT H2AX DAPI.tif]

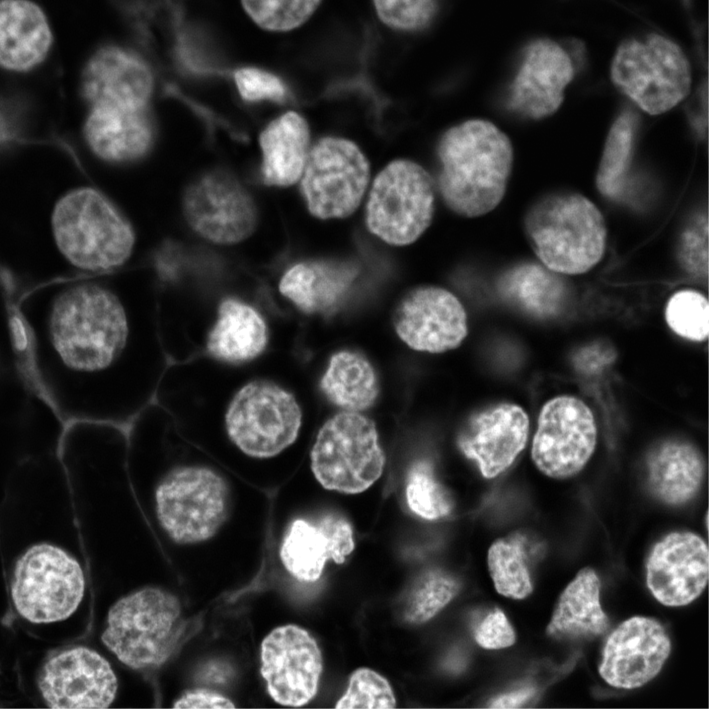

Supplement: Supplementary file 5 — Source data Fig. 2 [file 44319_2025_511_MOESM5_ESM.zip › Figure 2/Fig 2L Images/rnpc3 WT tp53 WT H2AX DAPI.tif]

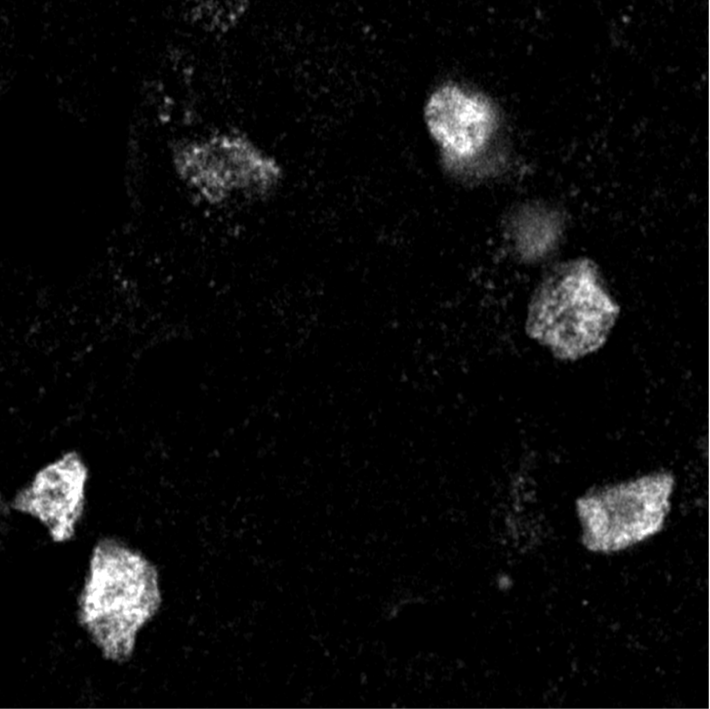

Supplement: Supplementary file 5 — Source data Fig. 2 [file 44319_2025_511_MOESM5_ESM.zip › Figure 2/Fig 2L Images/rnpc3 HET tp53 MUT H2AX gH2AX.tif]

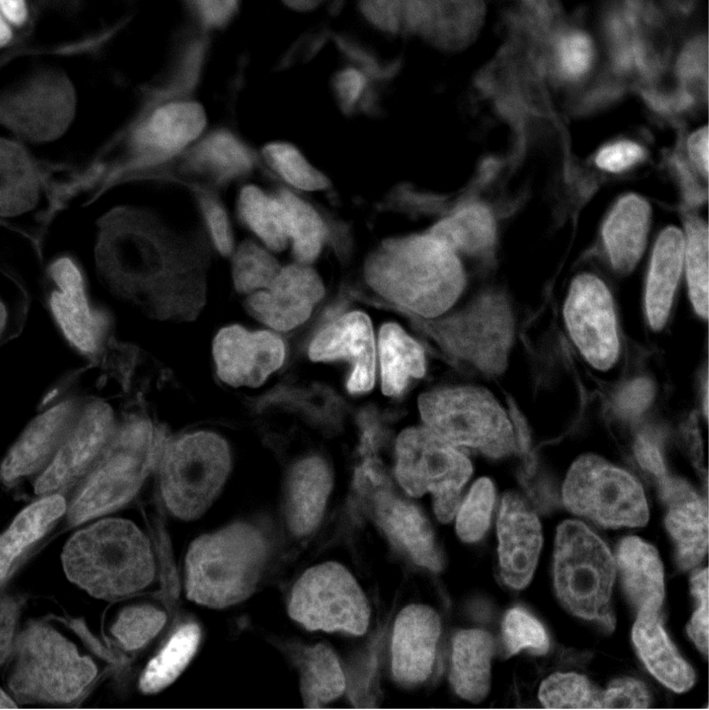

Supplement: Supplementary file 5 — Source data Fig. 2 [file 44319_2025_511_MOESM5_ESM.zip › Figure 2/Fig 2L Images/rnpc3 WT tp53 MUT H2AX DAPI.tif]

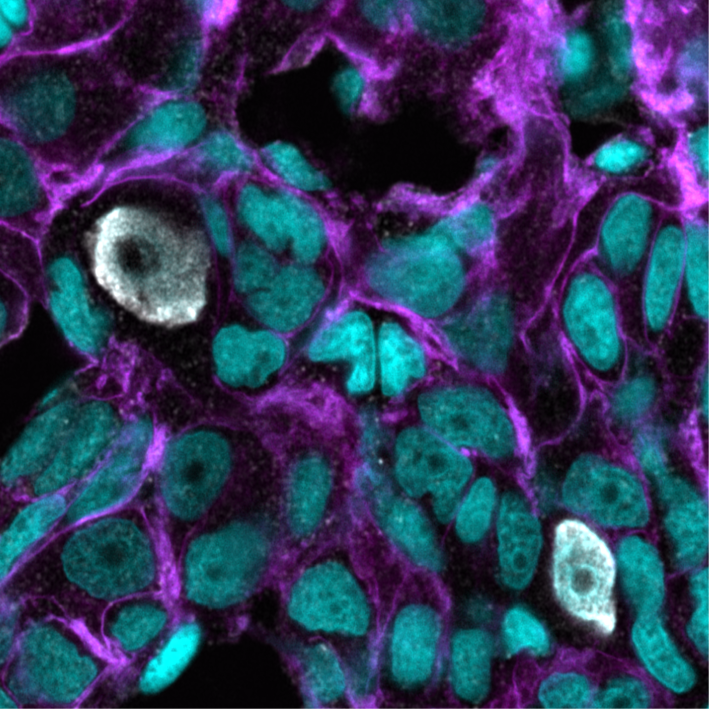

Supplement: Supplementary file 5 — Source data Fig. 2 [file 44319_2025_511_MOESM5_ESM.zip › Figure 2/Fig 2L Images/rnpc3 WT tp53 MUT H2AX merge.tif]

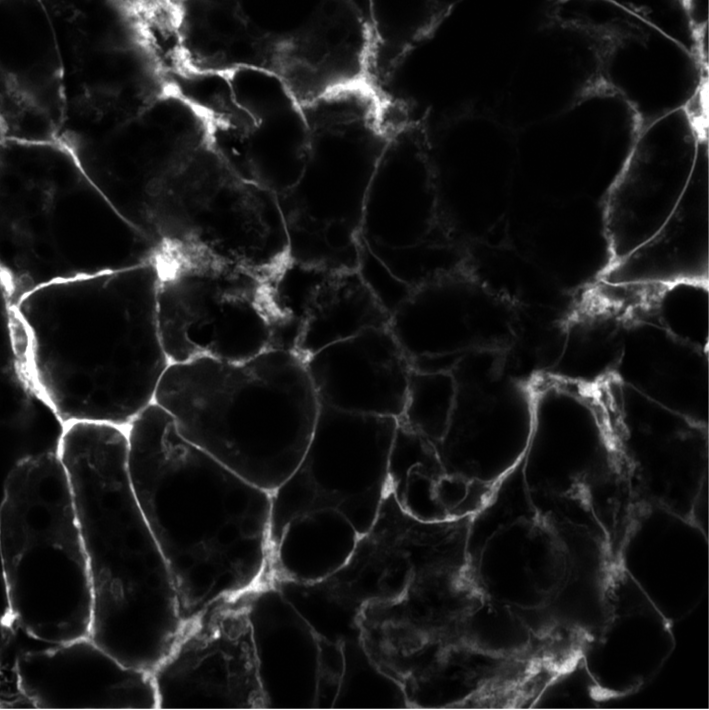

Supplement: Supplementary file 5 — Source data Fig. 2 [file 44319_2025_511_MOESM5_ESM.zip › Figure 2/Fig 2L Images/rnpc3 WT tp53 WT H2AX GFP.tif]

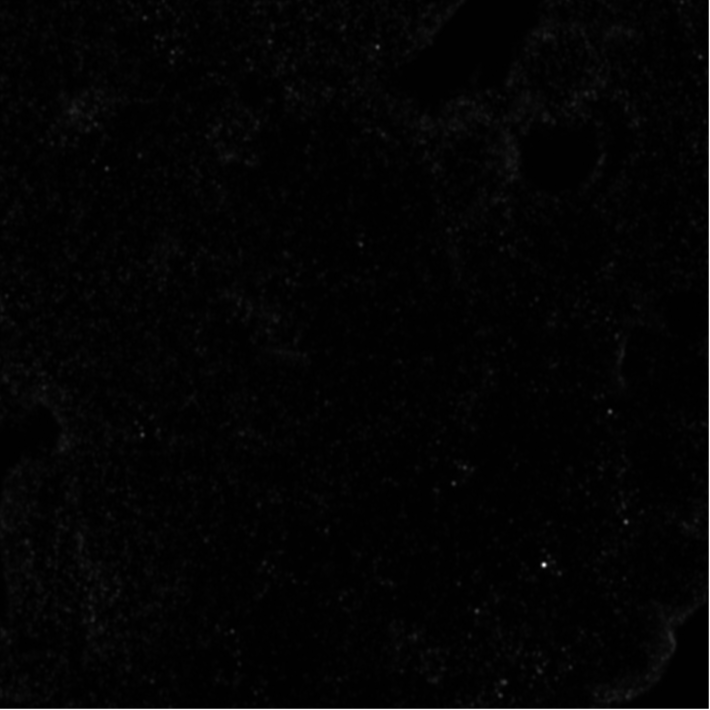

Supplement: Supplementary file 5 — Source data Fig. 2 [file 44319_2025_511_MOESM5_ESM.zip › Figure 2/Fig 2L Images/rnpc3 WT tp53 WT H2AX gH2AX.tif]

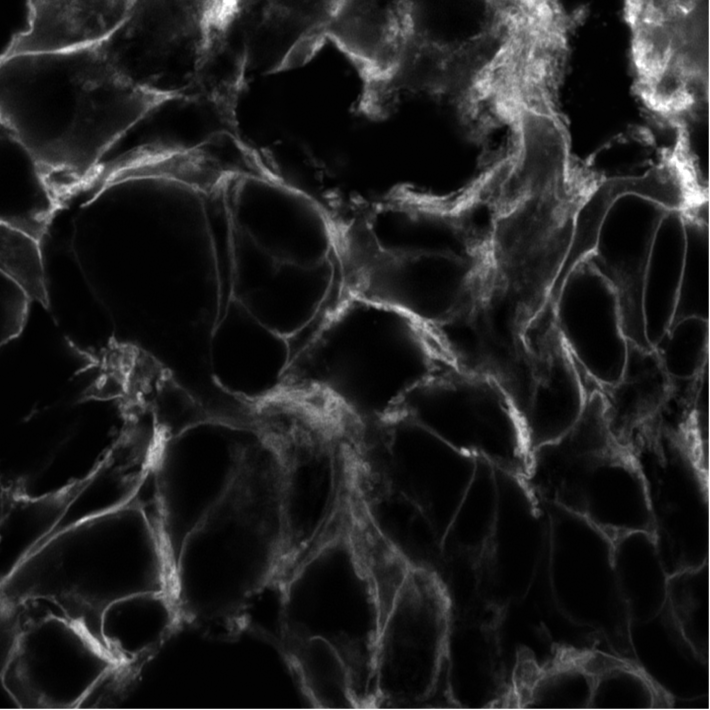

Supplement: Supplementary file 5 — Source data Fig. 2 [file 44319_2025_511_MOESM5_ESM.zip › Figure 2/Fig 2L Images/rnpc3 WT tp53 MUT H2AX GFP.tif]

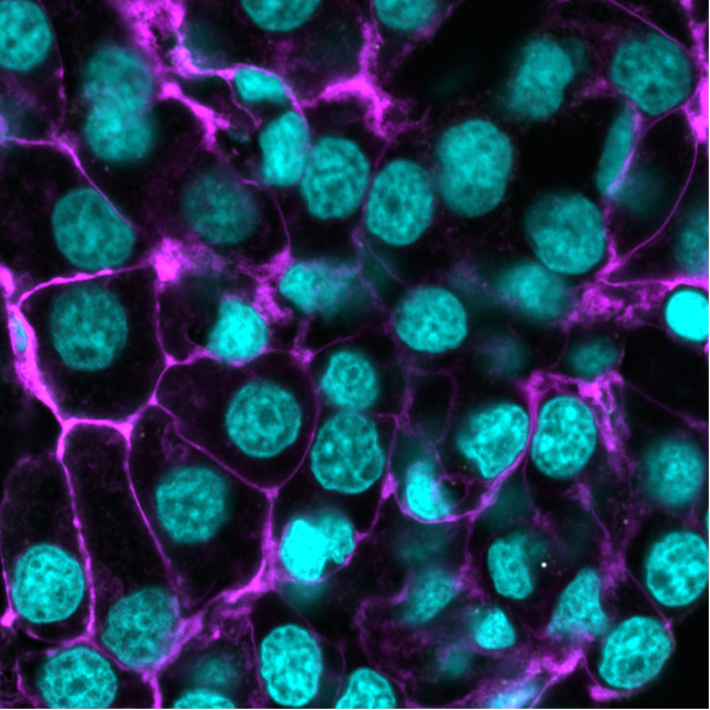

Supplement: Supplementary file 5 — Source data Fig. 2 [file 44319_2025_511_MOESM5_ESM.zip › Figure 2/Fig 2L Images/rnpc3 WT tp53 WT H2AX merge.tif]

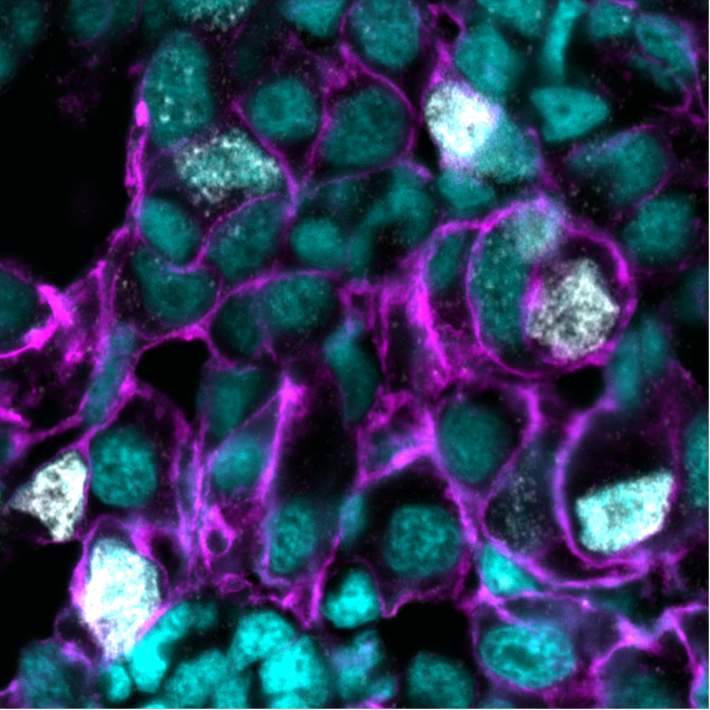

Supplement: Supplementary file 5 — Source data Fig. 2 [file 44319_2025_511_MOESM5_ESM.zip › Figure 2/Fig 2L Images/rnpc3 HET tp53 MUT H2AX merge.tif]

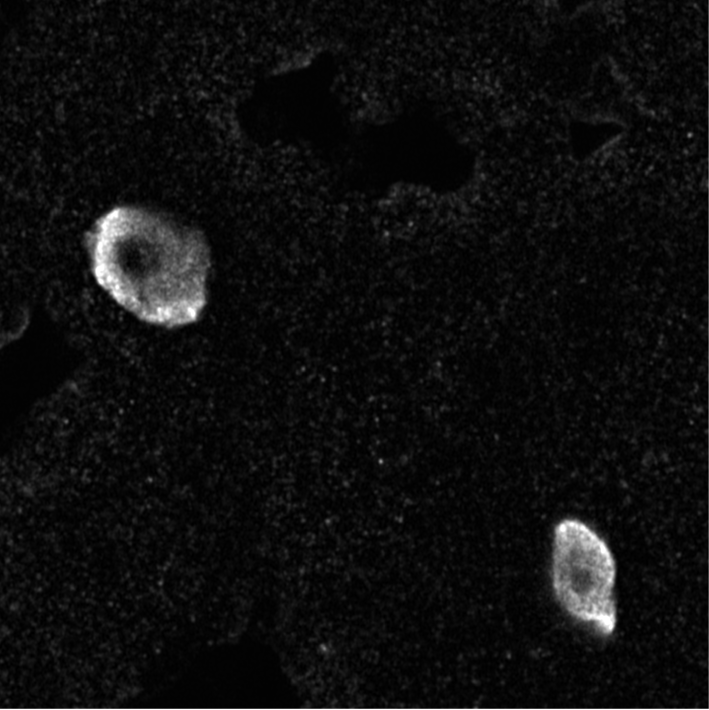

Supplement: Supplementary file 5 — Source data Fig. 2 [file 44319_2025_511_MOESM5_ESM.zip › Figure 2/Fig 2L Images/rnpc3 WT tp53 MUT H2AX gH2AX.tif]

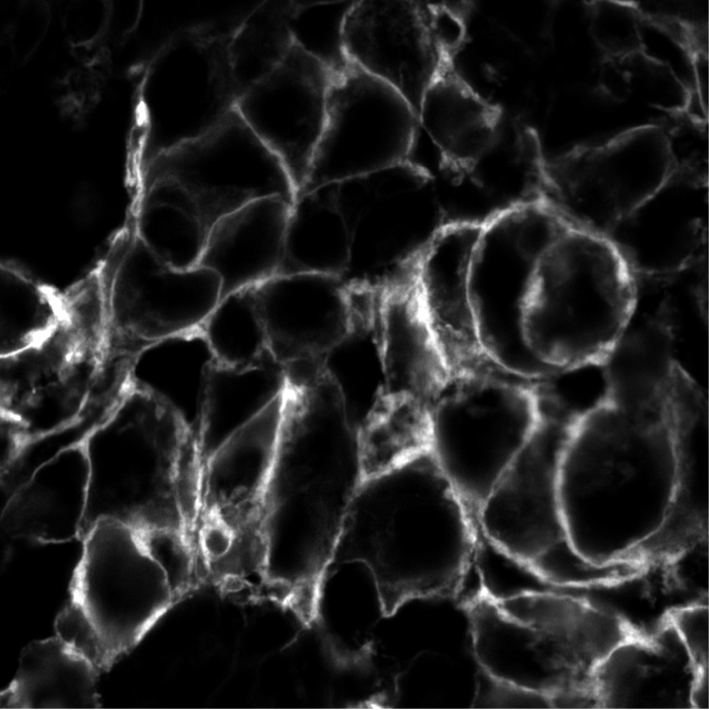

Supplement: Supplementary file 5 — Source data Fig. 2 [file 44319_2025_511_MOESM5_ESM.zip › Figure 2/Fig 2L Images/rnpc3 HET tp53 MUT H2AX GFP.tif]

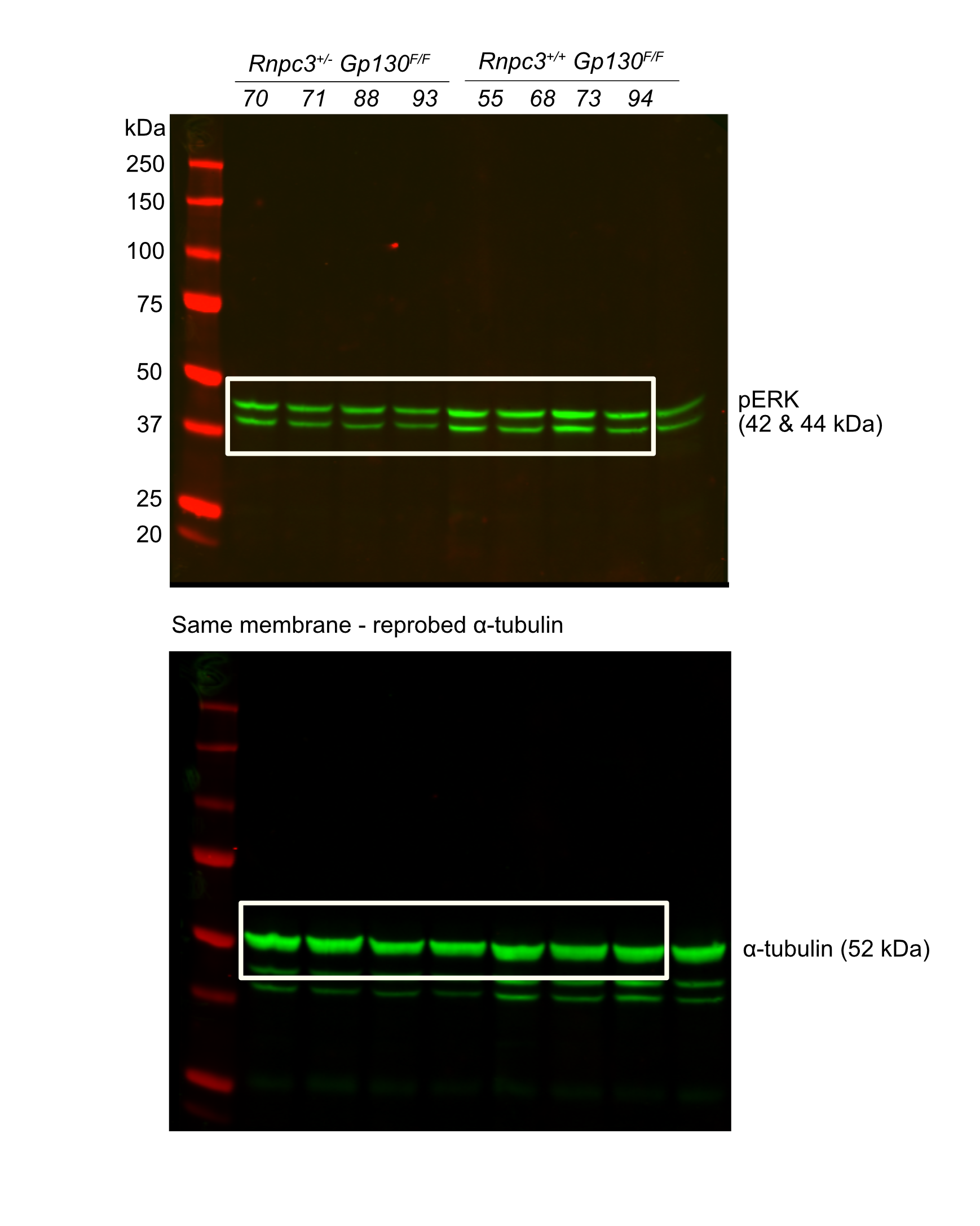

Supplement: Supplementary file 6 — Source data Fig. 3 [file 44319_2025_511_MOESM6_ESM.zip › Figure 3/Fig 3J Western.tiff]

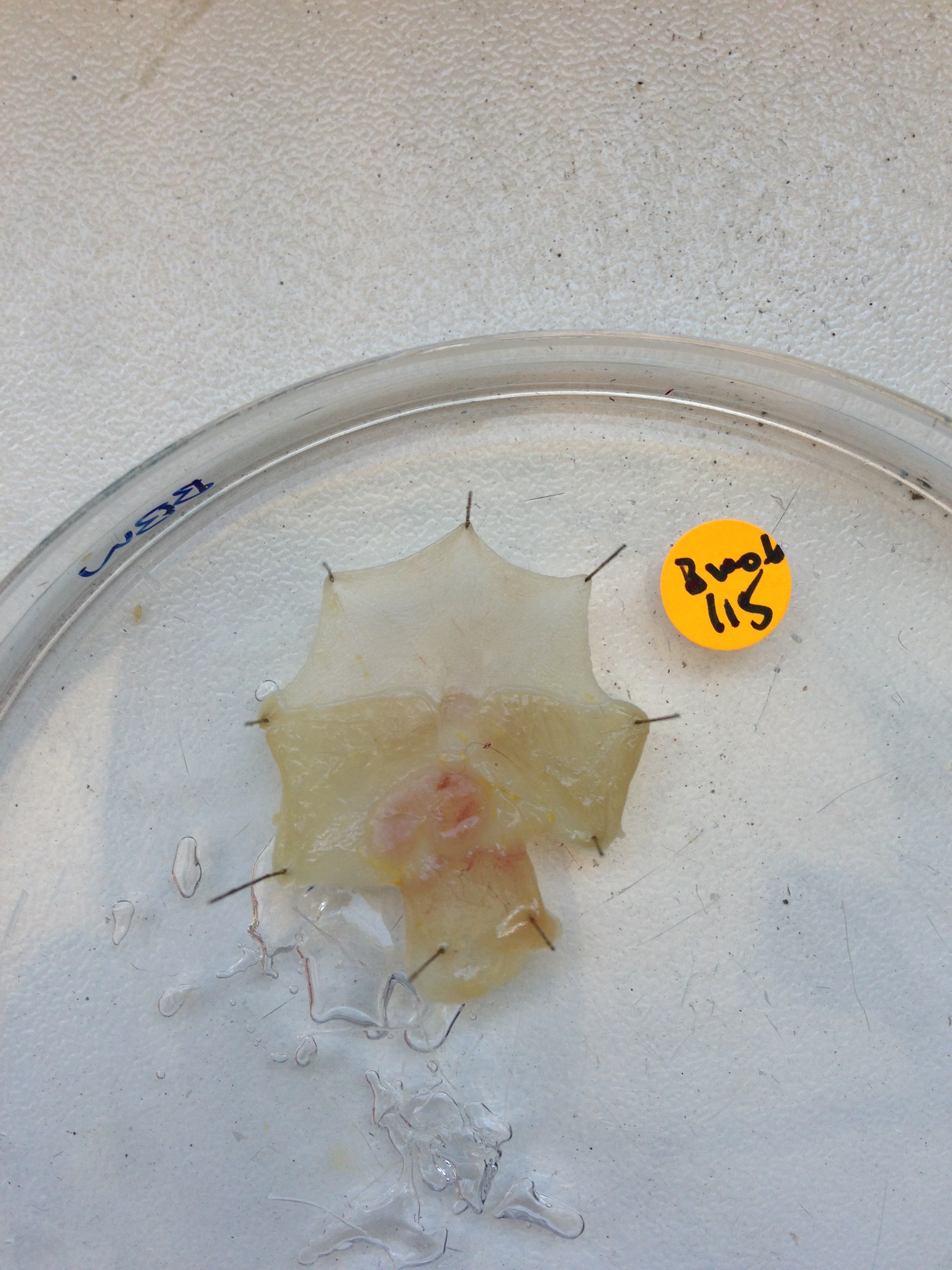

Supplement: Supplementary file 6 — Source data Fig. 3 [file 44319_2025_511_MOESM6_ESM.zip › Figure 3/Fig 3E Images/100day RNPC3 HET.JPG]

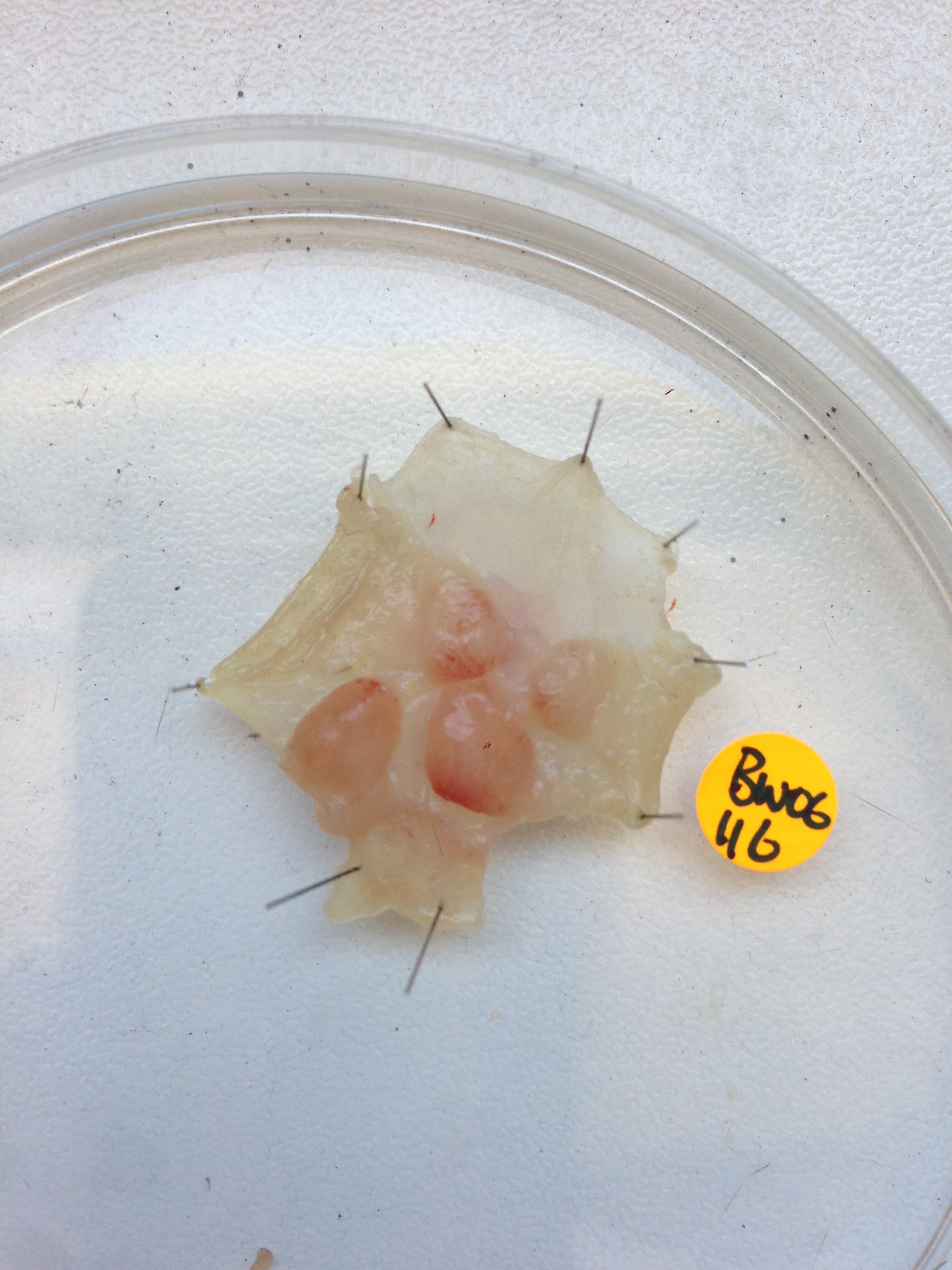

Supplement: Supplementary file 6 — Source data Fig. 3 [file 44319_2025_511_MOESM6_ESM.zip › Figure 3/Fig 3E Images/100day WT.JPG]

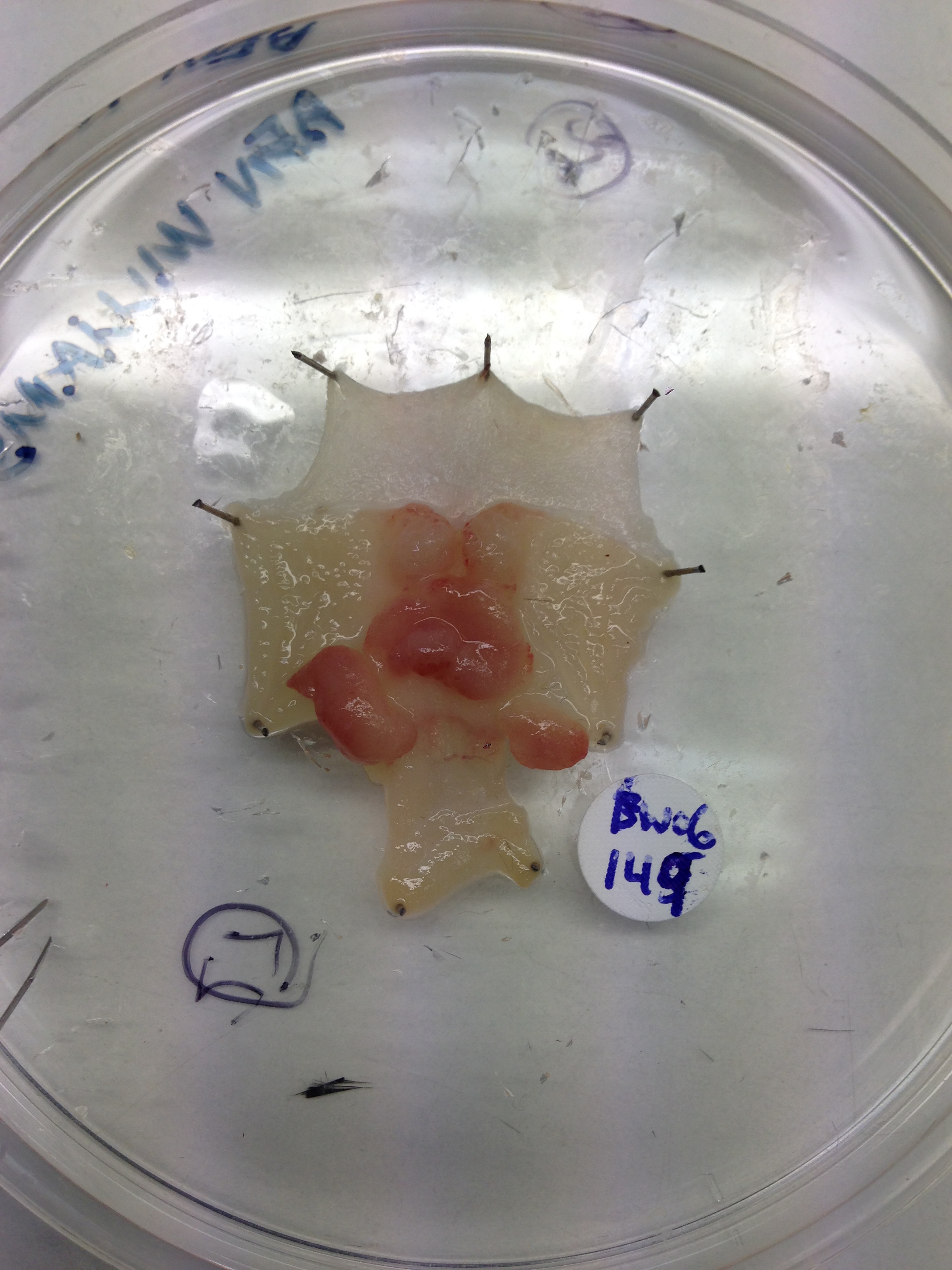

Supplement: Supplementary file 6 — Source data Fig. 3 [file 44319_2025_511_MOESM6_ESM.zip › Figure 3/Fig 3E Images/180day WT.JPG]

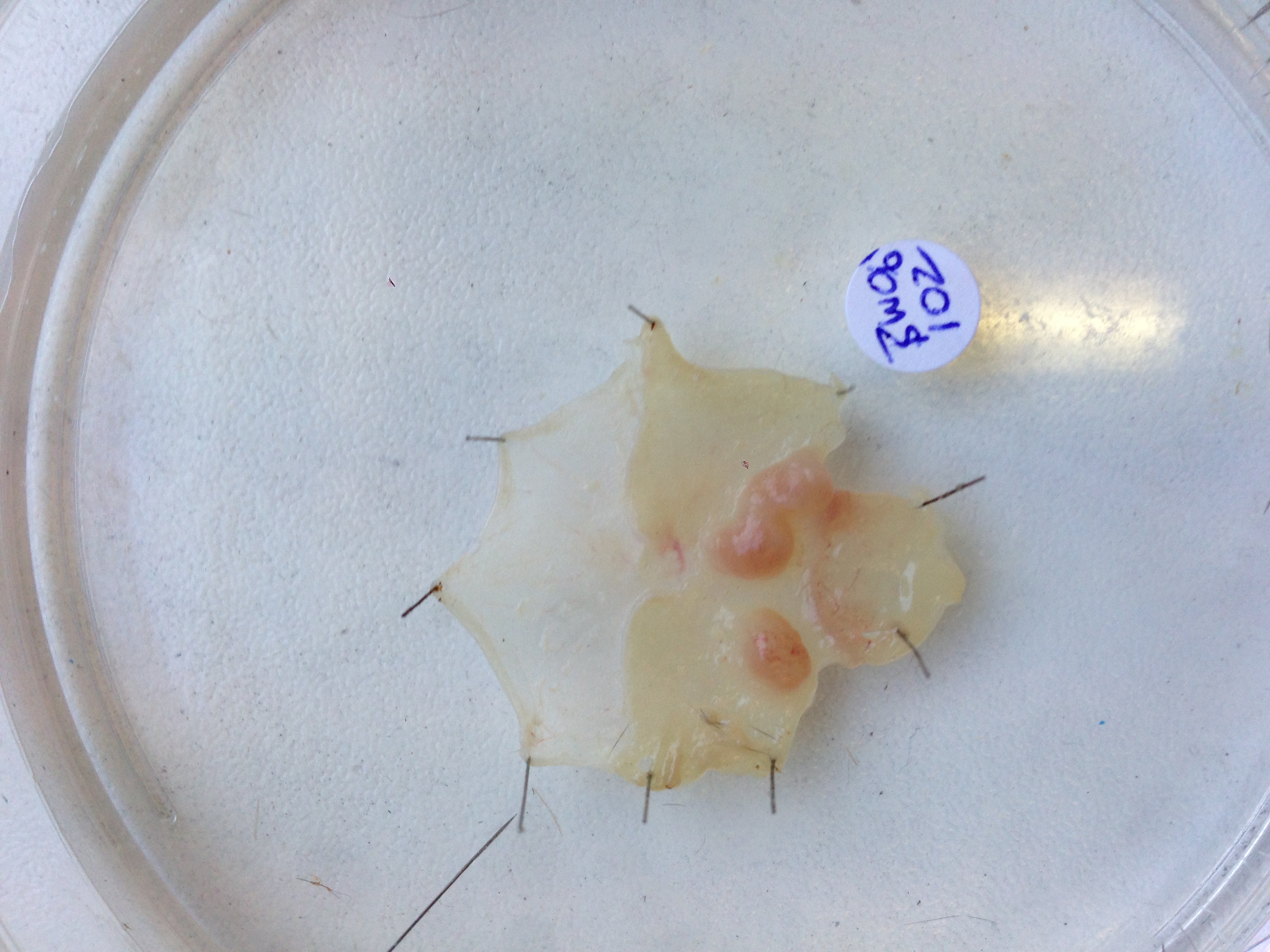

Supplement: Supplementary file 6 — Source data Fig. 3 [file 44319_2025_511_MOESM6_ESM.zip › Figure 3/Fig 3E Images/180day RNPC3 HET.JPG]

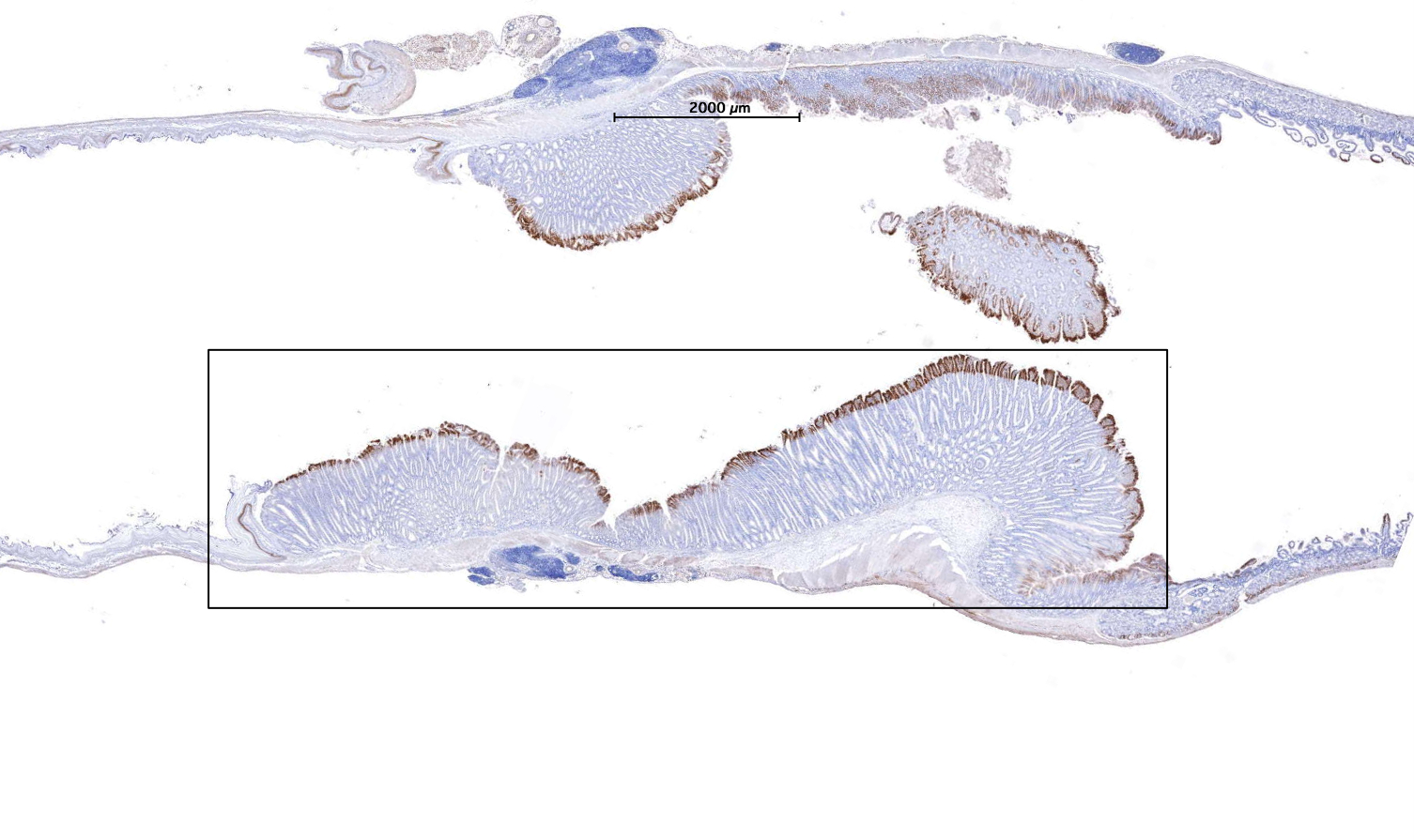

Supplement: Supplementary file 6 — Source data Fig. 3 [file 44319_2025_511_MOESM6_ESM.zip › Figure 3/Fig 3I Images/WT_pERK_1x.tiff]

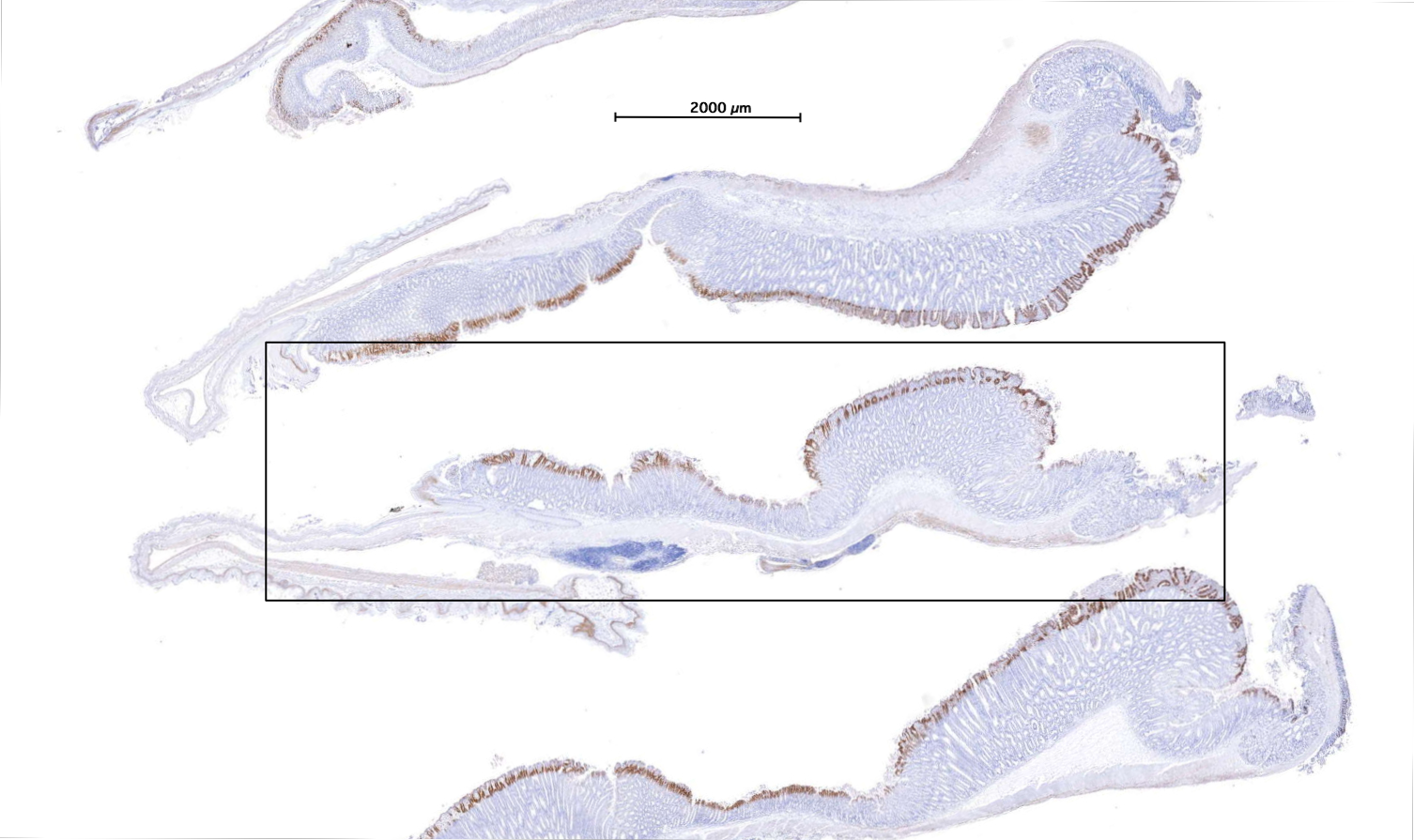

Supplement: Supplementary file 6 — Source data Fig. 3 [file 44319_2025_511_MOESM6_ESM.zip › Figure 3/Fig 3I Images/RNPC3HET_pERK_1x.tiff]

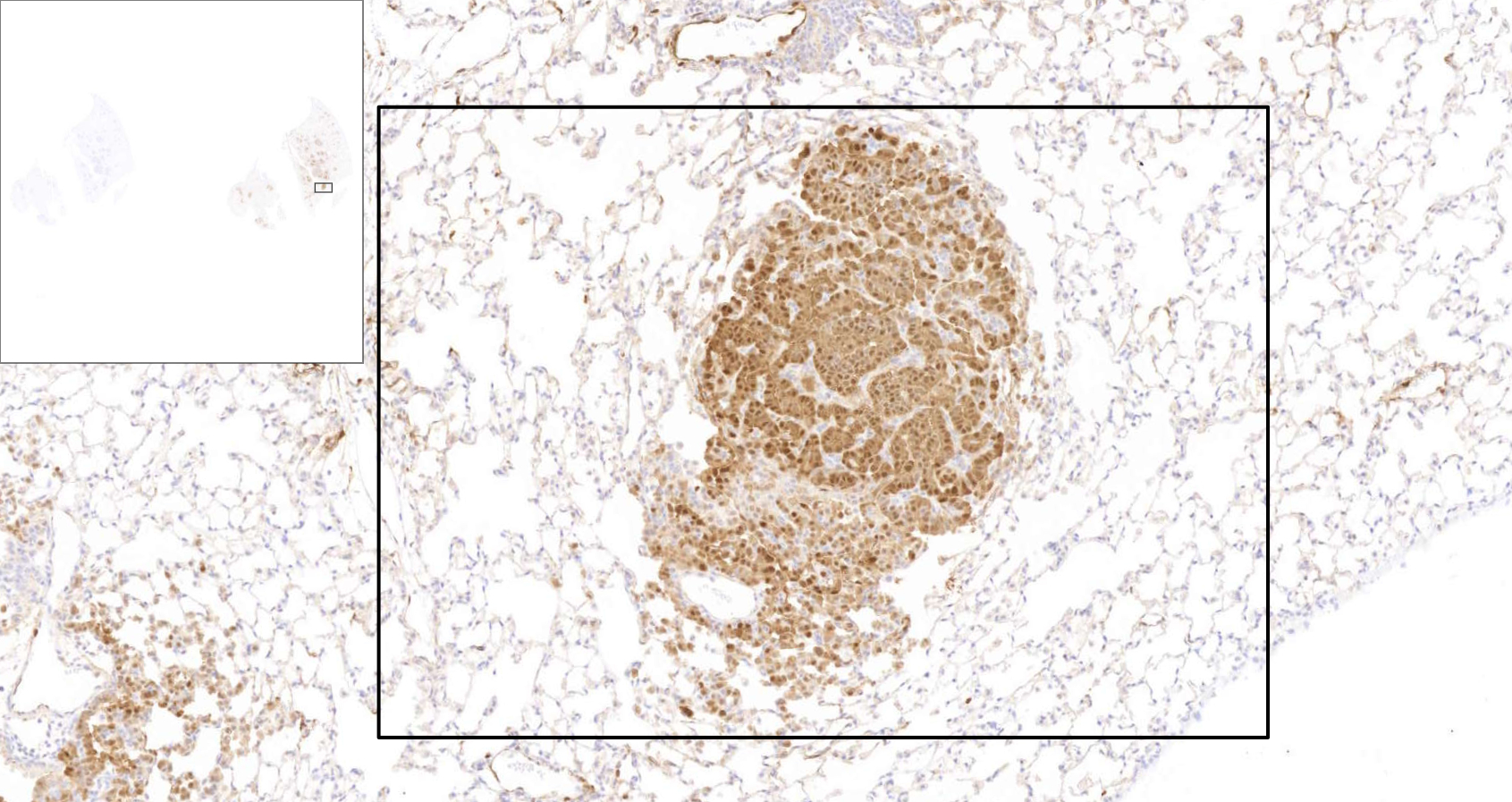

Supplement: Supplementary file 6 — Source data Fig. 3 [file 44319_2025_511_MOESM6_ESM.zip › Figure 3/Fig 3C Images/antipERK_HET_10x.tiff]

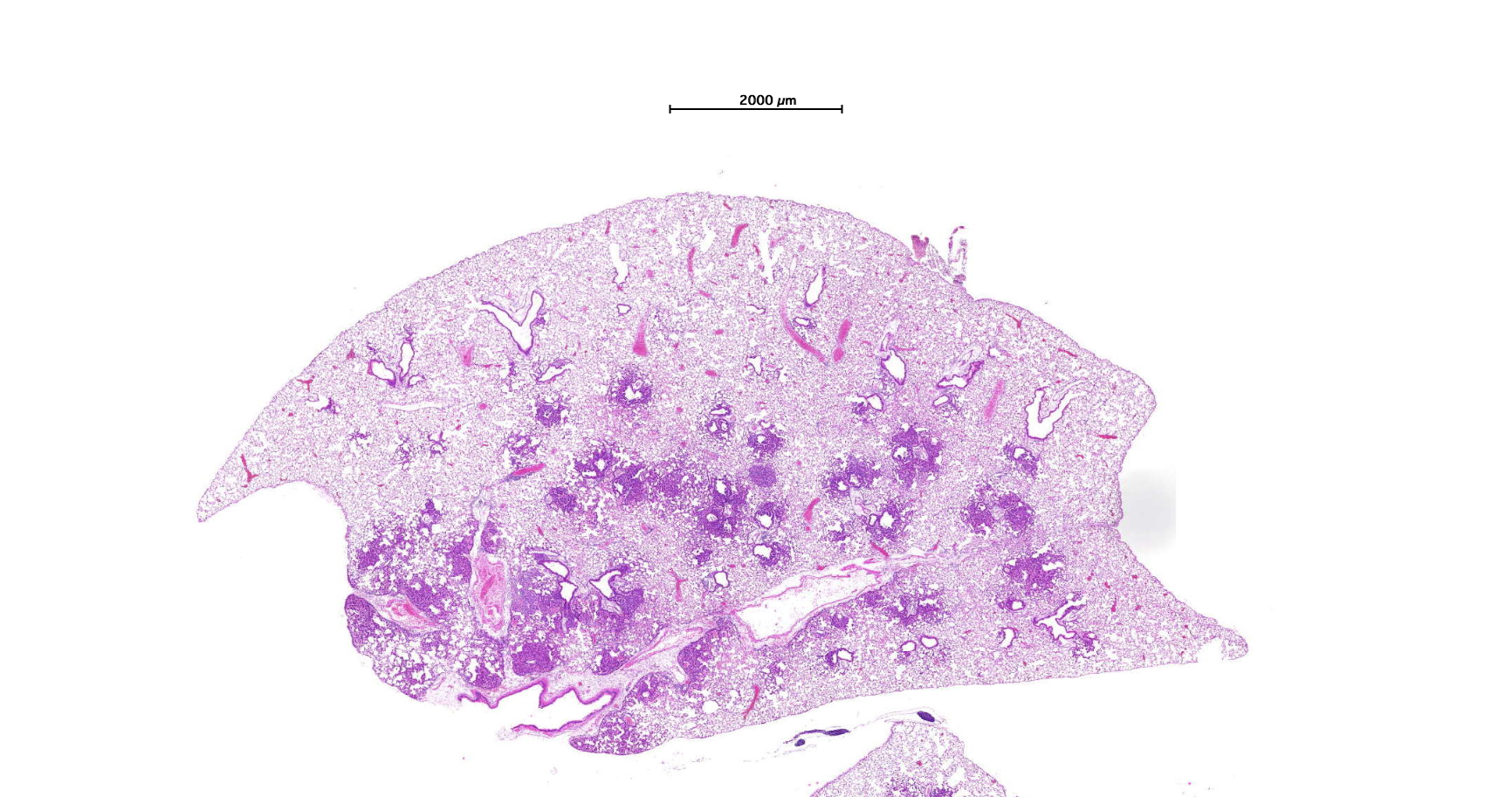

Supplement: Supplementary file 6 — Source data Fig. 3 [file 44319_2025_511_MOESM6_ESM.zip › Figure 3/Fig 3C Images/rnpc3HET_1x.jpg]

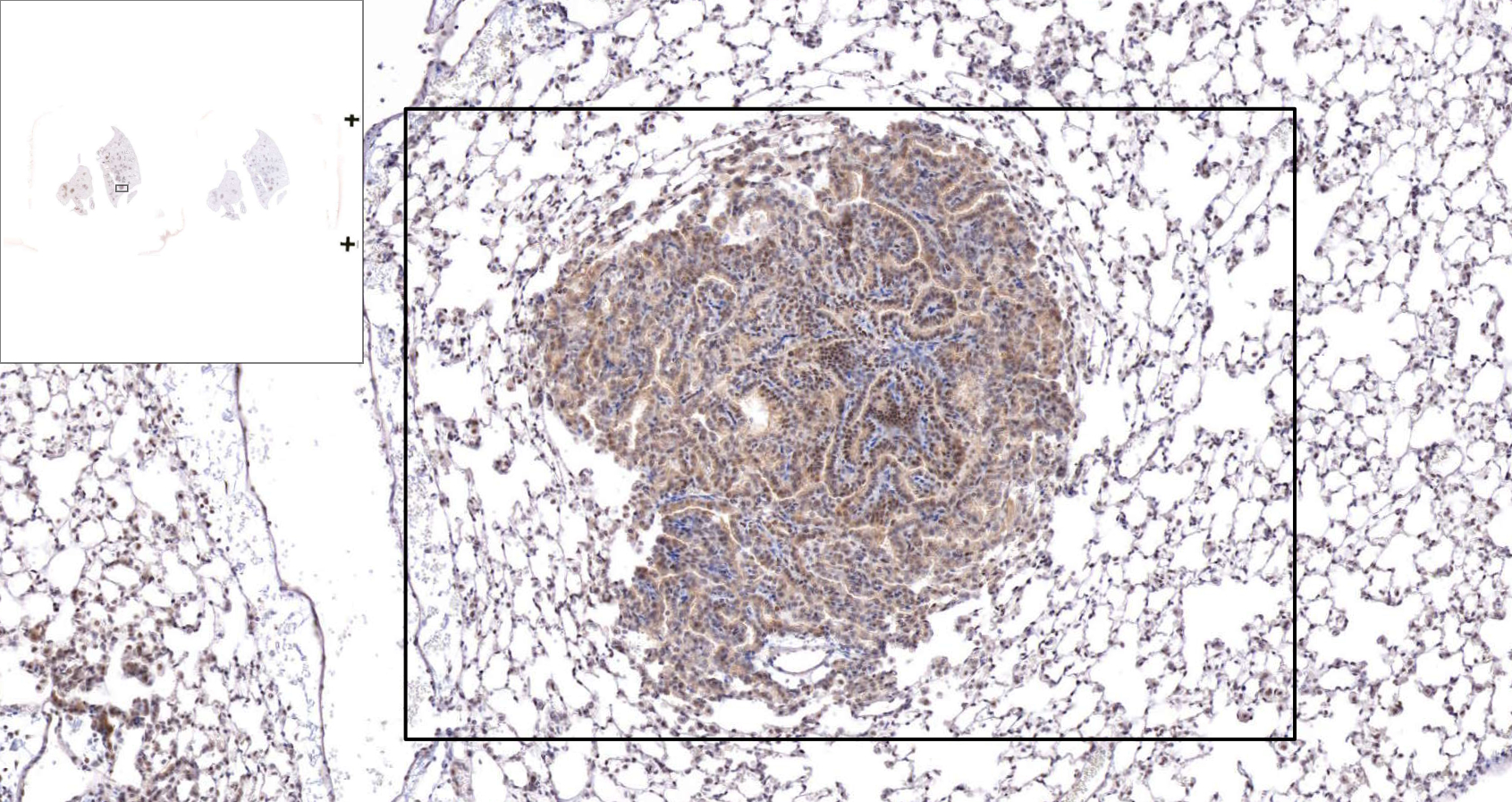

Supplement: Supplementary file 6 — Source data Fig. 3 [file 44319_2025_511_MOESM6_ESM.zip › Figure 3/Fig 3C Images/anti65K_HET_10x.tiff]

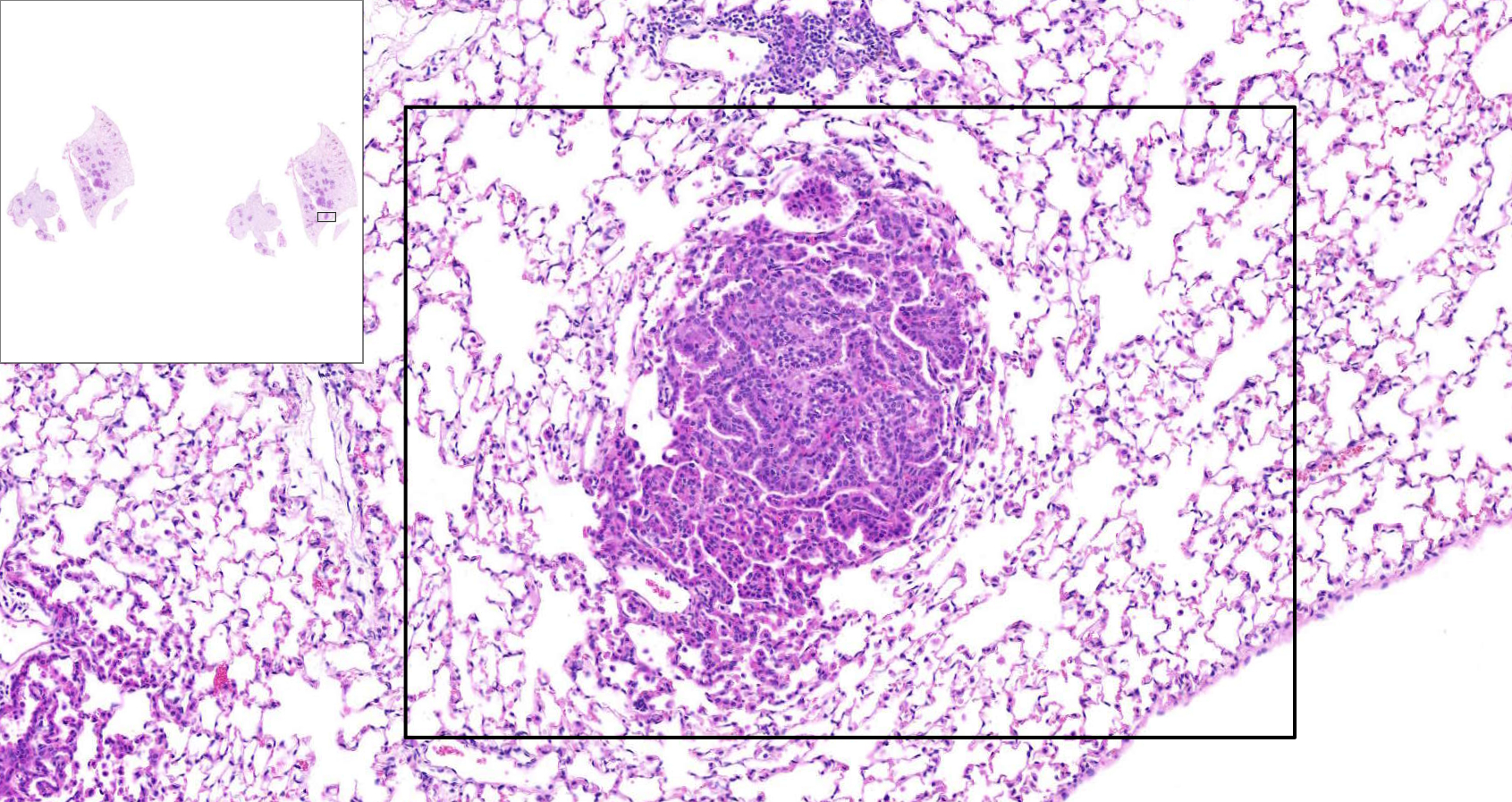

Supplement: Supplementary file 6 — Source data Fig. 3 [file 44319_2025_511_MOESM6_ESM.zip › Figure 3/Fig 3C Images/HE_HET_10x.tiff]

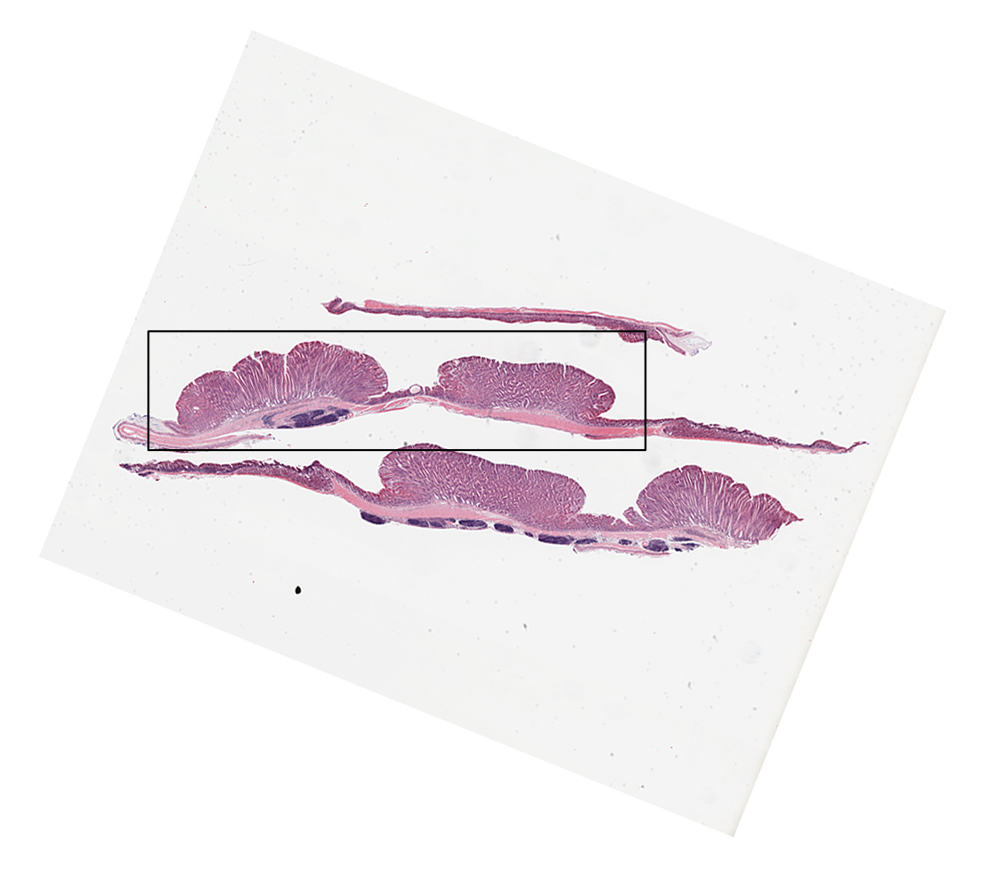

Supplement: Supplementary file 6 — Source data Fig. 3 [file 44319_2025_511_MOESM6_ESM.zip › Figure 3/Fig 3H Images/100day_WT_HE.tiff]

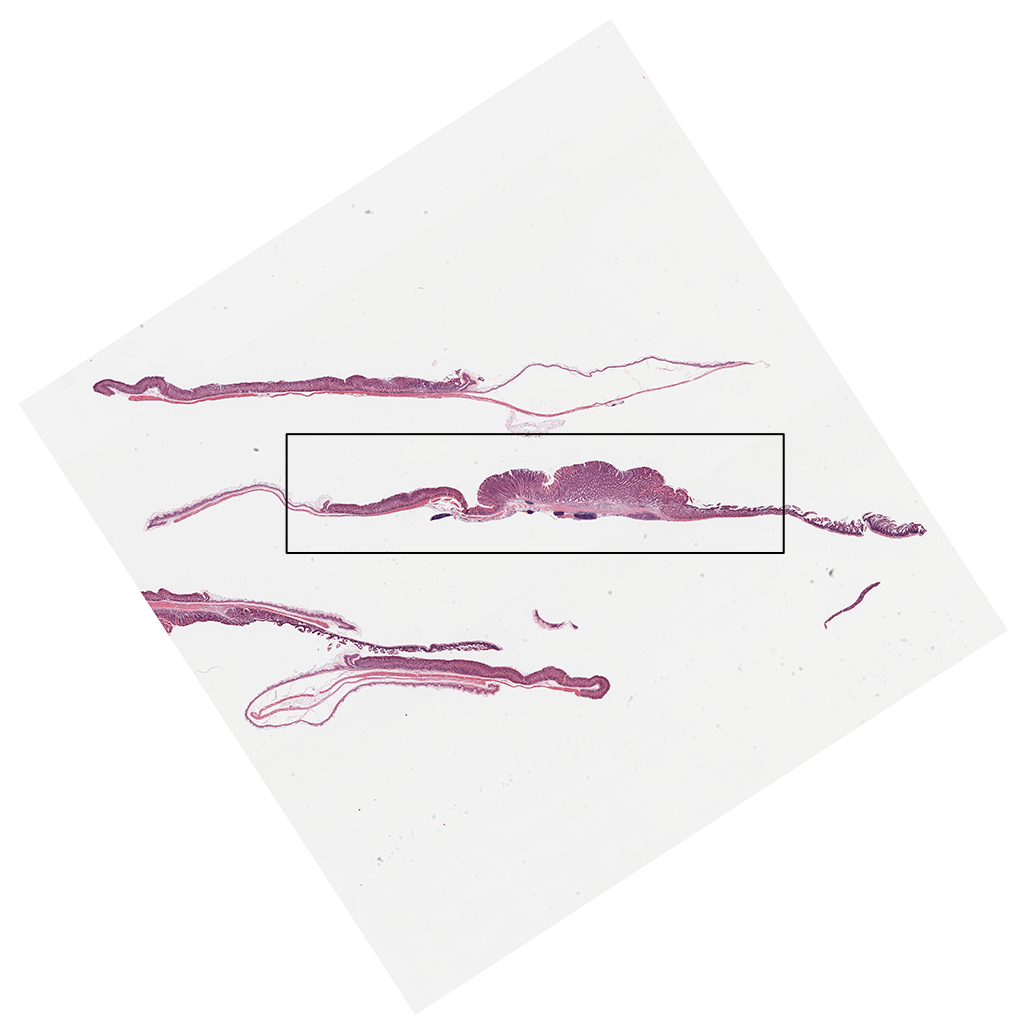

Supplement: Supplementary file 6 — Source data Fig. 3 [file 44319_2025_511_MOESM6_ESM.zip › Figure 3/Fig 3H Images/100day_RNPC3HET_HE.tiff]

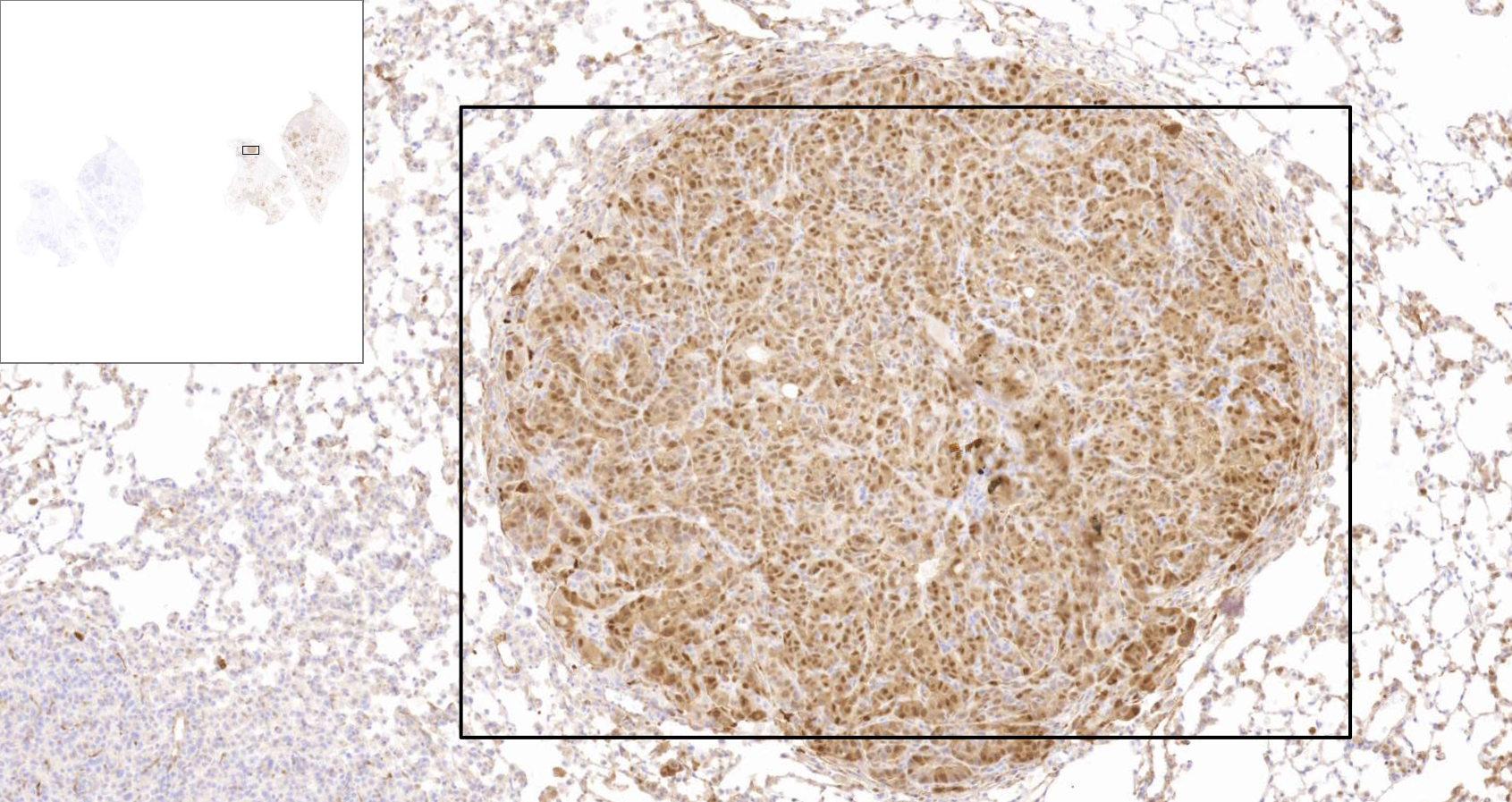

Supplement: Supplementary file 6 — Source data Fig. 3 [file 44319_2025_511_MOESM6_ESM.zip › Figure 3/Fig 3B Images/pERK_WT_10x.tiff]

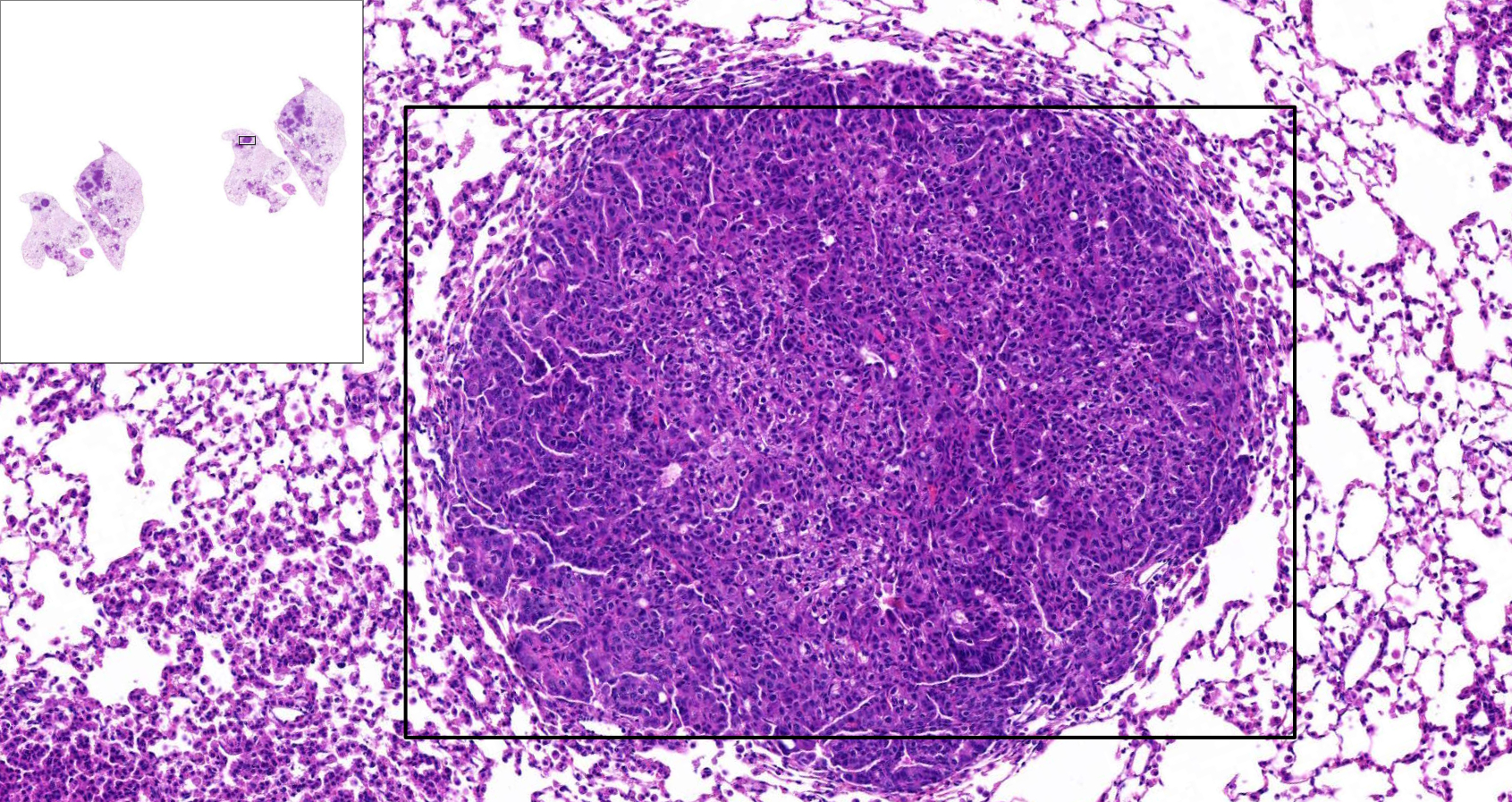

Supplement: Supplementary file 6 — Source data Fig. 3 [file 44319_2025_511_MOESM6_ESM.zip › Figure 3/Fig 3B Images/HE_WT_10x.tiff]

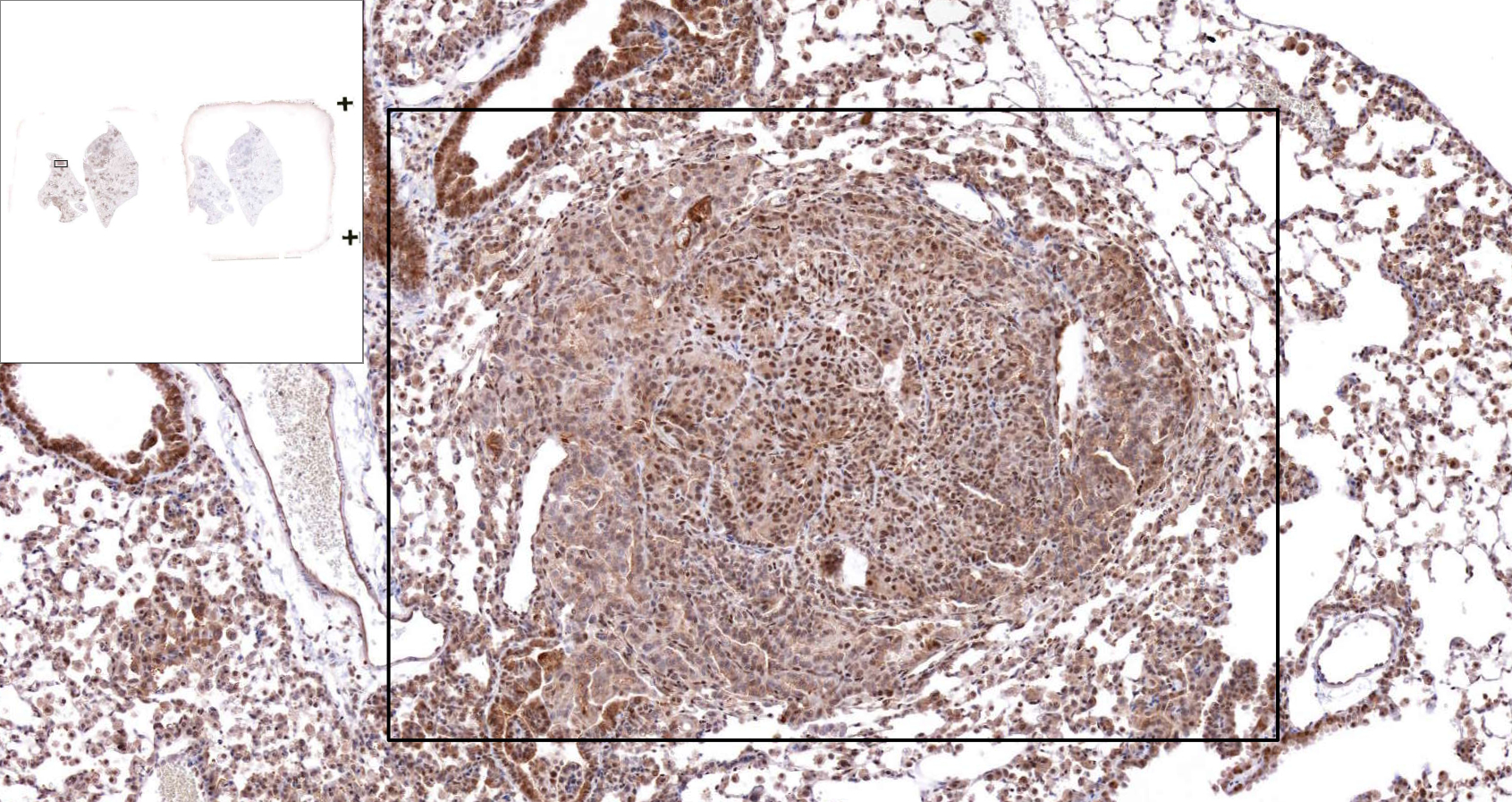

Supplement: Supplementary file 6 — Source data Fig. 3 [file 44319_2025_511_MOESM6_ESM.zip › Figure 3/Fig 3B Images/anti65K_WT_10x.tiff]

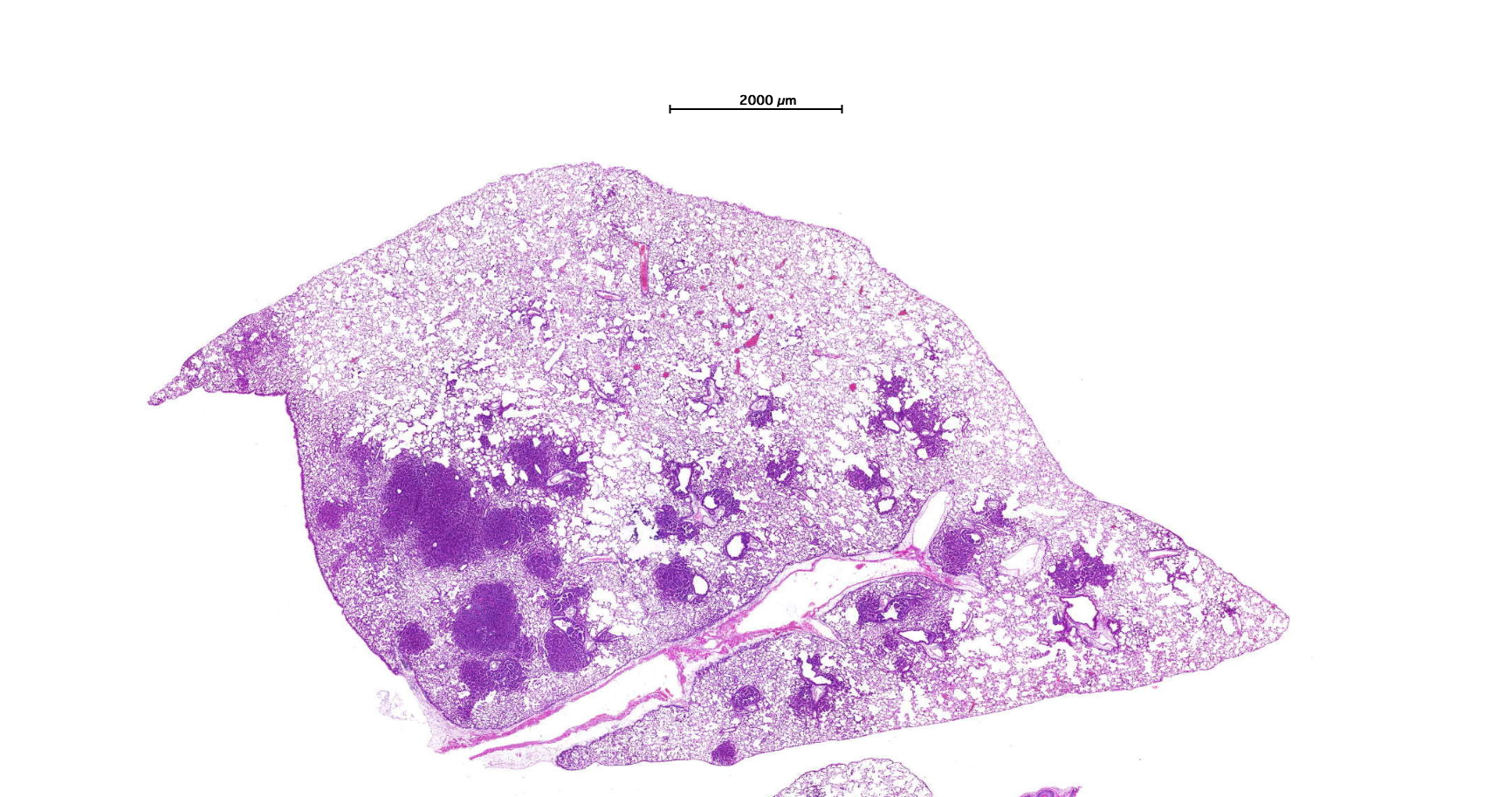

Supplement: Supplementary file 6 — Source data Fig. 3 [file 44319_2025_511_MOESM6_ESM.zip › Figure 3/Fig 3B Images/rnpc3WT_1x.jpg]

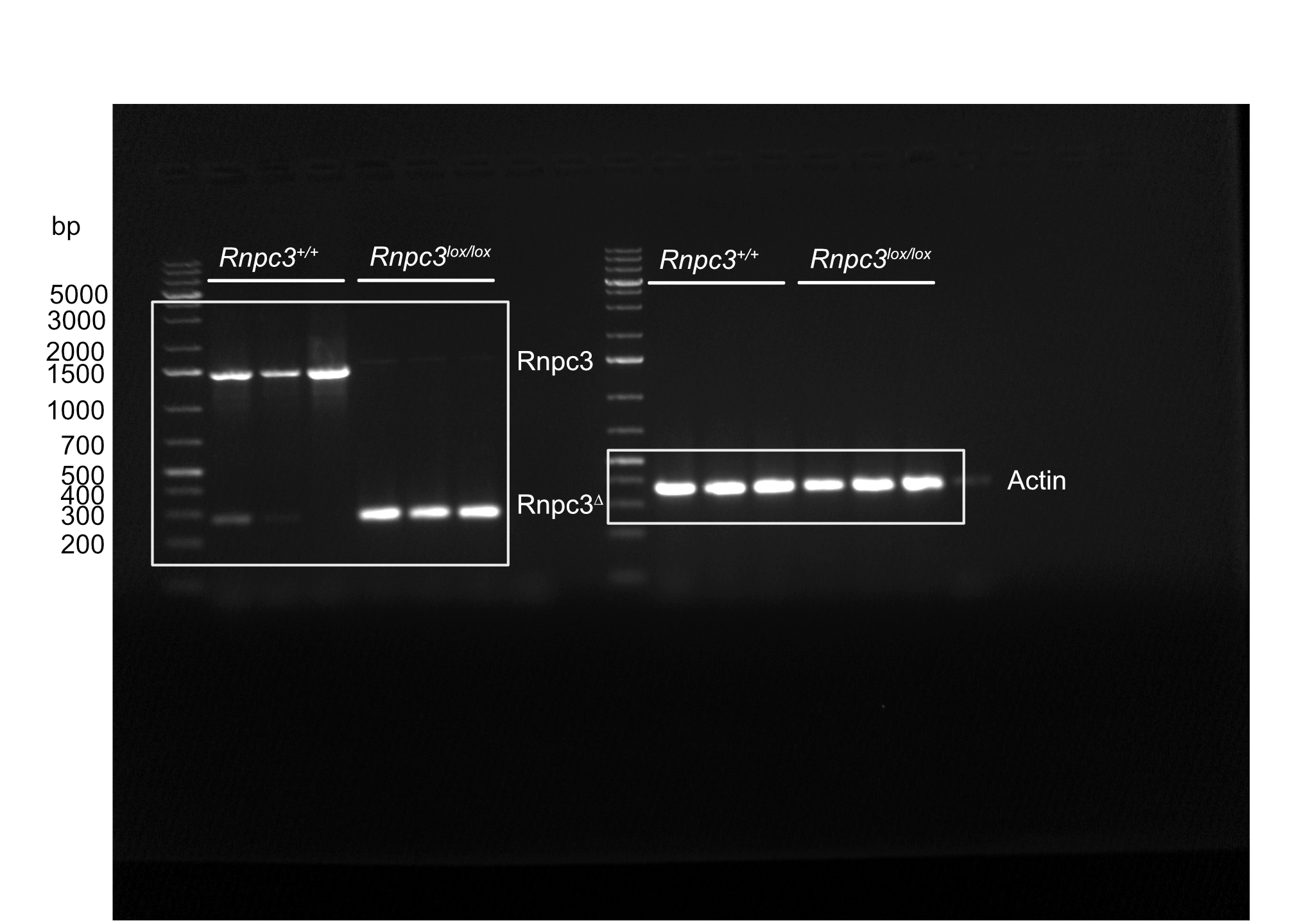

Supplement: Supplementary file 7 — Source data Fig. 4 [file 44319_2025_511_MOESM7_ESM.zip › Figure 4/Fig 4B Gel image.tiff]

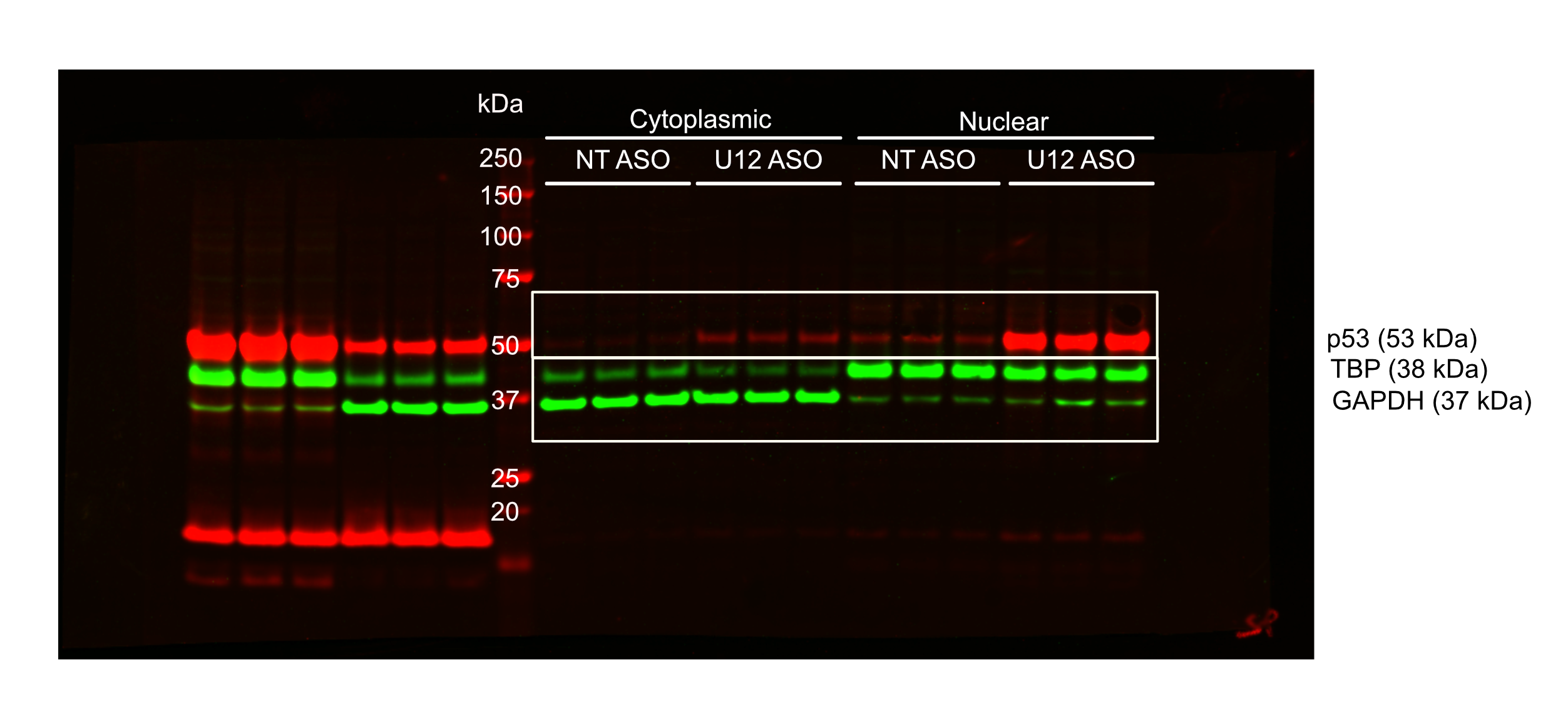

Supplement: Supplementary file 9 — Source data Fig. 6 [file 44319_2025_511_MOESM9_ESM.zip › Figure 6/Fig 6E Western.tiff]

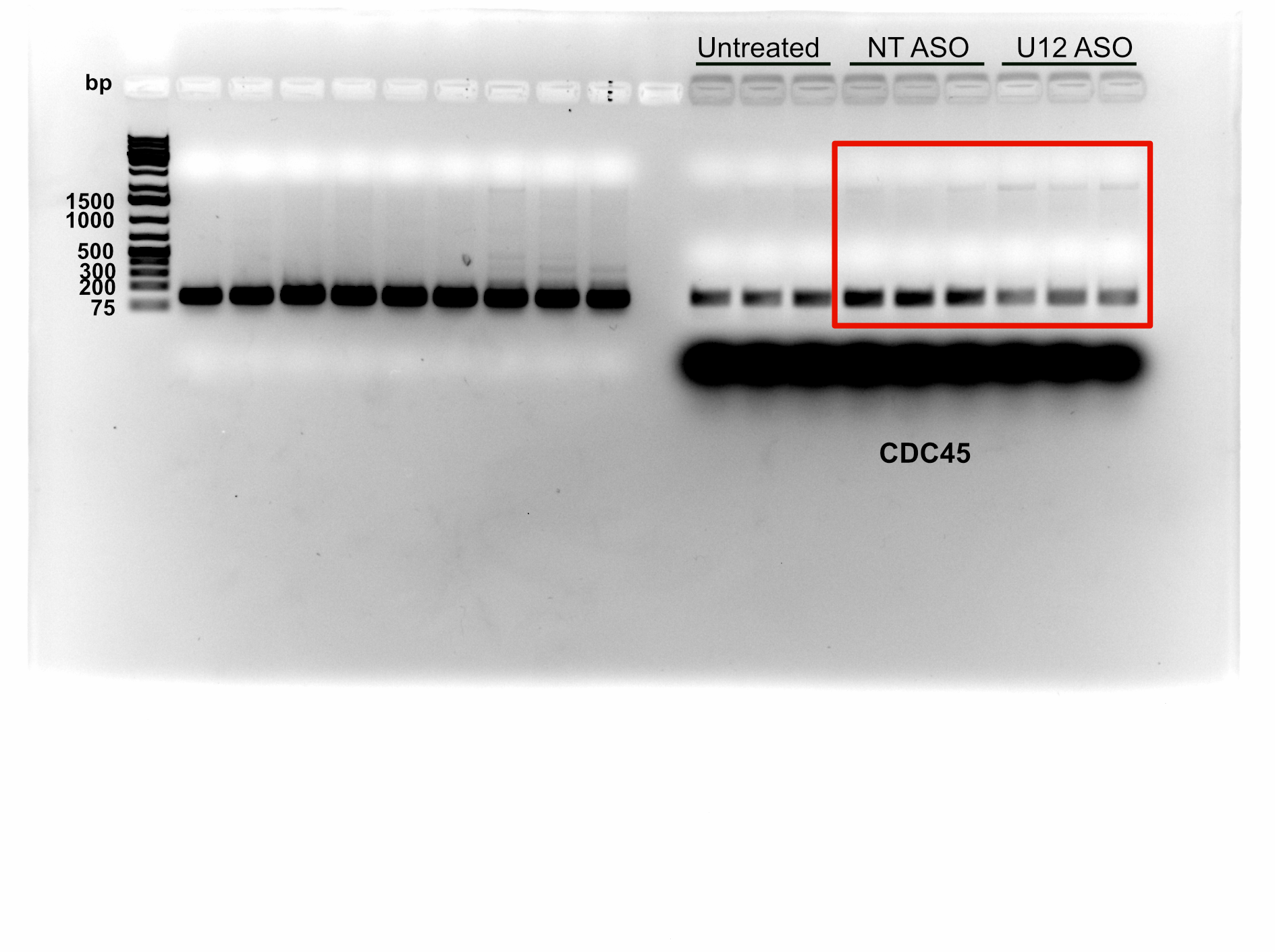

Supplement: Supplementary file 9 — Source data Fig. 6 [file 44319_2025_511_MOESM9_ESM.zip › Figure 6/Fig 6A Gel images/CDC45.tiff]

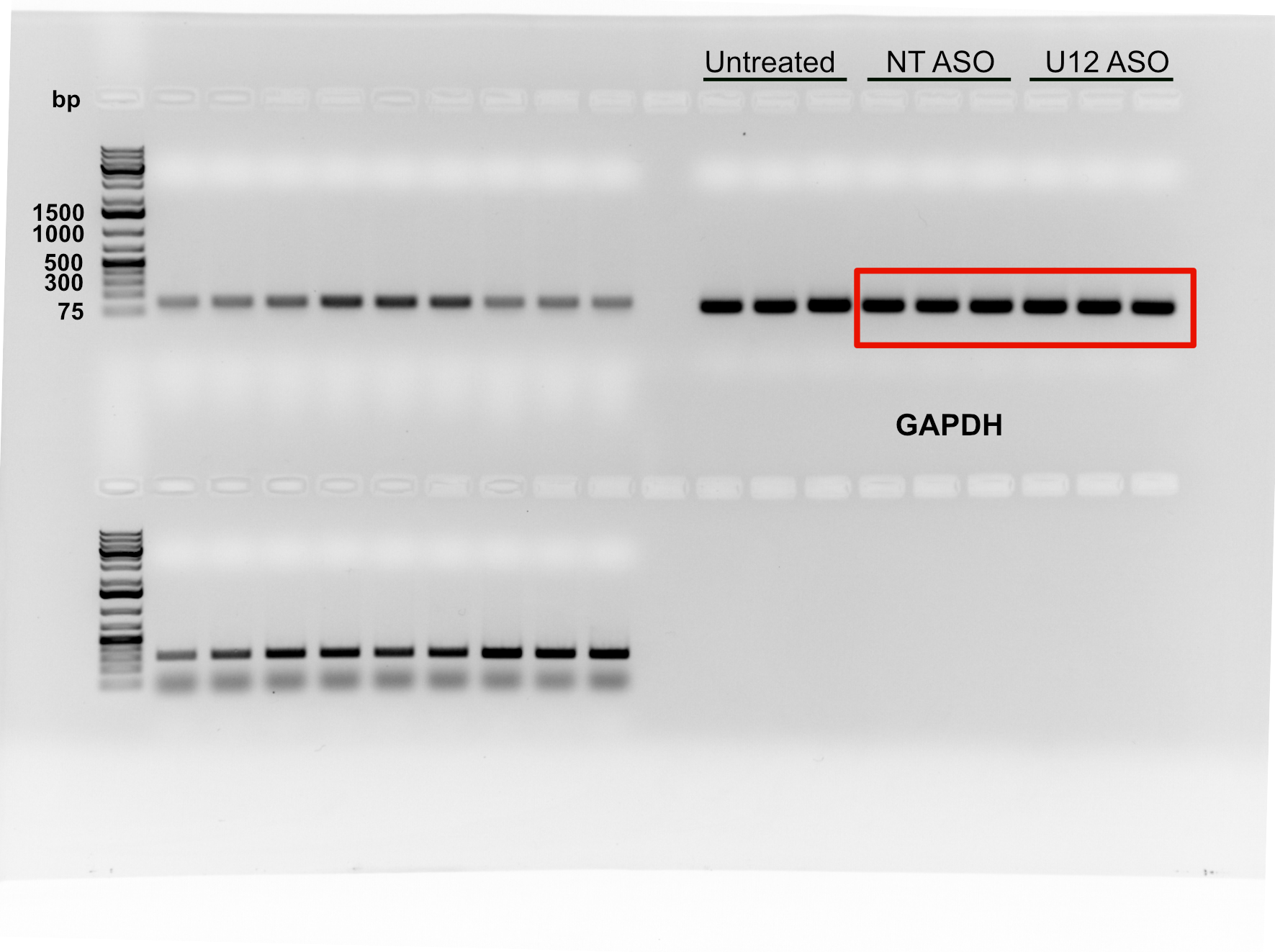

Supplement: Supplementary file 9 — Source data Fig. 6 [file 44319_2025_511_MOESM9_ESM.zip › Figure 6/Fig 6A Gel images/GAPDH.tiff]

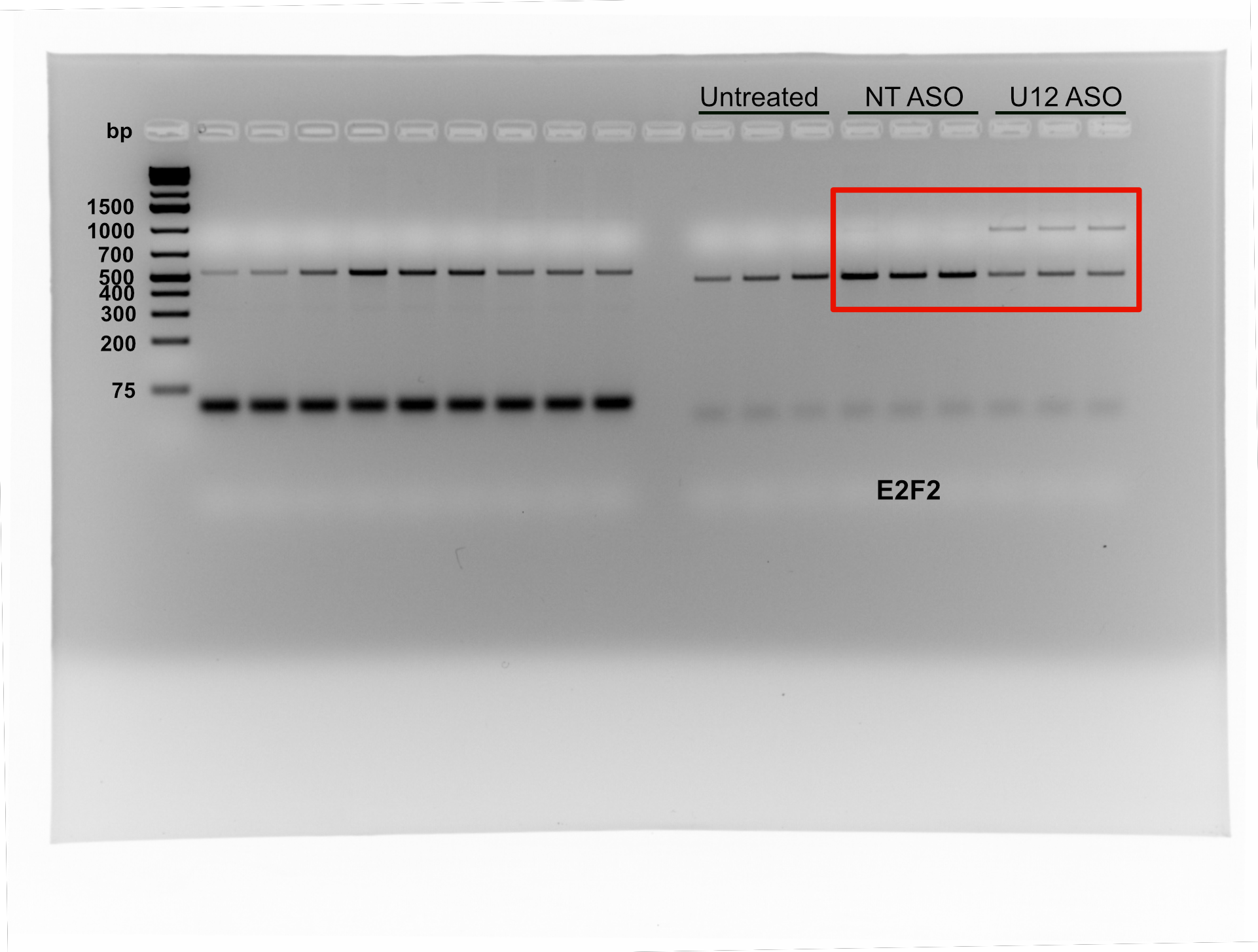

Supplement: Supplementary file 9 — Source data Fig. 6 [file 44319_2025_511_MOESM9_ESM.zip › Figure 6/Fig 6A Gel images/E2F2.tiff]

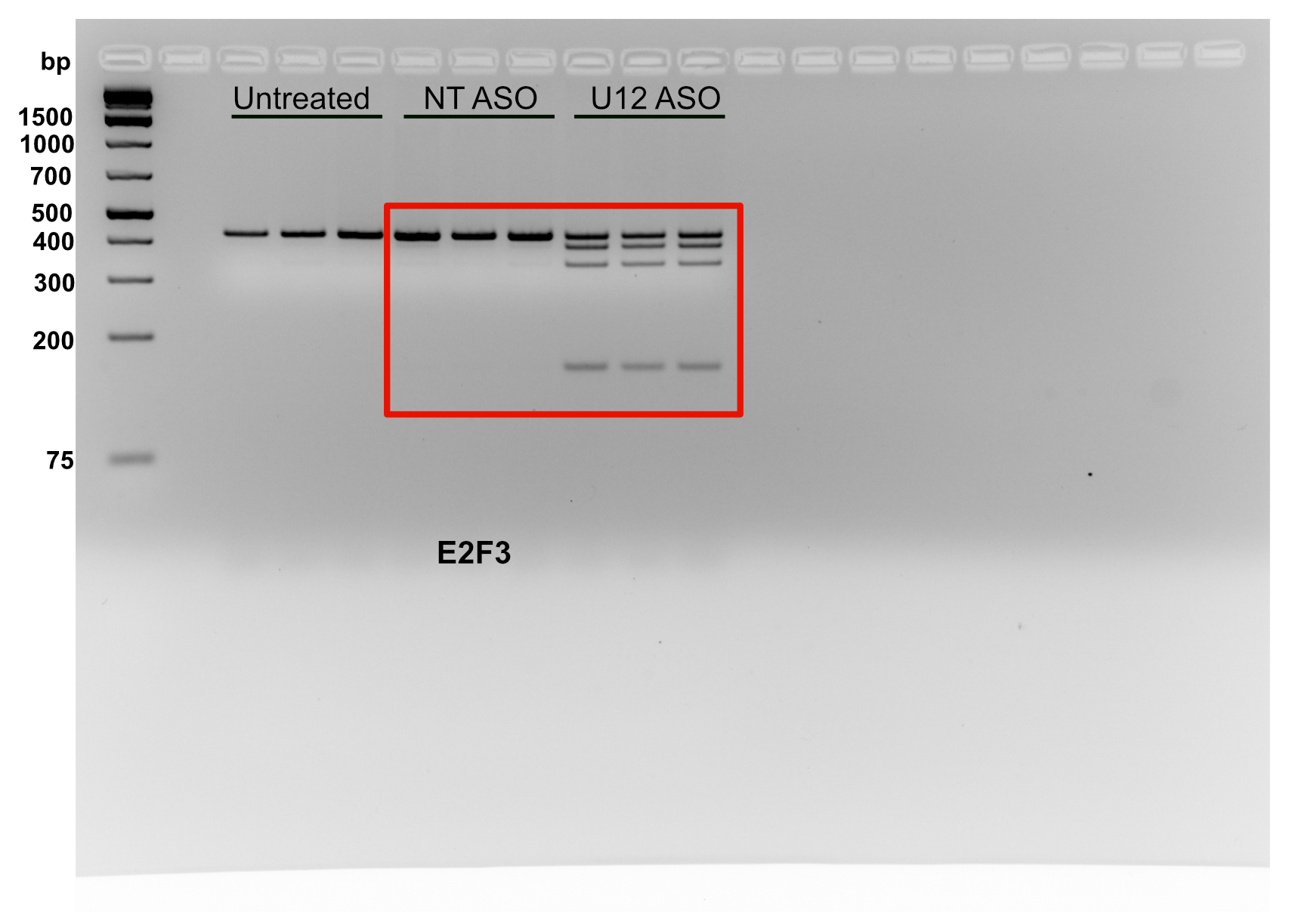

Supplement: Supplementary file 9 — Source data Fig. 6 [file 44319_2025_511_MOESM9_ESM.zip › Figure 6/Fig 6A Gel images/E2F3.tiff]

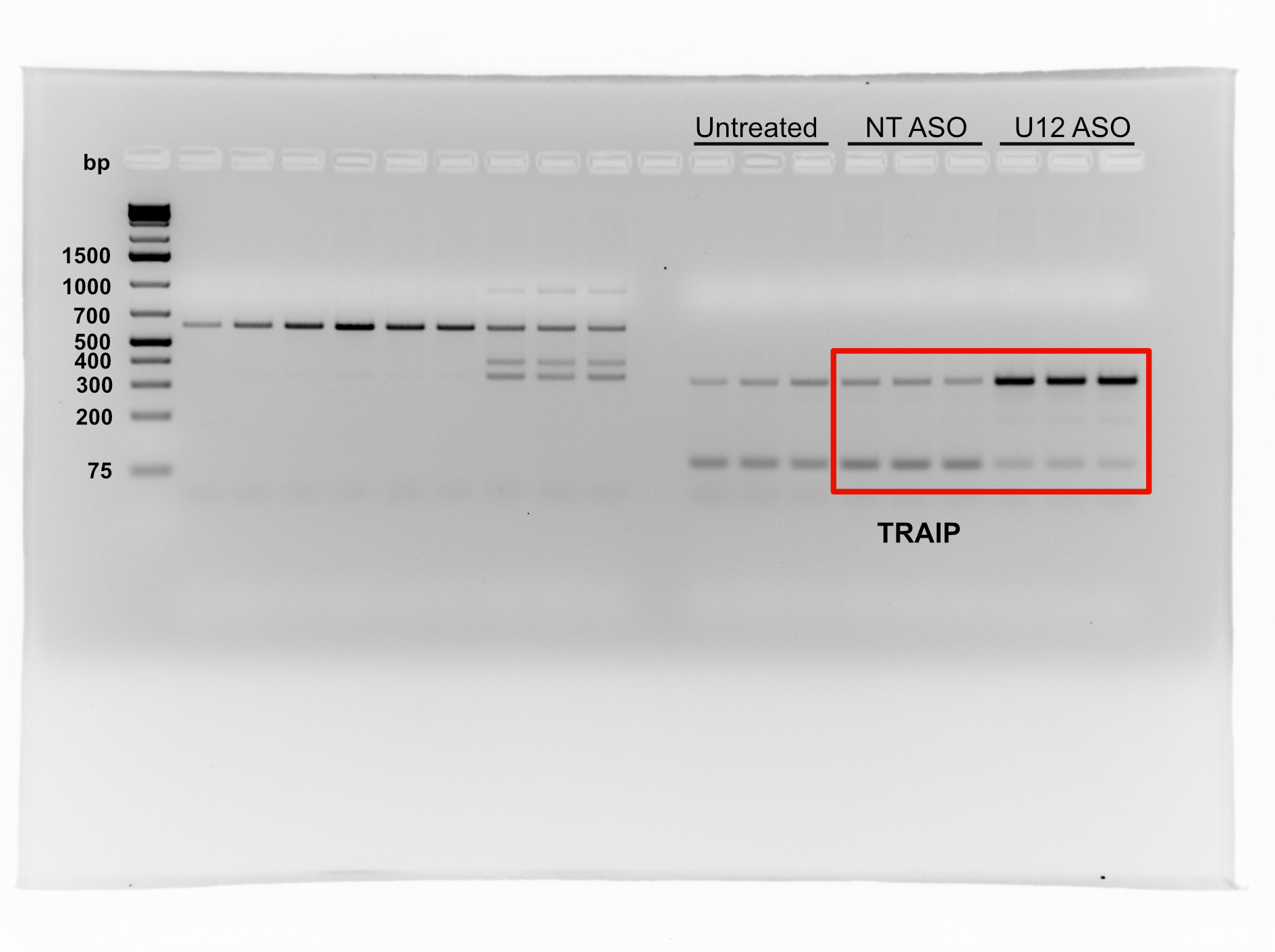

Supplement: Supplementary file 9 — Source data Fig. 6 [file 44319_2025_511_MOESM9_ESM.zip › Figure 6/Fig 6A Gel images/TRAIP.tiff]

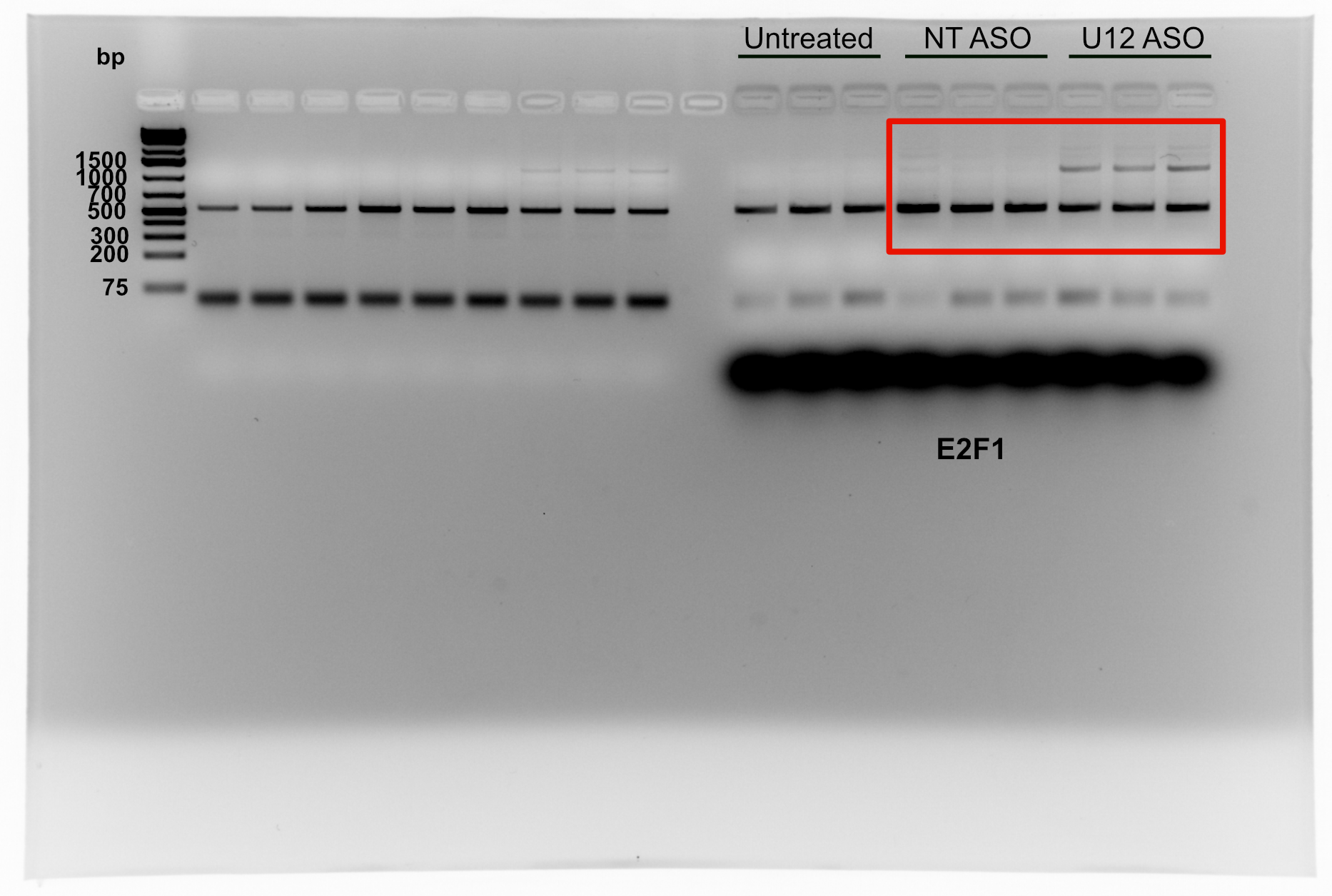

Supplement: Supplementary file 9 — Source data Fig. 6 [file 44319_2025_511_MOESM9_ESM.zip › Figure 6/Fig 6A Gel images/E2F1.tiff]

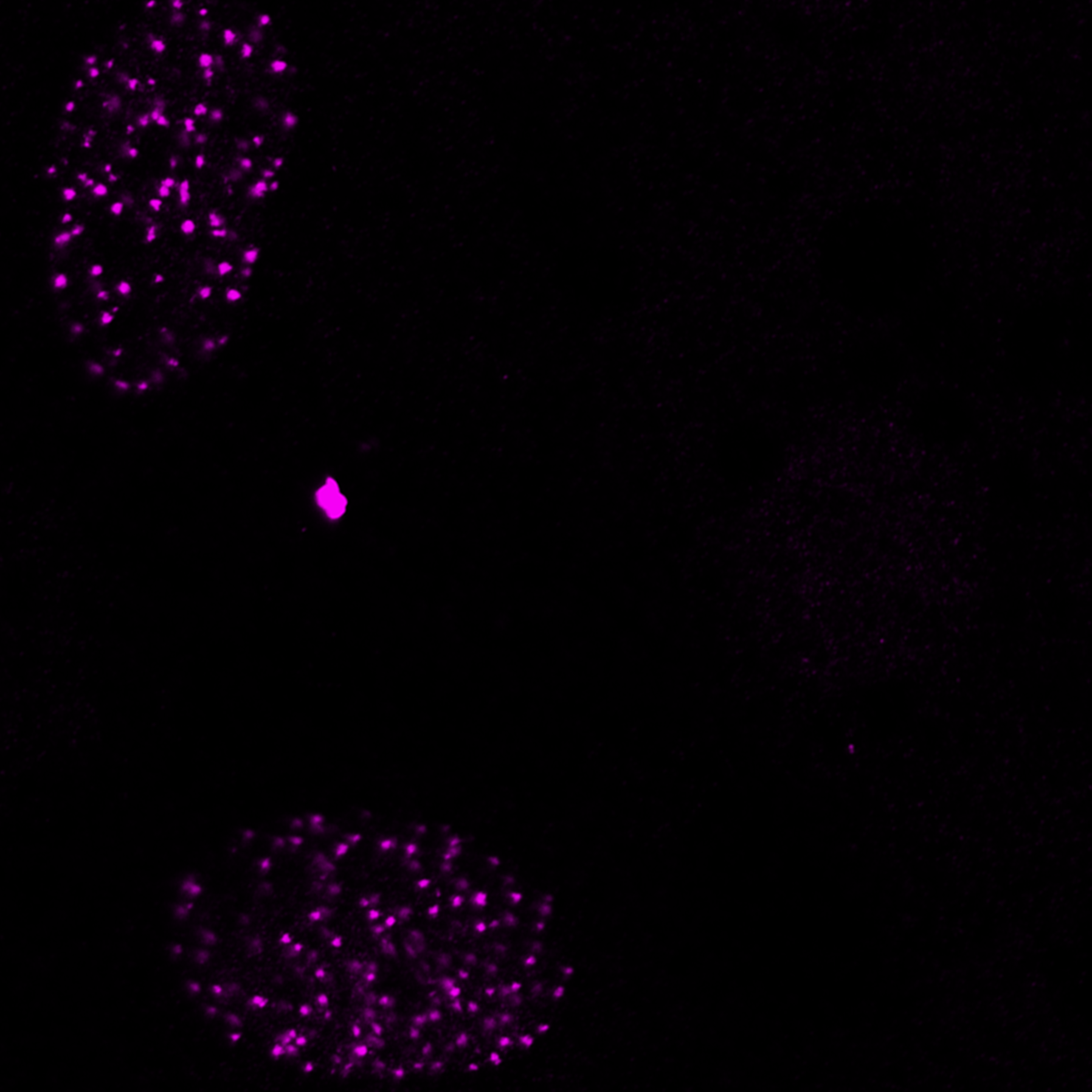

Supplement: Supplementary file 9 — Source data Fig. 6 [file 44319_2025_511_MOESM9_ESM.zip › Figure 6/Fig 6D_Image Files/10 nM U12 ASO/Image 30_53BP1_Airyscan Processing.czi - C=0.tif]

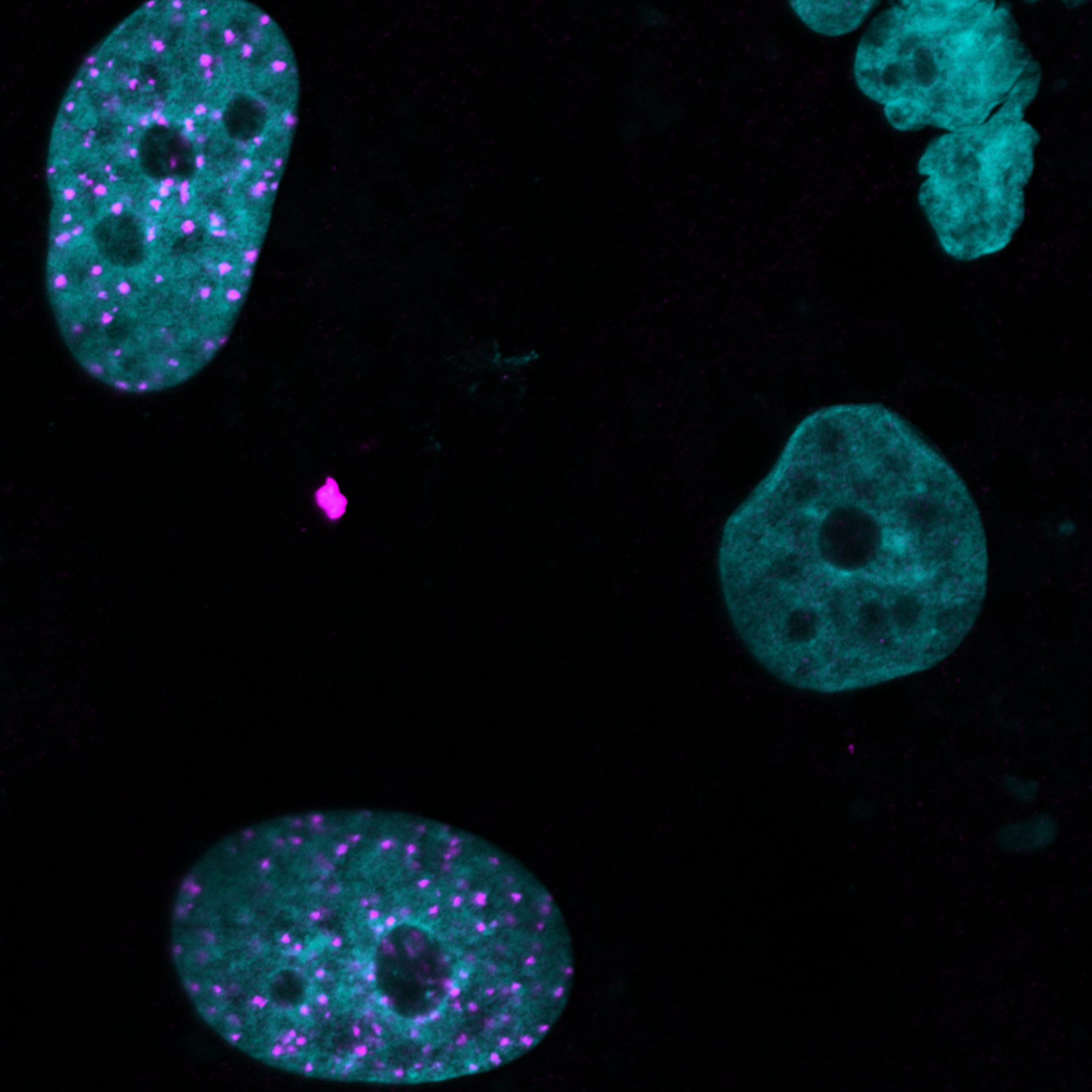

Supplement: Supplementary file 9 — Source data Fig. 6 [file 44319_2025_511_MOESM9_ESM.zip › Figure 6/Fig 6D_Image Files/10 nM U12 ASO/Image30_merge.tif]

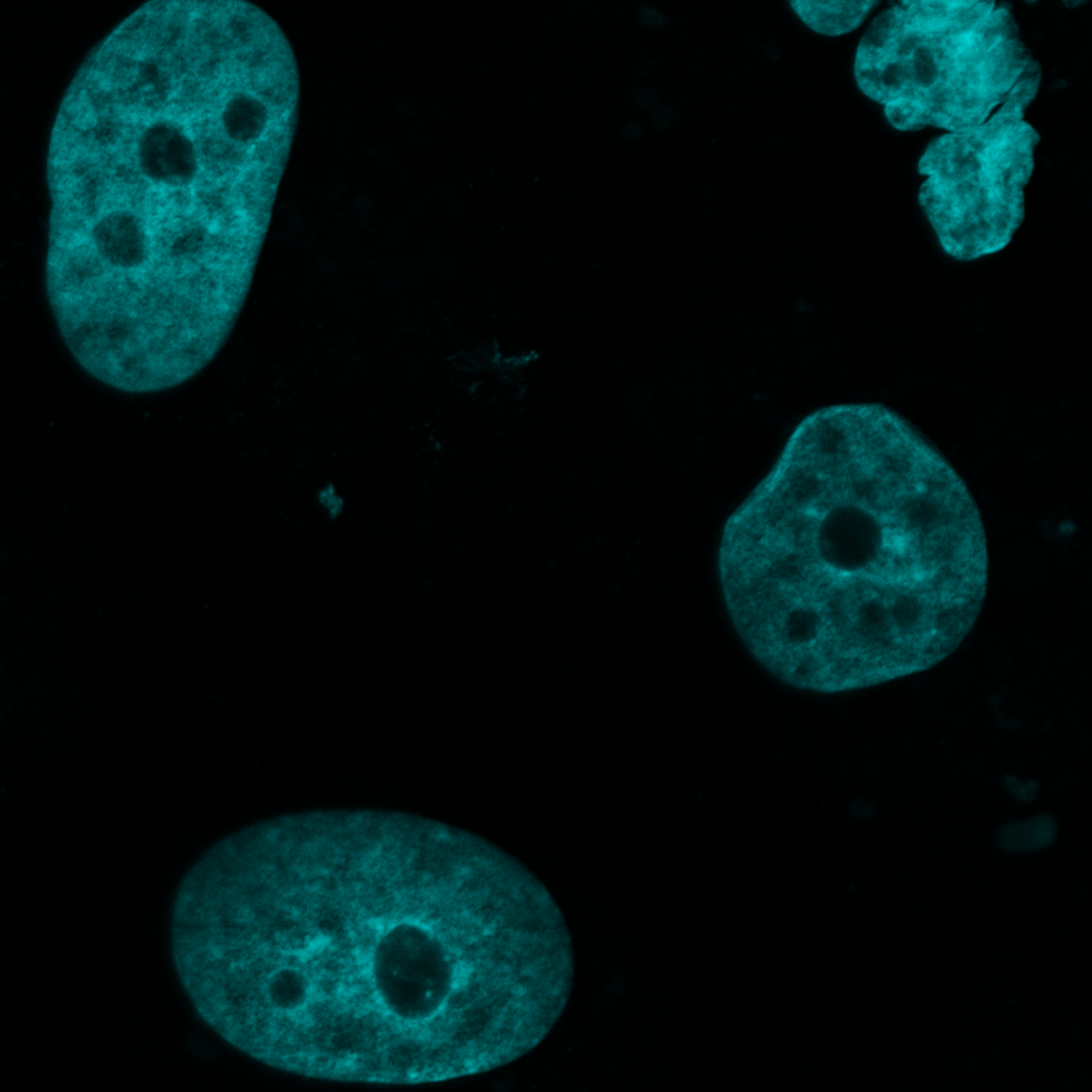

Supplement: Supplementary file 9 — Source data Fig. 6 [file 44319_2025_511_MOESM9_ESM.zip › Figure 6/Fig 6D_Image Files/10 nM U12 ASO/Image 30_DAPI_Airyscan Processing.czi - C=1.tif]

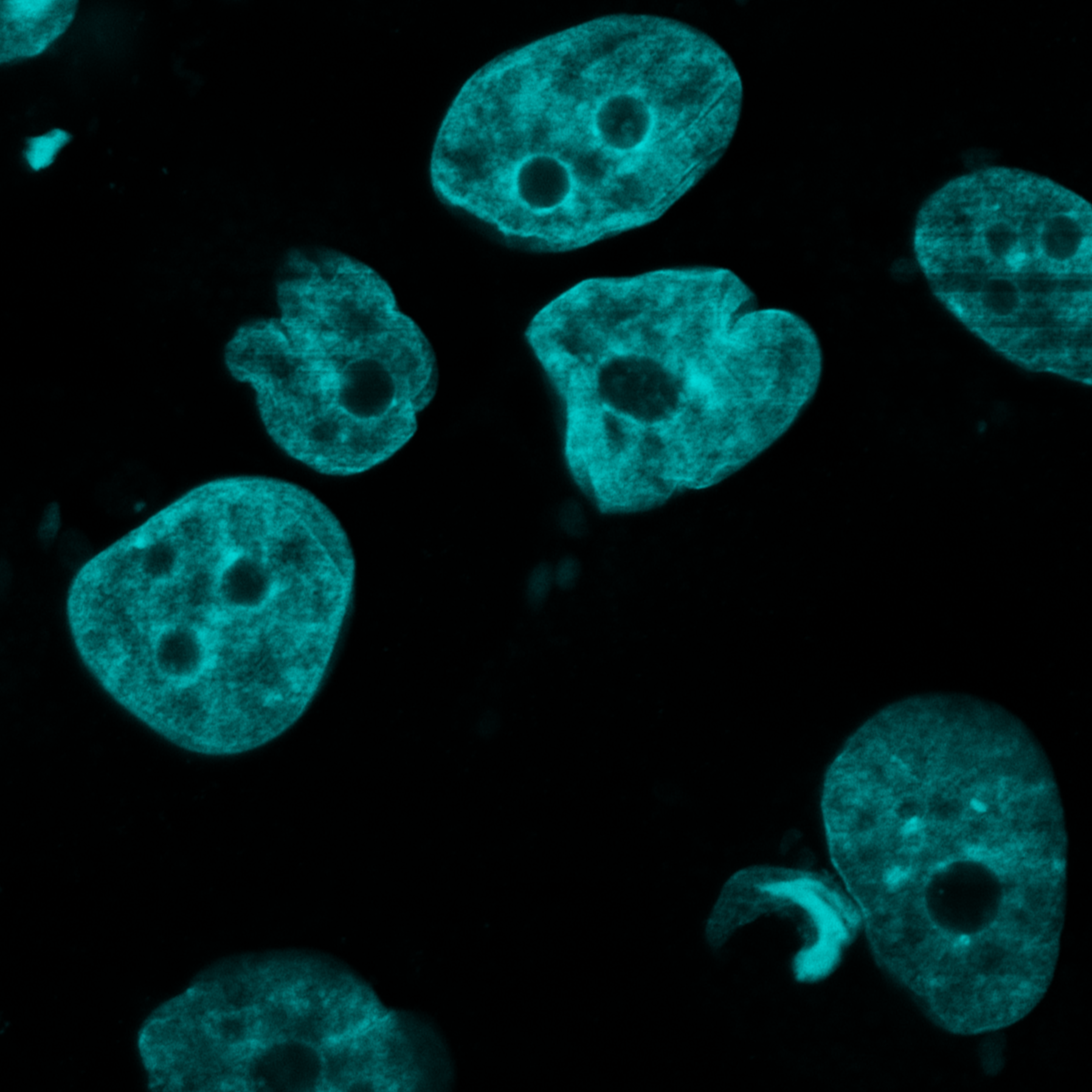

Supplement: Supplementary file 9 — Source data Fig. 6 [file 44319_2025_511_MOESM9_ESM.zip › Figure 6/Fig 6D_Image Files/10 nM U12 ASO/Image 28_DAPI_Airyscan Processing.czi - C=1.tif]

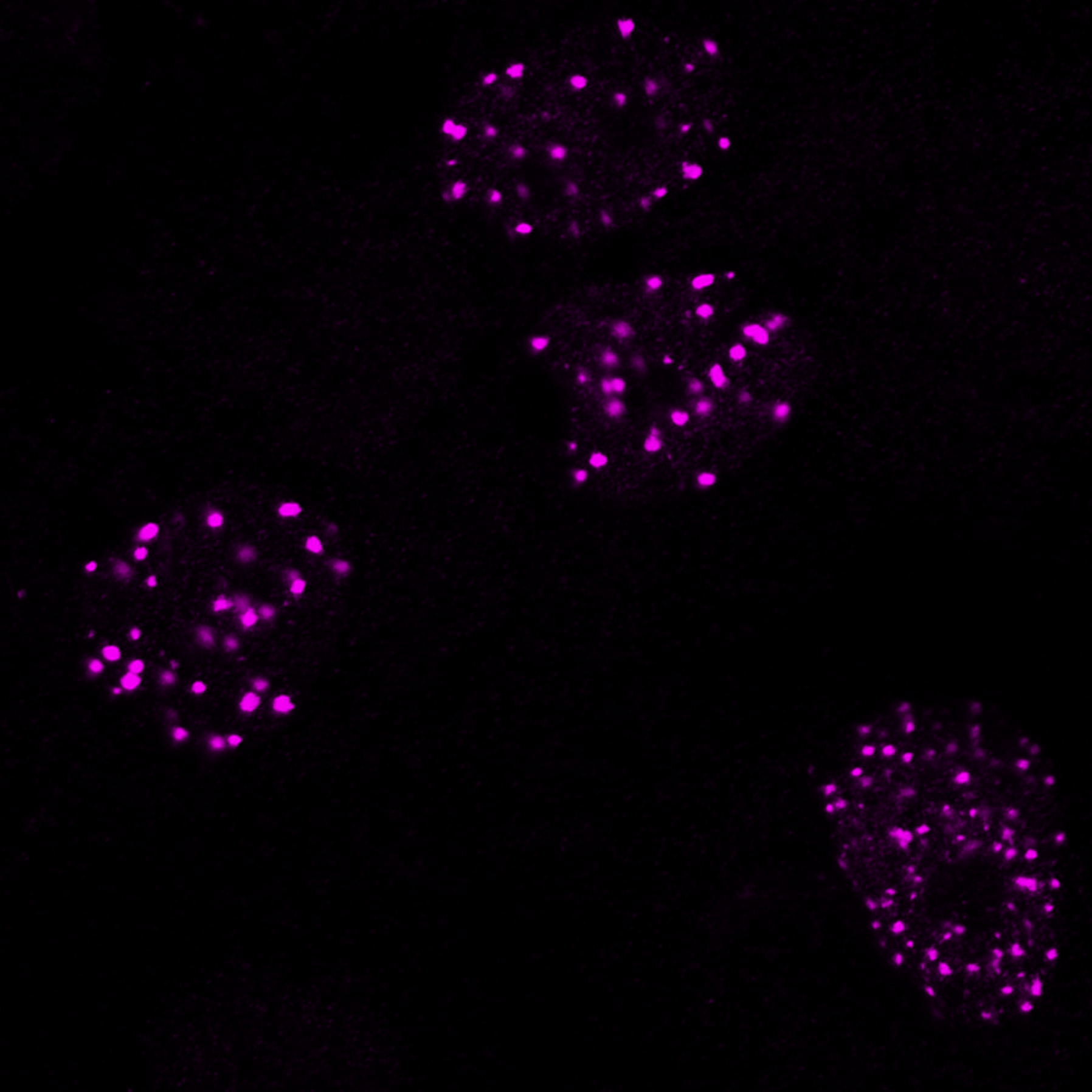

Supplement: Supplementary file 9 — Source data Fig. 6 [file 44319_2025_511_MOESM9_ESM.zip › Figure 6/Fig 6D_Image Files/10 nM U12 ASO/Image 28_53BP1_Airyscan Processing.czi - C=0.tif]

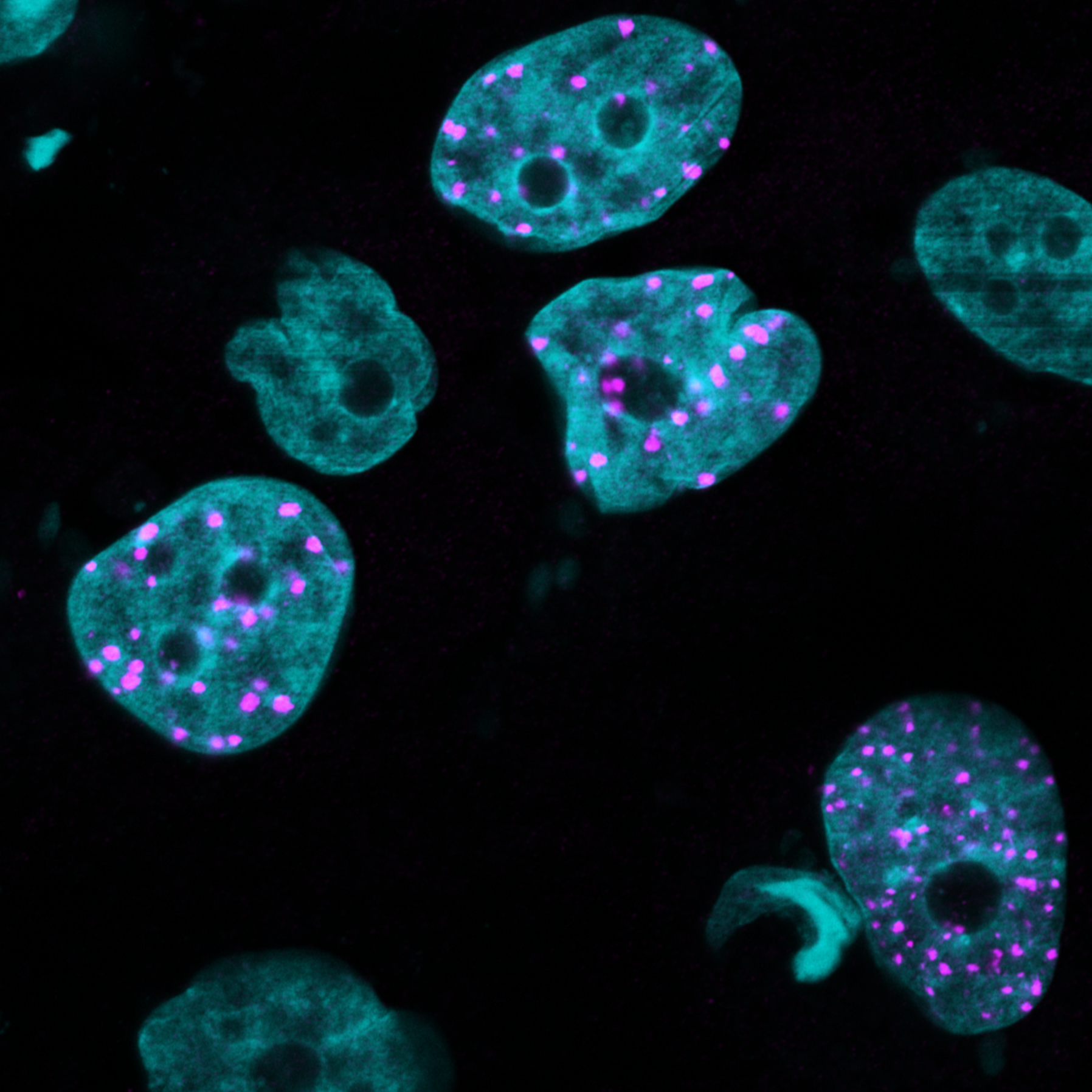

Supplement: Supplementary file 9 — Source data Fig. 6 [file 44319_2025_511_MOESM9_ESM.zip › Figure 6/Fig 6D_Image Files/10 nM U12 ASO/53BP1_28_merge.tif]

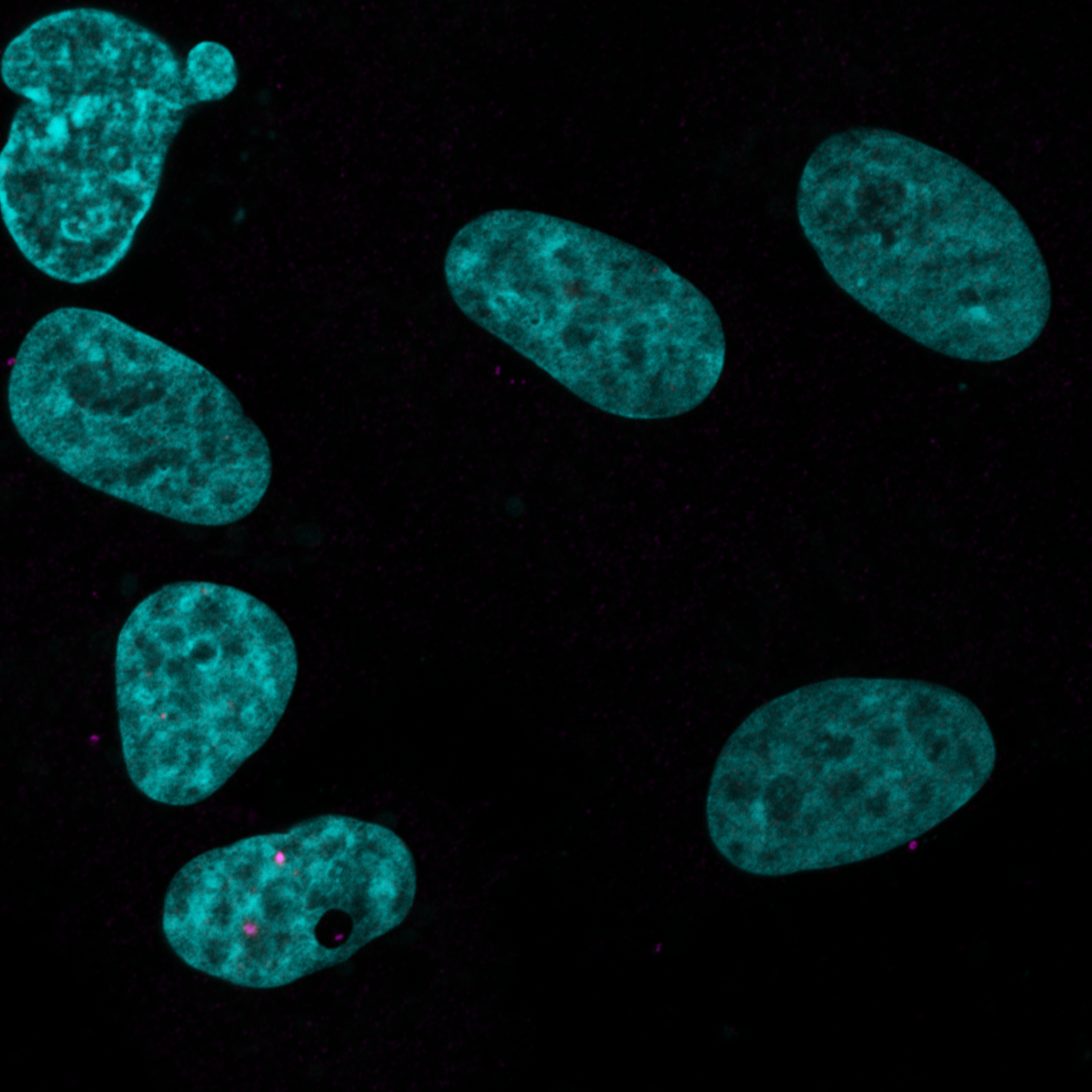

Supplement: Supplementary file 9 — Source data Fig. 6 [file 44319_2025_511_MOESM9_ESM.zip › Figure 6/Fig 6D_Image Files/10 nM NT ASO/Image25_merge.tif]

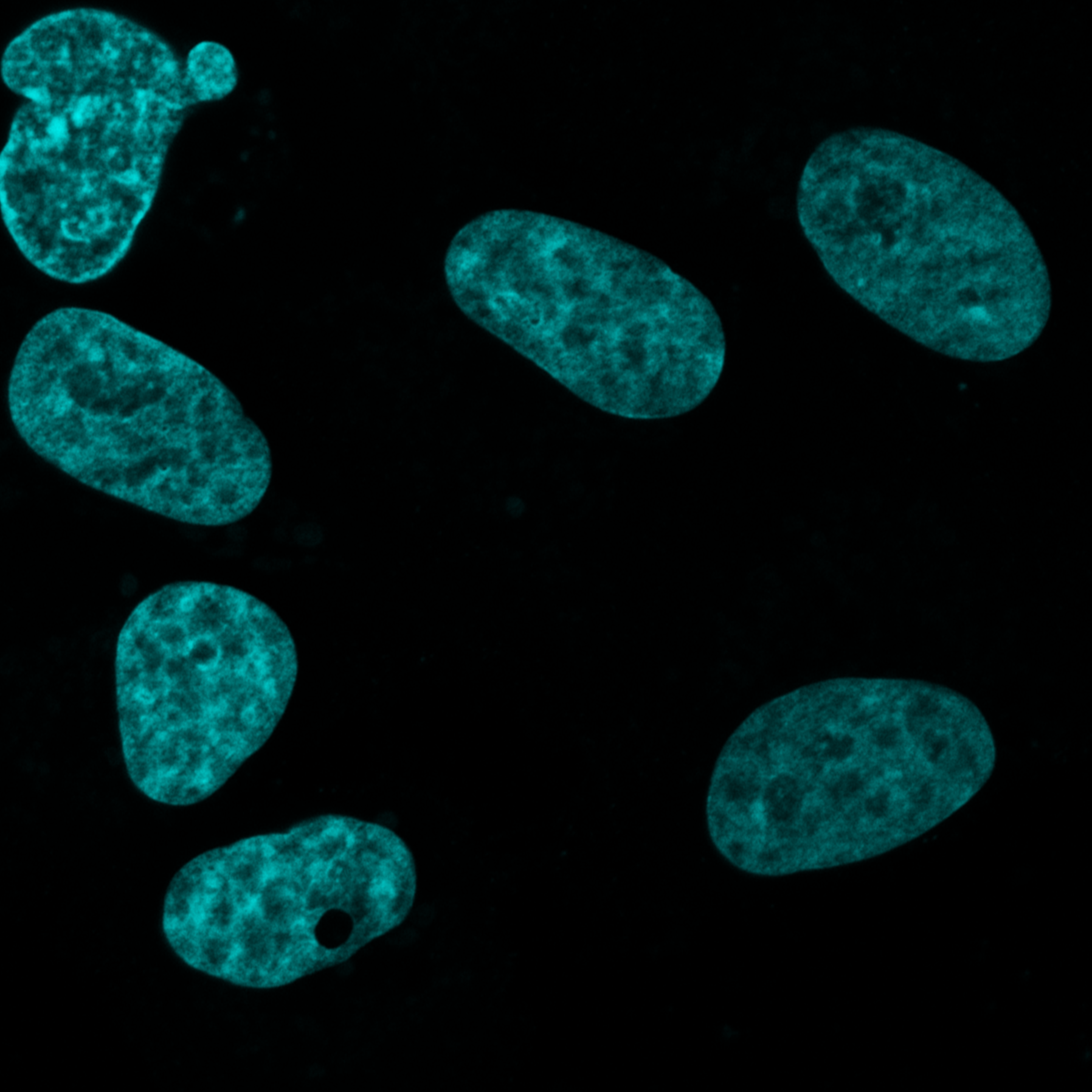

Supplement: Supplementary file 9 — Source data Fig. 6 [file 44319_2025_511_MOESM9_ESM.zip › Figure 6/Fig 6D_Image Files/10 nM NT ASO/Image 25_DAPI_Airyscan Processing.czi - C=1.tif]

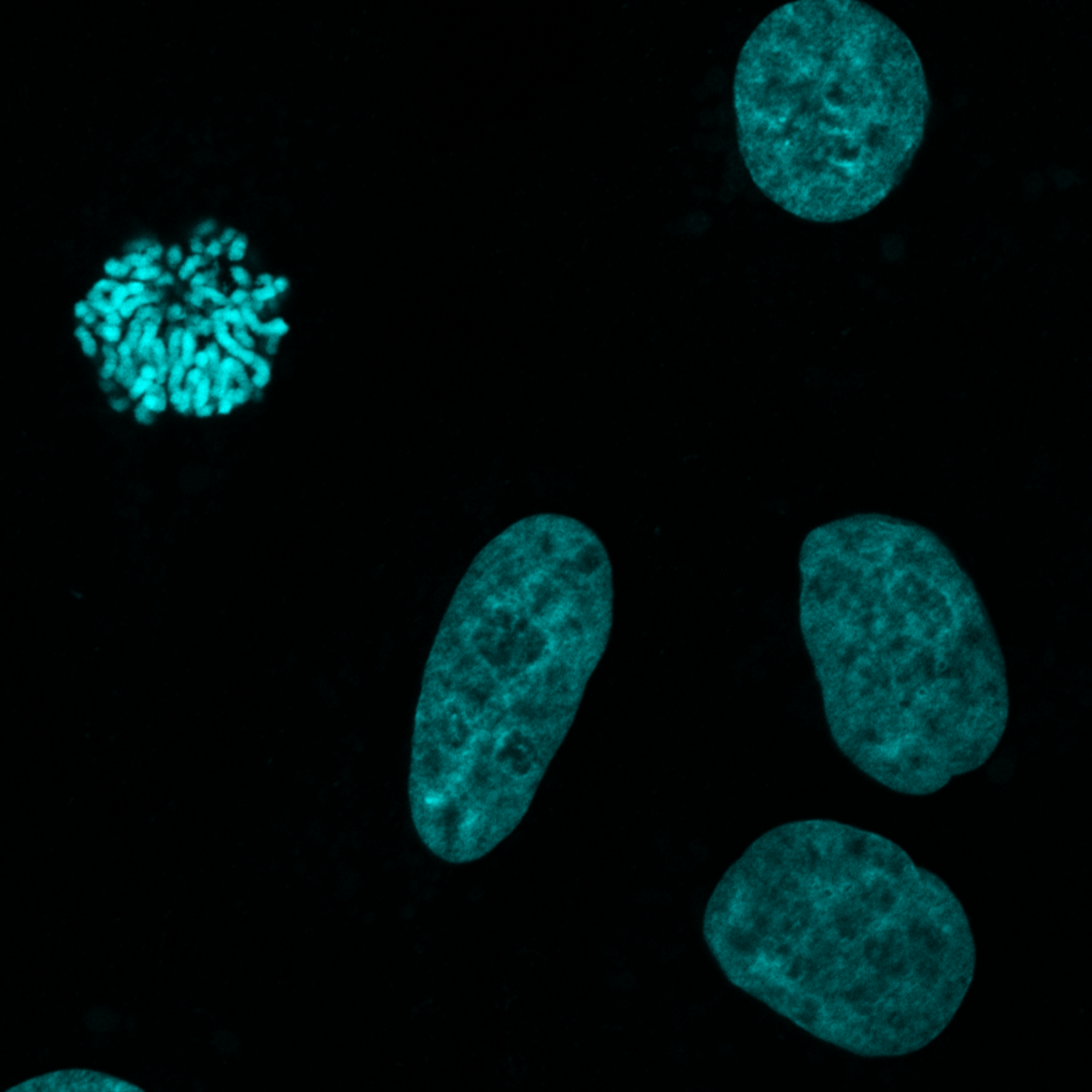

Supplement: Supplementary file 9 — Source data Fig. 6 [file 44319_2025_511_MOESM9_ESM.zip › Figure 6/Fig 6D_Image Files/10 nM NT ASO/Image 22_DAPI_Airyscan Processing.czi - C=1.tif]

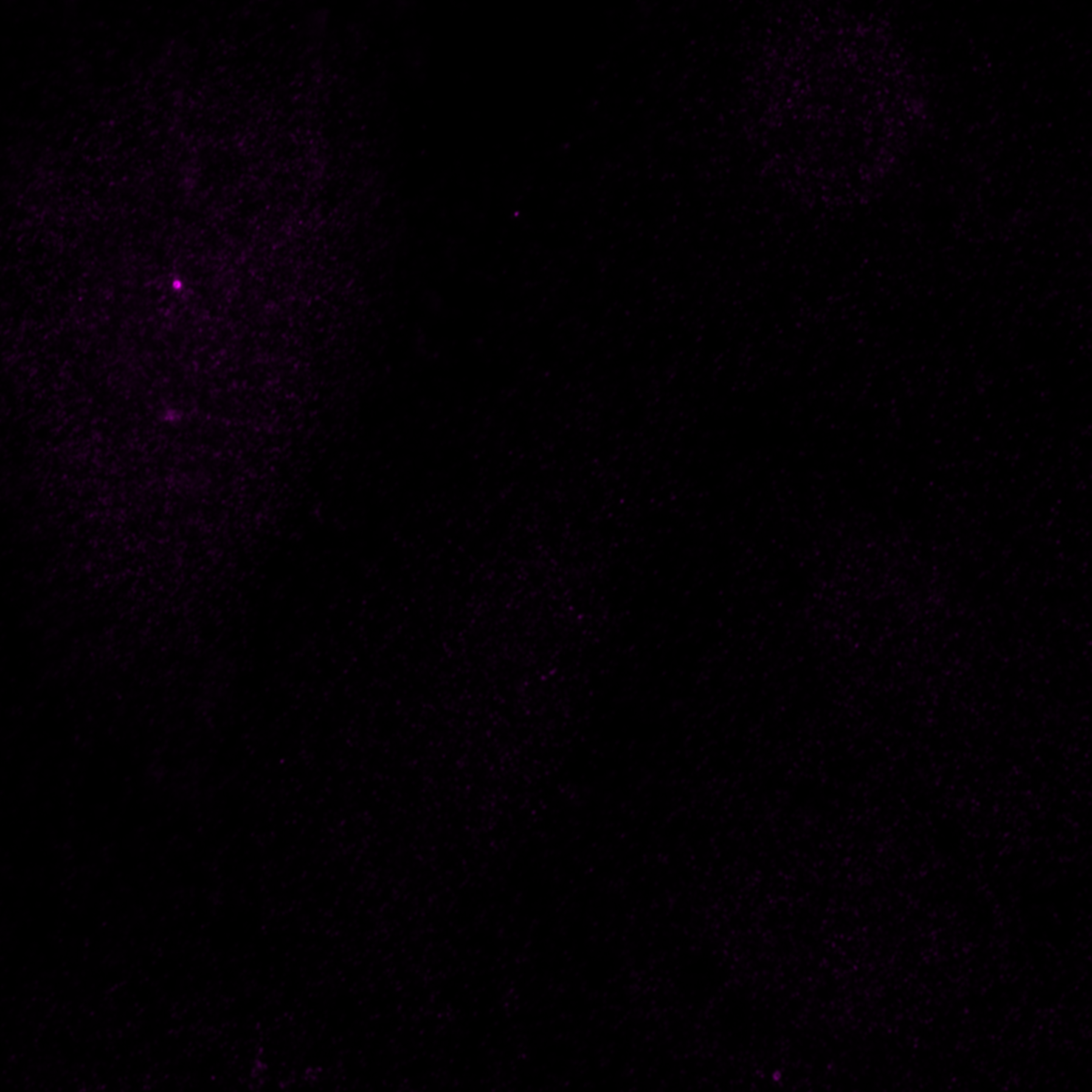

Supplement: Supplementary file 9 — Source data Fig. 6 [file 44319_2025_511_MOESM9_ESM.zip › Figure 6/Fig 6D_Image Files/10 nM NT ASO/Image 22_53BP1_Airyscan Processing.czi - C=0.tif]

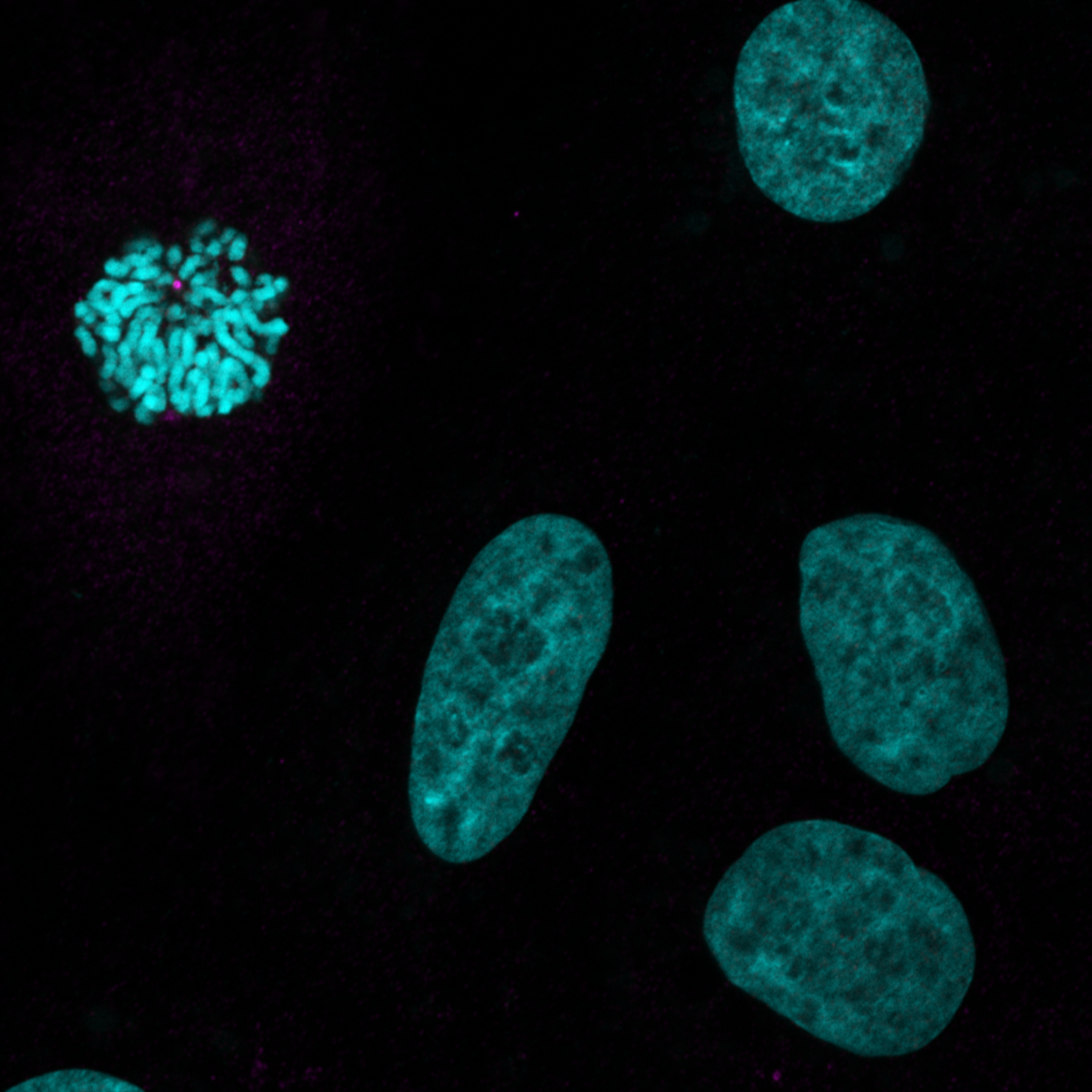

Supplement: Supplementary file 9 — Source data Fig. 6 [file 44319_2025_511_MOESM9_ESM.zip › Figure 6/Fig 6D_Image Files/10 nM NT ASO/52BP1_22_merge.tif]

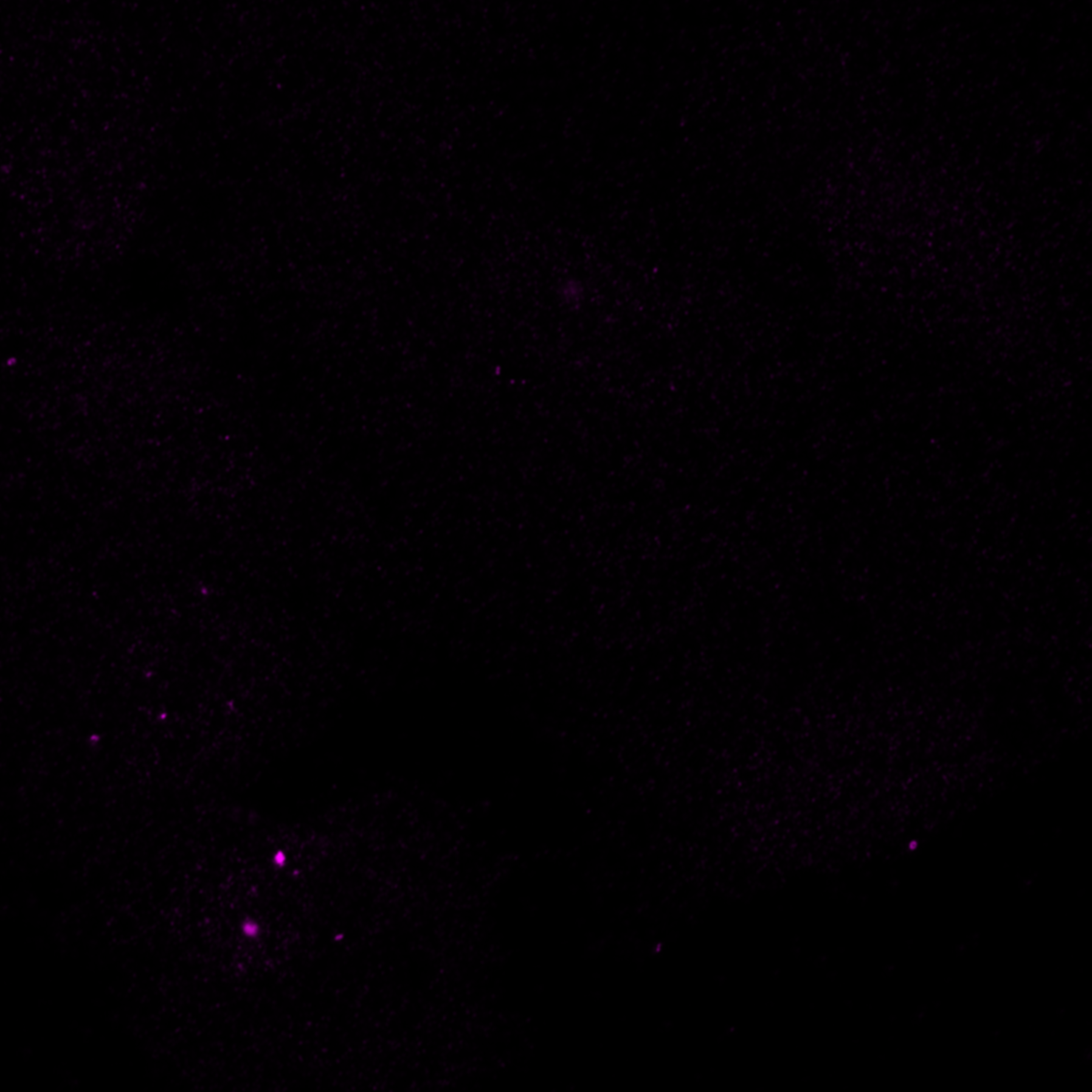

Supplement: Supplementary file 9 — Source data Fig. 6 [file 44319_2025_511_MOESM9_ESM.zip › Figure 6/Fig 6D_Image Files/10 nM NT ASO/Image 25_53BP1_Airyscan Processing.czi - C=0.tif]
