# Supplementary material for: Inhibition of the minor spliceosome restricts the growth of a broad spectrum of cancers
Source: EMBO Rep. 2025 Jul 7;26(15):3937–69. doi: 10.1038/s44319-025-00511-8 (PMC12332006; doi:10.1038/s44319-025-00511-8)
Supplement: Supplementary file 10 — Expanded View Figures [file 44319_2025_511_MOESM10_ESM.pdf]

Expanded View Figures

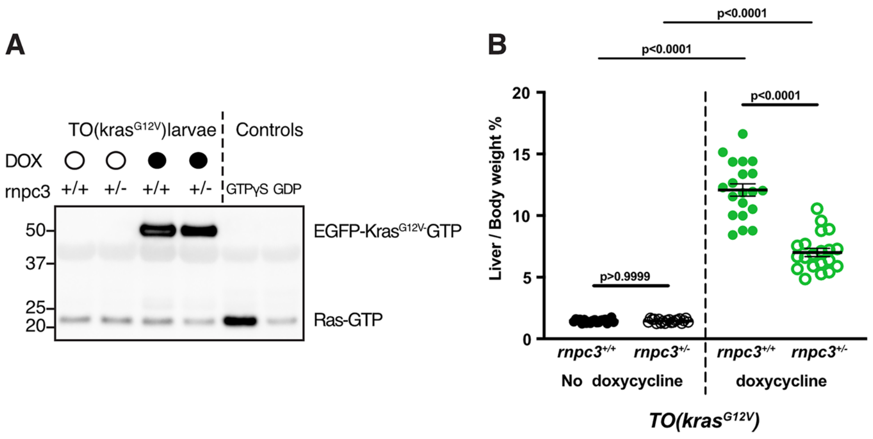

Figure EV1. Heterozygous loss of *rnpc3* reduces tumour burden in a *kras*^{G12V}-driven zebrafish model of hepatocellular carcinoma (HCC).

(**A**) Western blot of active Ras-GTP protein following active Ras pull-down from *TO(kras*^{G12V}*)* larvae of the indicated *rnpc3* genotype. (**B**) *rnpc3*$^{+/+}$ and *rnpc3*$^{+/-}$ liver/body mass ratio (%) of adult male *TO(kras*^{G12V}*)* (3.5–4 months of age) with and without dox treatment. $n = 20$, 2 pooled independent experiments. Mean ± SEM, significance was assessed by one-way ANOVA with Tukey's multiple comparison test.

 © The Author(s)

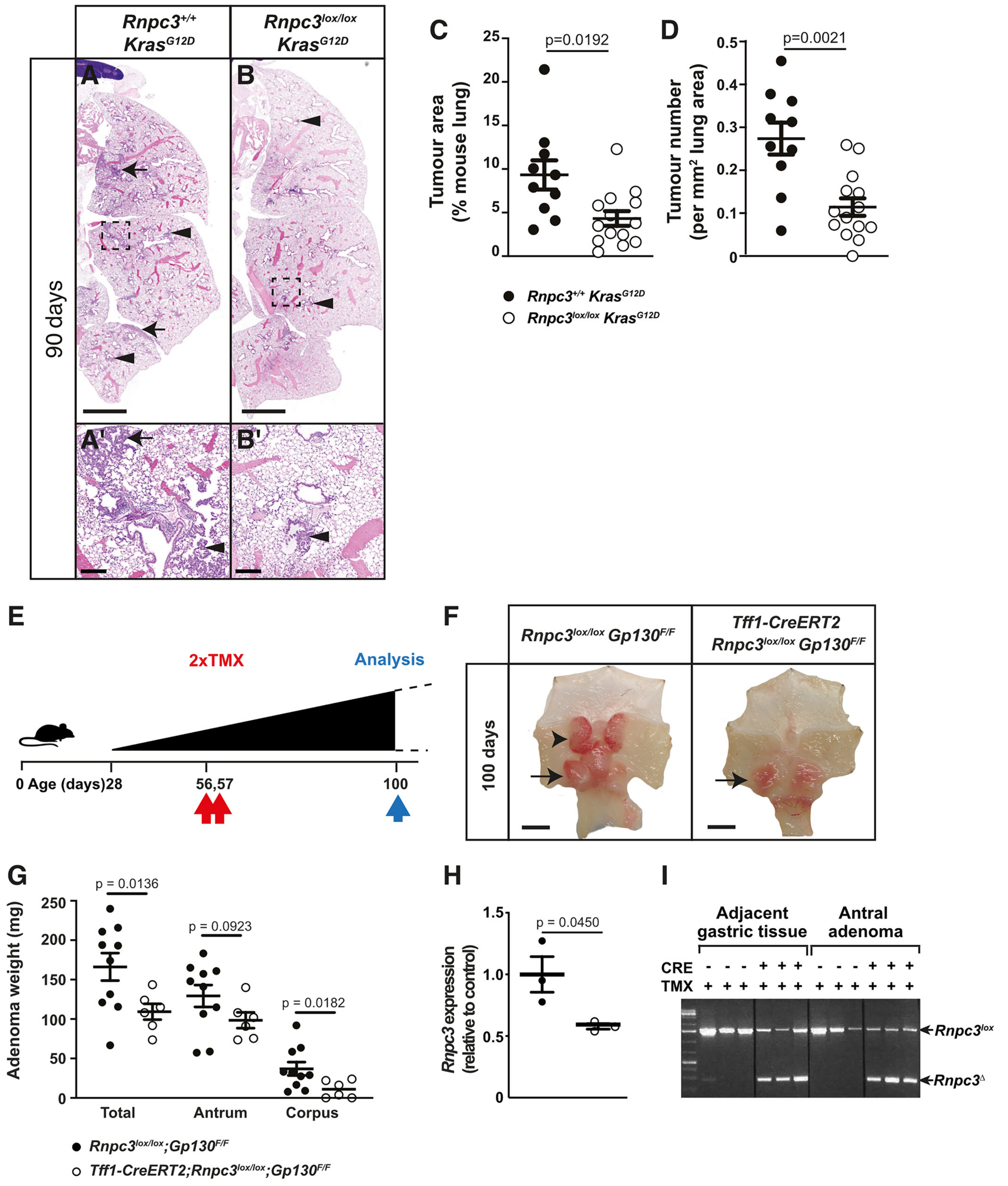
Rnpc3+/+ KrasG12D
Rnpc3lox/lox KrasG12D
90 days
A
B
A'
B'
C
D
p=0.0192
p=0.0021
Tumour area (% mouse lung)
Tumour number (per mm² lung area)
Rnpc3+/+ KrasG12D
Rnpc3lox/lox KrasG12D
E
2xTMX
Analysis
0 Age (days)28
56,57
100
F
Rnpc3lox/lox Gp130F/F
Tff1-CreERT2 Rnpc3lox/lox Gp130F/F
100 days
G
p = 0.0136
p = 0.0923
p = 0.0182
Adenoma weight (mg)
Total
Antrum
Corpus
Rnpc3lox/lox;Gp130F/F
Tff1-CreERT2;Rnpc3lox/lox;Gp130F/F
H
p = 0.0450
Rnpc3 expression (relative to control)
I
Adjacent gastric tissue
Antral adenoma
CRE
TMX
Rnpc3lox
Rnpc3Δ

Figure EV2. Recombination of *Rnpc3*^{lox} alleles in mice reduces tumour burden in *Kras*^{G12D}-driven lung adenocarcinoma and a *Gp130*$^{F/F}$ gastric adenoma model.

(**A**, **B**) Histological analysis of lung hyperplasia. Areas of AAH (atypical adenomatous hyperplasia; arrows) and bronchial hyperplasia (arrowheads) were observed in the lungs 90 d after adenoviral Cre recombinase administration. Scale bar is 2 mm. (**A′**, **B′**) Higher magnification images of region in dashed box. Scale bar is 200 µm. (**C**, **D**) Quantitation of the frequency and area of the hyperplastic lesions. Data are expressed as mean ± SEM, $n = 10$ or 14 per genotype. Significance was assessed using an unpaired Student's *t* test with Welch's correction. (**E**) Schematic diagram of gastric adenoma formation in *Gp130*$^{F/F}$ mice and treatment with tamoxifen (2 x TMX) on days 56 and 57 (red arrows) to induce recombination of *Rnpc3*^{lox} alleles. (**F**) Representative stomachs with adenomas in the corpus (arrowhead) and antrum (arrow). Scale bar is 5 mm. (**G**) Quantitation of adenoma weight. Data are expressed as mean ± SEM, $n = 6$ or 10 per genotype. Significance was assessed using an unpaired Student's *t* test with Welch's correction. (**H**) RT-qPCR analysis of *Rnpc3* mRNA in single antral adenomas from three independent mice per genotype. Data are expressed as mean ± SEM. Significance was evaluated with a two-tailed Student's *t* test. (**I**) PCR of genomic DNA for *Rnpc3* alleles in normal glandular stomach and adenomas harvested from TMX-treated, *Tff1-CreERT2;Rnpc3*$^{lox/lox}$*;Gp130*$^{F/F}$ mice, $n = 3$.

 © The Author(s)

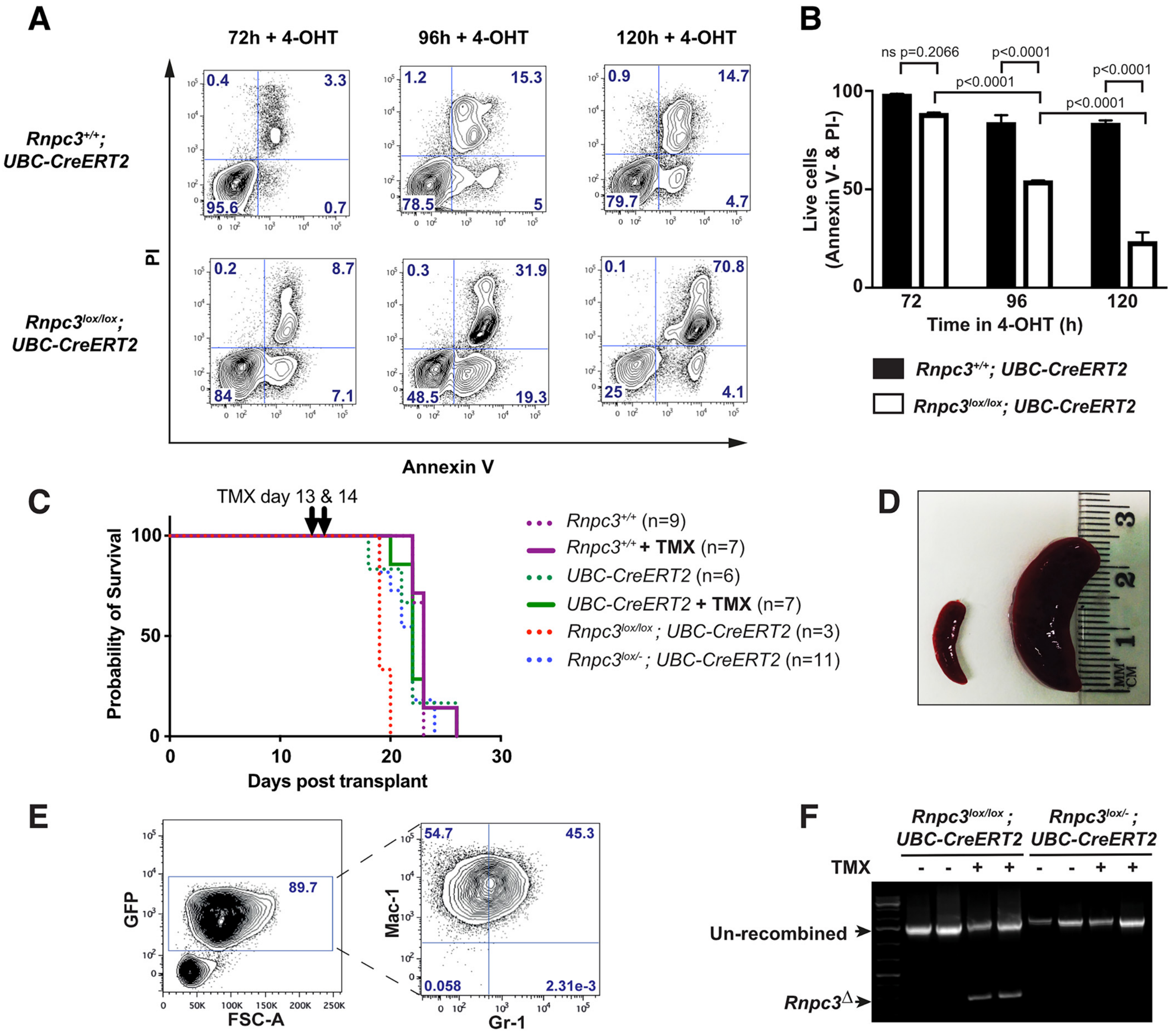

Figure EV3. Recombination of the *Rnpc3* locus in MLL-ENL AML cells in vitro reduces cell viability.

(**A**) Representative fluorescence-activated cell sorting (FACS) plots of cell viability using AnnexinV and PI staining. Numbers in each quadrant indicate the percentage of cells in each category. (**B**) Quantitation of the percentage of viable (AnnexinV$^-$, PI$^-$) cells over time enumerated by FACS. Percentage viable cells expressed as mean ± SEM, $n = 3$ for all cohorts. Significant differences were assessed with a one-way ANOVA with Tukey's multiple comparison test. (**C**) Kaplan–Meier survival plot of mice harbouring tertiary transplants of MLL-ENL AML cells with all control genotypes in the presence (solid line) and absence (dotted line) of TMX treatment 13 and 14 d (black arrows) following transplantation. There is no significant difference in median survival (19–23 d) between all control cohorts (i.e. no *Rnpc3*^{lox} alleles and no TMX treatment), $n = 311$. Significance was assessed with a Mantel–Cox test. (**D**) Spleen from a WT mouse not transplanted with AML cells next to a spleen harvested from a mouse transplanted with *UBC-CreERT2;Rnpc3*$^{lox/lox}$ AML cells and treated with TMX. (**E**) Example FACS analysis of *UBC-CreERT2;Rnpc3*$^{lox/lox}$ AML cells. (**F**) Genomic analysis of the *Rnpc3* locus in tertiary transplanted AML cells.

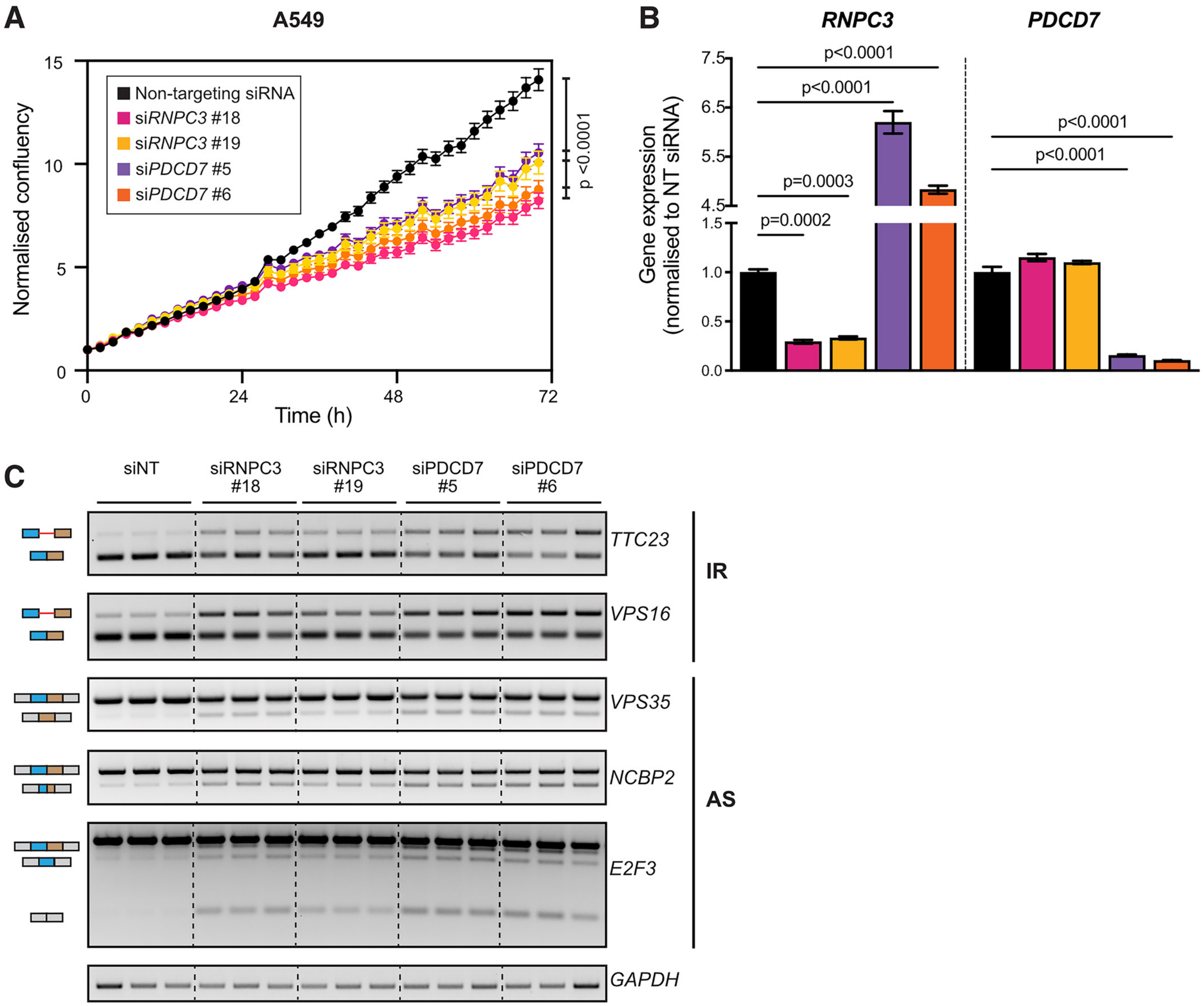

Figure EV4. Analysis of cell proliferation and aberrant splicing of MIGs in A549 cells treated with si*RNPC3* and si*PDCD7* for 72 h.

(**A**) Quantification of A549 cell growth over 72 h treatment with non-targeting (NT), two independent *RNPC3* or 2 independent *PDCD7* siRNAs. Data are represented as mean ± SEM ($n = 3$, 25 images per well, every hour). Significance was assessed by 2way ANOVA with Dunnett's multiple comparisons test. Note: the siNT and si*RNPC3* data are the same as shown in Fig. 5B. (**B**) RT-qPCR analysis of *RNPC3* and *PDCD7* mRNA in A549 cells after 72 h treatment with siRNAs. Data are represented as mean ± SEM ($n = 3$), significance was tested using a one-way ANOVA with Tukey's multiple comparisons test. (**C**) RT-PCR analysis of example MIG splicing changes, identified in A549 cells treated with si*RNPC3* or si*PDCD7* for 72 h. Schematised IR and AS events are represented on the left with the minor intron in red and the flanking upstream and downstream exons in blue and orange, respectively. Exons not separated by a minor intron are grey.

 © The Author(s)

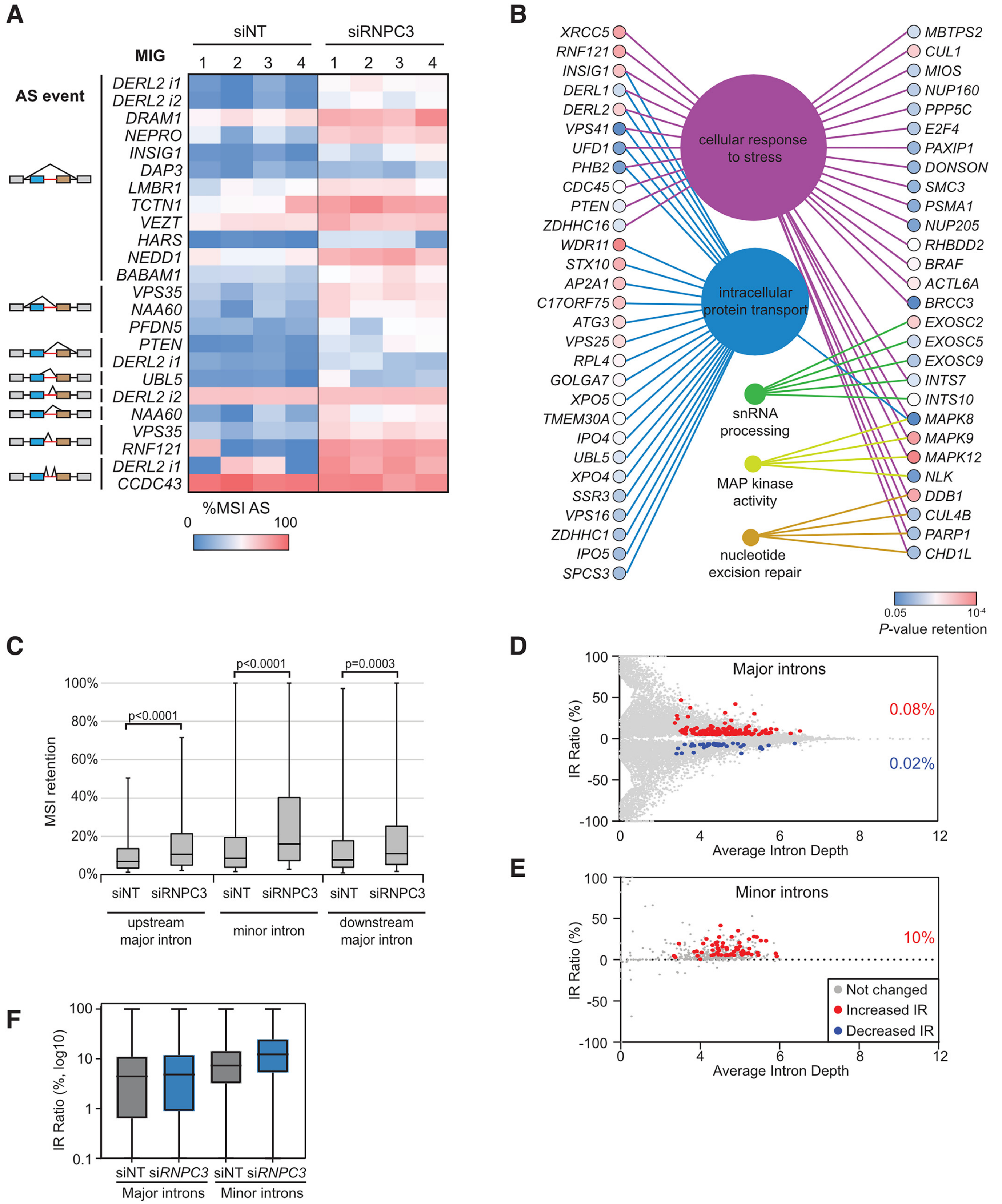
A
siNT
siRNPC3
MIG
1 2 3 4 1 2 3 4
AS event
DERL2 i1
DERL2 i2
DRAM1
NEPRO
INSIG1
DAP3
LMBR1
TCTN1
VEZT
HARS
NEDD1
BABAM1
VPS35
NAA60
PFDN5
PTEN
DERL2 i1
UBL5
DERL2 i2
NAA60
VPS35
RNF121
DERL2 i1
CCDC43
%MSI AS
0
100
B
XRCC5
RNF121
INSIG1
DERL1
DERL2
VPS41
UFD1
PHB2
CDC45
PTEN
ZDHHC16
WDR11
STX10
AP2A1
C17ORF75
ATG3
VPS25
RPL4
GOLGA7
XPO5
TMEM30A
IPO4
UBL5
XPO4
SSR3
VPS16
ZDHHC1
IPO5
SPCS3
cellular response to stress
intracellular protein transport
snRNA processing
MAP kinase activity
nucleotide excision repair
MBTPS2
CUL1
MIOS
NUP160
PPP5C
E2F4
PAXIP1
DONSON
SMC3
PSMA1
NUP205
RHBDD2
BRAF
ACTL6A
BRCC3
EXOSC2
EXOSC5
EXOSC9
INTS7
INTS10
MAPK8
MAPK9
MAPK12
NLK
DDB1
CUL4B
PARP1
CHD1L
0.05
10-4
P-value retention
C
MSI retention
100%
80%
60%
40%
20%
0%
p<0.0001
p<0.0001
p=0.0003
siNT siRNPC3 siNT siRNPC3 siNT siRNPC3
upstream major intron
minor intron
downstream major intron
D
Major introns
IR Ratio (%)
100
50
0
-50
-100
0.08%
0.02%
0 4 6 8 12
Average Intron Depth
E
Minor introns
IR Ratio (%)
100
50
0
-50
-100
10%
Not changed
Increased IR
Decreased IR
0 4 6 8 12
Average Intron Depth
F
IR Ratio (%, log10)
100
10
1
0.1
siNT siRNPC3 siNT siRNPC3
Major introns
Minor introns

Figure EV5. RNAseq analysis of A549 cells treated with si*RNPC3* for 72 h reveals defects in MIG splicing.

(**A**) Heatmap of RNAseq identified MIG AS events. (**B**) Selected enriched GO terms of MIGs with significant intron retention. Significance was assessed by Welch's *t* test. (**C**) Box plot of intron retention at minor introns and their upstream and downstream major introns. Significance was assessed by Mann–Whitney *U* test, $n = 4$ replicate RNAseq samples/genotype. (**D**, **E**) Transcriptome-wide IRFinder analysis showing median IR for all introns. (**D**) Major introns showing significantly upregulated IR (solid red circles) and significantly downregulated IR (solid blue circles). (**E**) Minor introns showing significantly upregulated IR (solid red circles). For an intron to be called as significantly retained in si*RNPC3* samples, we required the following, FDR JQ 0.05, 100% coverage of the intron in si*RNPC3* samples, 10% IRratio in si*RNPC3* and 5% IRratio over NT samples. Likewise, for an intron to be significantly retained in NT samples, FDR JQ 0.05, 100% coverage across the intron in NT samples, 10% IRratio in NT cells and 5% IRratio over si*RNPC3* samples. (**F**) Box plot of intron retention (% IRratio log10) across 234,057 major and 599 minor introns. Significance was assessed by paired *t* test.

 © The Author(s)

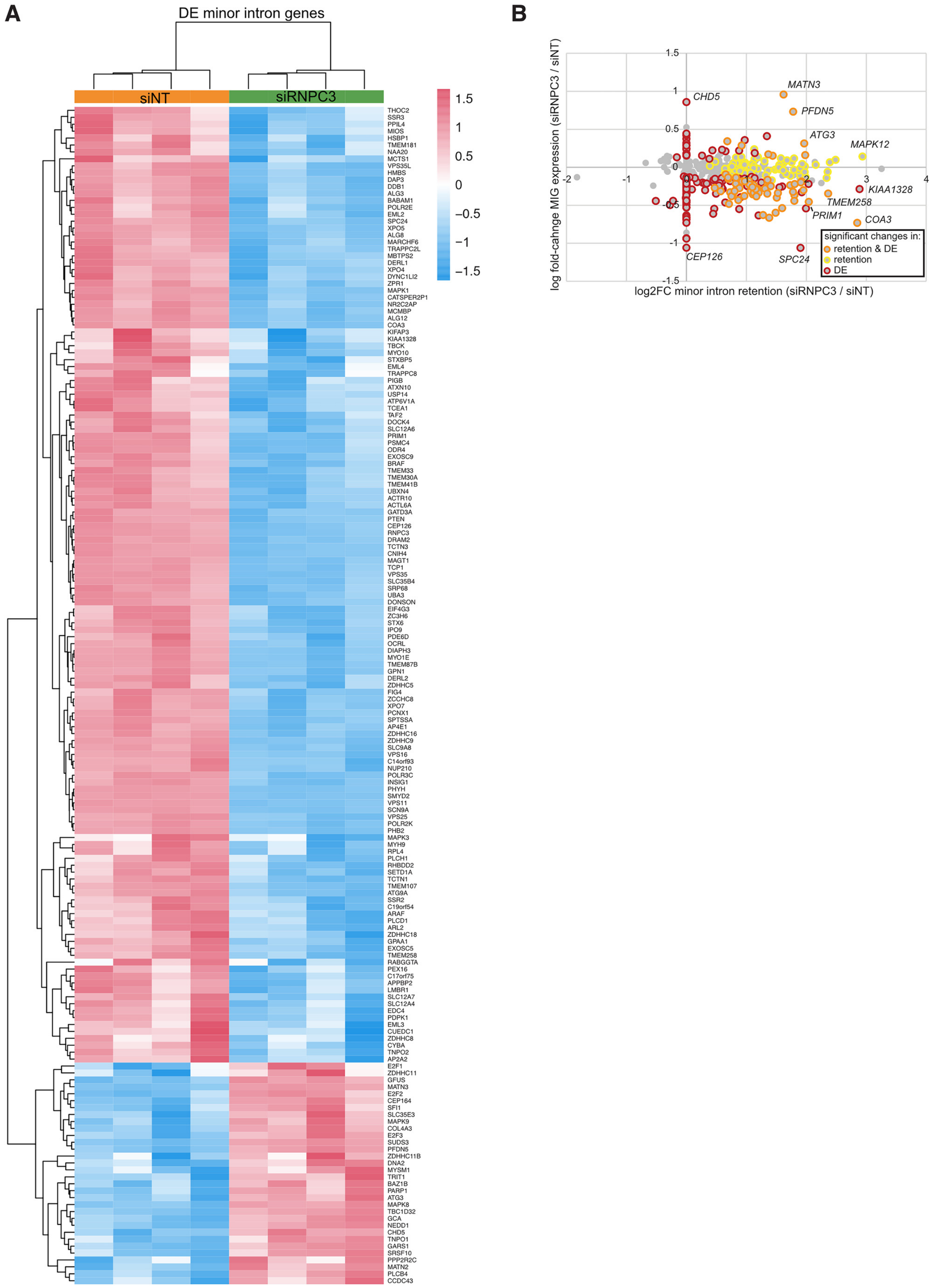
A
DE minor intron genes
siNT
siRNPC3
1.5
1
0.5
0
−0.5
−1
−1.5
THOC2
SSR3
PPIL4
MIOS
HSBP1
TMEM181
NAA20
MCTS1
VPS35L
HMBS
DAP3
DDB1
ALG3
BABAM1
POLR2E
EML2
SPC24
XPO5
ALG8
MARCHF6
TRAPPC2L
MBTPS2
DERL1
XPO4
DYNC1LI2
ZPR1
MAPK1
CATSPER2P1
NR2C2AP
MCMBP
ALG12
COA3
KIFAP3
KIAA1328
TBCK
MYO10
STXBP5
EML4
TRAPPC8
PIGB
ATXN10
USP14
ATP6V1A
TCEA1
TAF2
DOCK4
SLC12A6
PRIM1
PSMC4
ODR4
EXOSC9
BRAF
TMEM33
TMEM30A
TMEM41B
UBXN4
ACTR10
ACTL6A
GATD3A
PTEN
CEP126
RNPC3
DRAM2
TCTN3
CNIH4
MAGT1
TCP1
VPS35
SLC35B4
SRP68
UBA3
DONSON
EIF4G3
ZC3H6
STX6
IPO9
PDE6D
OCRL
DIAPH3
MYO1E
TMEM87B
GPN1
DERL2
ZDHHC5
FIG4
ZCCHC8
XPO7
PCNX1
SPTSSA
AP4E1
ZDHHC16
ZDHHC9
SLC9A8
VPS16
C14orf93
NUP210
POLR3C
INSIG1
PHYH
SMYD2
VPS11
SCN9A
VPS25
POLR2K
PHB2
MAPK3
MYH9
RPL4
PLCH1
RHBDD2
SETD1A
TCTN1
TMEM107
ATG9A
SSR2
C19orf54
ARAF
PLCD1
ARL2
ZDHHC18
GPAA1
EXOSC5
TMEM258
RABGGTA
PEX16
C17orf75
APPBP2
LMBR1
SLC12A7
SLC12A4
EDC4
PDPK1
EML3
CUEDC1
ZDHHC8
CYBA
TNPO2
AP2A2
E2F1
ZDHHC11
GFUS
MATN3
E2F2
CEP164
SFI1
SLC35E3
MAPK9
COL4A3
E2F3
SUDS3
PFDN5
ZDHHC11B
DNA2
MYSM1
TRIT1
BAZ1B
PARP1
ATG3
MAPK8
TBC1D32
GCA
NEDD1
CHD5
TNPO1
GARS1
SRSF10
PPP2R2C
MATN2
PLCB4
CCDC43
B
log fold-cahnge MIG expression (siRNPC3 / siNT)
log2FC minor intron retention (siRNPC3 / siNT)
1.5
1
0.5
0
-0.5
-1
-1.5
-2
-1
2
3
4
CHD5
MATN3
PFDN5
ATG3
MAPK12
KIAA1328
TMEM258
PRIM1
COA3
CEP126
SPC24
significant changes in:
retention & DE
retention
DE

◀ **Figure EV6. A549 cells treated for 72 h with *RNPC3* siRNA exhibit differential expression of genes and MIGs that are enriched in cancer.**

(**A**) Heatmap of all identified differentially expressed MIGs, showing logCPM corrected for replicate and scaled for each gene. (**B**) Log-fold change plot of all MIGs highlighting those that exhibit significant changes in intron retention, differential expression or both when *RNPC3* is knocked down.

 © The Author(s)

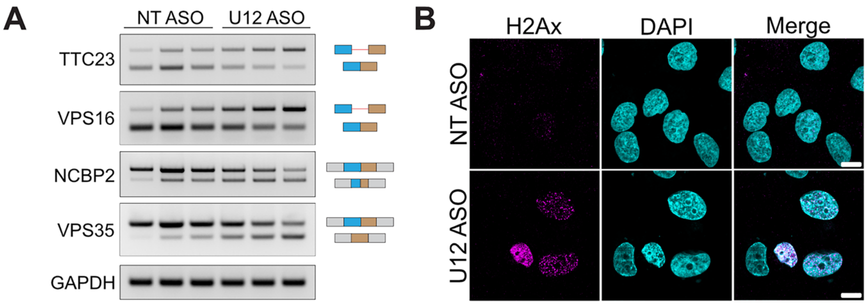

Figure EV7. A549 cells treated for 72 h with U12 ASO exhibit aberrant MIG splicing and markers of DNA damage.

(**A**) RT-PCR analysis of MIG splicing changes in A549 cells after 72 h treatment with 10 nM ASO. Schematic depictions of the obtained amplicons are shown on the right with the minor intron in red and the upstream and downstream exons coloured blue and orange, respectively. Exons not separated by a minor intron are grey. (**B**) Representative images of γH2Ax (Ser139) staining in A549 cells 72 h after 10 nM ASO treatment. Scale bar is 10 μm.

© The Author(s)
